# A Convergent Total Synthesis of (+)-Ineleganolide

**DOI:** 10.1021/jacs.3c02142

**Published:** 2023-03-29

**Authors:** Benjamin M. Gross, Seo-Jung Han, Scott C. Virgil, Brian M. Stoltz

**Affiliations:** † The Warren and Katharine Schlinger Laboratory for Chemistry and Chemical Engineering, California Institute of Technology, MC-101-20, Pasadena, California 91125, United States; ‡ Chemical and Biological Integrative Research Center, KIST and Division of Bio-Medical Science & Technology, KIST-School, UST, Seoul, 02792, Republic of Korea

## Abstract

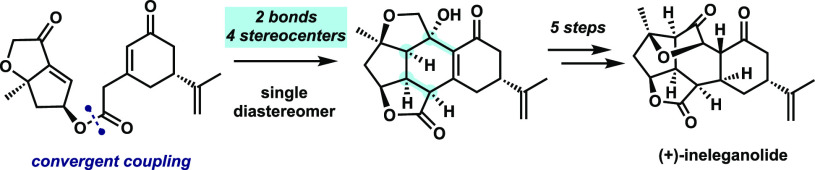

We report the total synthesis of the furanobutenolide-derived diterpenoid (+)-ineleganolide. The synthetic approach relies on a convergent strategy based on the coupling of two enantioenriched fragments, which are derived from (−)-linalool and (+)-norcarvone, respectively. A high-yielding, one-step Michael addition and aldol cascade furnishes a pentacyclic framework as a single diastereomer, thereby overcoming previous challenges in controlling stereochemistry. The endgame features an O_2_-facilitated C–H oxidation and a samarium diiodide-induced semipinacol rearrangement to furnish the highly rigid central seven-membered ring.

The cembranoid and norcembranoid diterpenoids represent a large family of natural products isolated from soft coral species.^1^ Because of their unique, highly complex structures, the furanobutenolide-derived diterpenoids have received considerable attention from synthetic chemists over the past decades, which has given rise to new reaction developments in synthetic chemistry.^2^ They have furthermore served as a proving ground in retrosynthetic planning and in delivering useful amounts of material for potential further investigations into their bioactivity.^3^ A subclass of these molecules contain a macrocyclic structure, as represented by the neurotoxin lophotoxin (**2**), which functions as an irreversible inhibitor of the nicotinic acetylcholine receptor (Figure 1).^4,5^ Biosynthetically, these macrocycles are suggested to engage in further modifications to give rise to more dense polycyclic structures. A showcase example of these architectures is reflected in bielschowskysin (**3**), which shows promising cytotoxicity against non-small cell lung cancer and renal cancer.^6^ Another flagship member of this class, ineleganolide (**1**), was isolated from the Formosan soft coral *Sinularia inelegans* by Duh and co-workers in 1999.^7^ It has shown preliminary cytotoxicity against P-380 leukeumia cell lines, but further insights into its bioactivity remain undisclosed. Structurally, ineleganolide contains a highly rigid oxidized framework that bears a key central seven-membered ring, a remote isopropenyl group, and a bridging β-keto tetrahydrofuran moiety. Because of its unique and challenging framework, the synthesis of ineleganolide had remained an unsolved challenge over the past two decades, despite efforts by the groups of Vanderwal,^8^ Nicolaou,^9^ Gaich,^10^ Romo,^11^ Moeller,^12^ and our group.^13^ Only recently, in 2022, did Wood and co-workers showcase the first total synthesis of ineleganolide.^14^ After elegantly constructing a macrocyclic precursor, they were able to form the last bond through a transannular Michael addition, similar to that disclosed by Pattenden and co-workers in their 2011 biomimetic semisynthesis of **1**,^15^ which gave rise to sinulochmodin C in 34.5% yield and ineleganolide in 11.5% yield, respectively.

**Figure 1:**
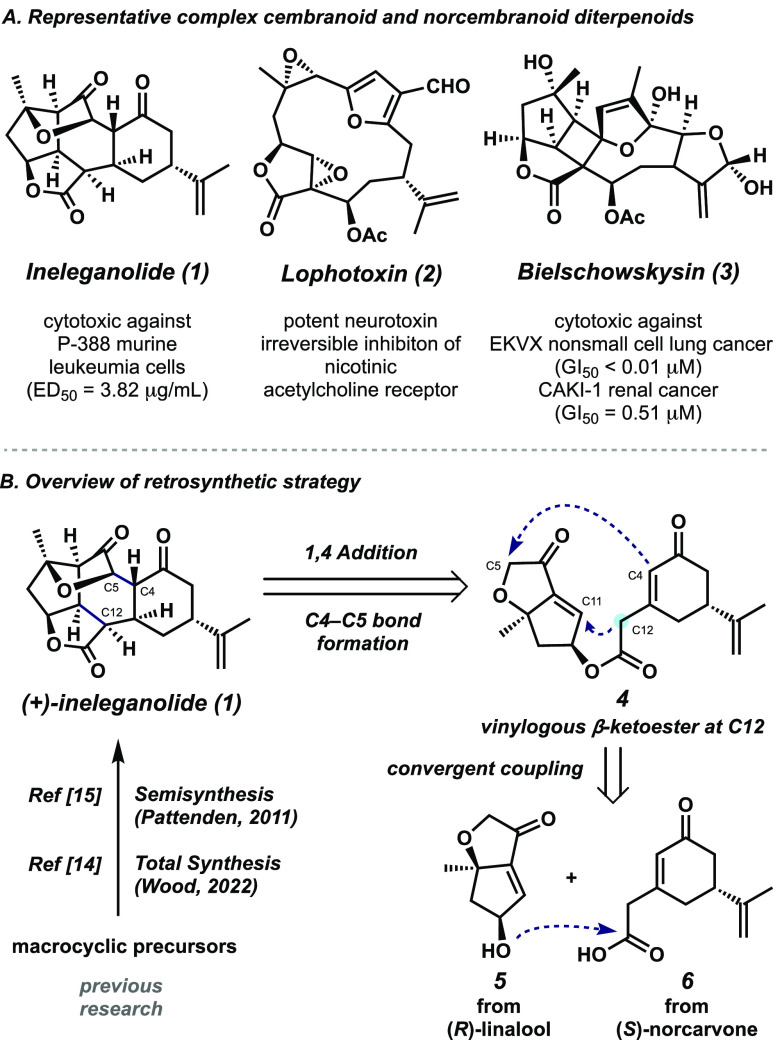
(A) Structures and bioactivity of representative furanobutenolide-derived diterpenoids. (B) Outline for the synthesis of (+)-ineleganolide.

In our own research, issues surrounding the central seven-membered ring and constructing the ether bridge at a late stage had halted previous efforts.^16,17^ This sparked the idea of constructing the central seven-membered ring at a later stage, and instead having the tetrahydrofuran motif introduced early. Therefore, we envisioned disconnecting through the C4–C5 bond as the final step and constructing the seven-membered ring last. Originally, we imagined this bond formation could potentially be achieved by a cross-enolate coupling process. Inspired by our previous research and, specifically, the work of Vanderwal and co-workers, we planned our route back to the precursor **4** to set the stereochemistry at C12 through a Michael addition.^18^ As a vinylogous β-keto ester, we envisioned that the lowered acidity of the C12 proton would thereby provide an epimerizable handle. Disconnecting **4** through an esterification, we were left with two fragments, carboxylic acid **6** and alcohol **5**. We were able to derive these from (−)-linalool and (+)-norcarvone, respectively, which resulted in an overall convergent and stereospecific synthesis.

To synthesize bicyclic enone **5**, we began from (*R*)-linalool (Scheme 1). Aldehyde **7** was readily available through previously developed chemistry established by the Maimone group and our group, respectively.^19,20^ This route proved to be highly scalable to access aldehyde **7** in sufficient quantities (>70 g prepared). We next focused our attention on building the essential β-keto tetrahydrofuran moiety present in bicycle **5**. This proved challenging because of the highly strained nature of enone **5**. Initially, extensive efforts of an intramolecular cyclization of the tertiary alcohol onto an α-functionalized ketone showed no success. We envisioned that oxidizing to the ketone later would alleviate the induced strain and allow for the intramolecular cyclization to occur. This idea proved to be successful, as the cyclization onto a β-functionalized secondary alcohol was possible. We converted aldehyde **7** to the corresponding epoxide **8** using dibromomethane as the one carbon source. Removal of both silyl groups by treatment of **8** with TBAF, followed by selective silylation of the resulting secondary alcohol with TBSCl, revealed the tertiary alcohol **9**. Treatment of epoxide **9** with magnesium diiodide revealed the intermediary iodohydrin **10**. Protection of the incipient secondary alcohol as the corresponding silyl ether proved necessary since cyclization in the presence of the unprotected secondary alcohol failed, even with excess amounts of base. Upon silylation, however, intramolecular substitution of the alkyl iodide by the tertiary alkoxide occurred at room temperature to afford bicycle **11** in excellent yield (98%). Selective deprotection of the triethyl silyl ether under mild acidic conditions gave alcohol **12**, which was oxidized to the enone under Swern conditions in 72% yield. Lastly, deprotection employing aqueous HF yielded **5** in 81% yield. Other deprotection conditions proved unsuccessful because of the presumed high reactivity and instability of enone **5** as an electrophilic Michael acceptor.

**Scheme 1:**
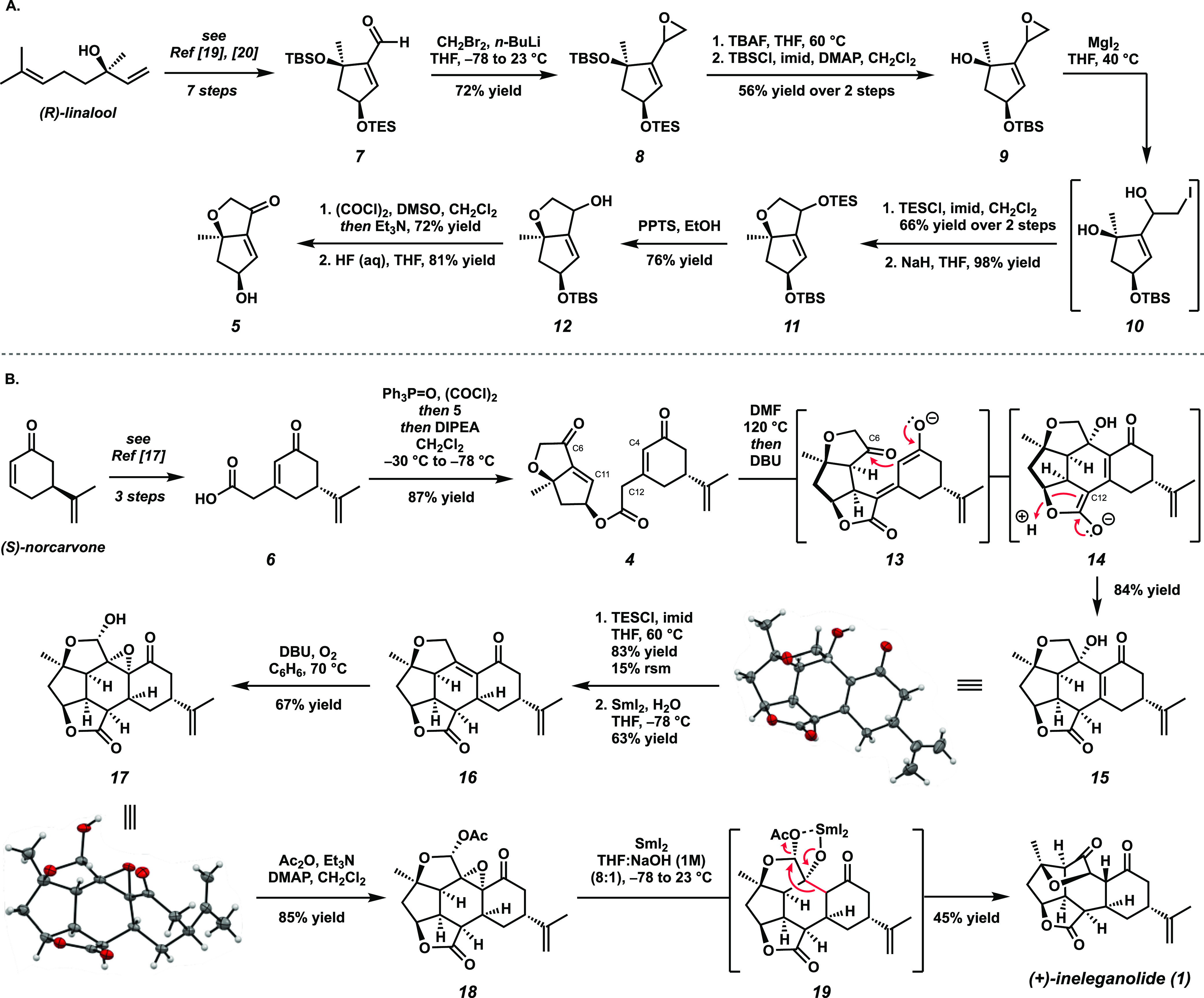
(A) Synthesis of Bicyclic Enone **5** and (B) Completed Synthesis of (+)-Ineleganolide **1**

Carboxylic acid **6** could be accessed through a protocol previously employed by our group, which utilizes (*S*)-norcarvone as an enantioenriched starting material.^17^ With acid **6** and alcohol **5** in hand, we turned to develop conditions for esterification. This proved challenging because of (1) the acidic α-proton of the acid **6** and (2) the instability of enone **5** toward an amine base or nucleophiles (Et_3_N, DIPEA, DMAP, pyridine, etc.), thereby leading to decomposition within minutes. As such, a broad range of esterification reagents were evaluated (i.e., EDC, DIC, Yamaguchi’s reagent, Otera’s catalyst, etc.) but failed to yield any desired product. We eventually developed specific conditions to overcome these issues by activating the acid as the triphenylphosphonium salt at low temperatures,^21^ which reacted with alcohol **5** within seconds upon addition of a base, to afford ester **4** in 87% yield. Next, we investigated the intramolecular Michael addition. While we were evaluating conditions, ester **4** appeared unstable to various bases and induced the formation of an array of unidentified side products upon reaction. To our surprise, treatment of ester **4** with DBU at 23 °C gave trace amounts of an unexpected pentacycle (**15**), the structure of which was determined by X-ray crystallography and resulted from not only Michael addition but a subsequent aldol cyclization, as well. We were able to optimize this process by preheating a solution of the ester **4** in DMF at high temperatures (i.e., 120 °C) and adding DBU in one portion. Under this protocol the reaction then proceeded within minutes to smoothly give **15** in 84% yield, thereby suppressing previous side products. Mechanistically, we believe that a Michael addition occurs first to form the C12–C11 bond. The second proton at C12 (i.e., **14**) can be abstracted again to give the conjugated enolate **13**. The extended enolate can then undergo aldol addition at C4 with the neighboring ketone and isomerize to give rise to intermediate **14**. Being sp^2^-hybridized, the enolate at C12 is preferentially protonated from the convex face, thereby giving **15** as a single diastereomer. Overall, this Michael addition and aldol cascade forges two bonds and four stereocenters as a single diastereomer in high yield, which crucially provides the correct stereochemistry at C12.

With this result in hand, we could envision a path toward ring expansion and completion of the natural product. To this end, protection of the tertiary alcohol as the silyl ether facilitated reduction of the tetrasubstituted enone under samarium diiodide conditions and subsequent elimination to enone **16**.^17,22^ While attempting an allylic oxidation at C5, we discovered that enone **16** undergoes a unique air oxidation under basic conditions to produce the epoxide hemiacetal **17** (Scheme 2). We were surprised by this nearly unprecedented reaction^23^ and speculate that triplet oxygen can facilitate a radical H atom abstraction to form the highly stabilized captodative radical **20** on the γ-position of the enone. Upon radical recombination, an initial peroxide can be formed, which can be deprotonated to form compound **21**.

**Scheme 2:**
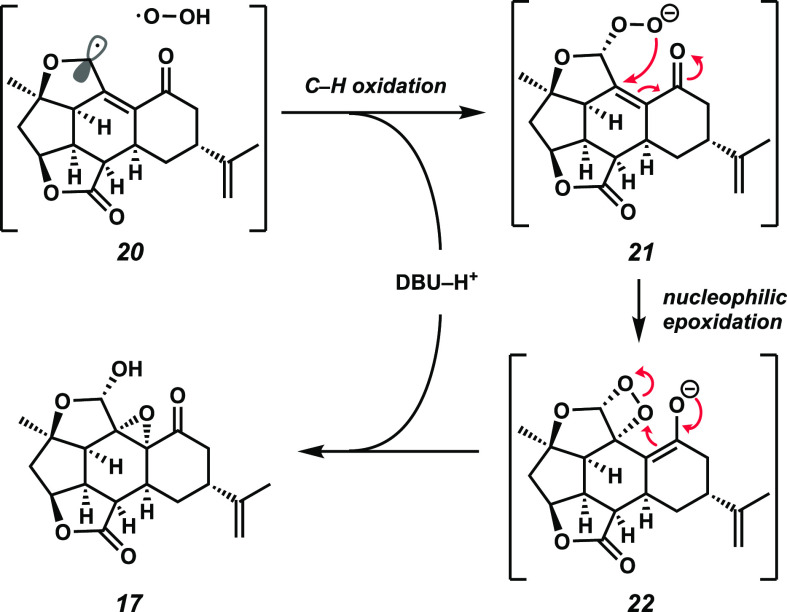
Suggestive Mechanism for the Formation of **17**

Under basic conditions, the peroxyanion **21** can then undergo intramolecular nucleophilic epoxidation with the enone to give intermediate **22**, which converts to the final product **17**. After optimization, **17** could be reliably obtained in 67% yield because it is highly sensitive to the stirring rate and oxygen atmosphere. Having the correct oxidation pattern in place, we planned to construct the crucial C4–C5 bond through reductive opening of the epoxide and a subsequent semipinacol shift. We deemed the odds in our favor and predicted good antiperiplanar orbital overlap between the shifting carbon–carbon bond and hemiacetal leaving group. Additionally, the ability to form a stabilized oxocarbenium ion could also be beneficial. Initial investigations showed that epoxide opening typically leads to elimination of the resulting tertiary alcohol.^24^ Conversion of hemiacetal **17** to the acetate **18** provided the initial lead, and reductive opening of **18** with samarium diiode provided trace amounts of ineleganolide, which indicates that the semipinacol rearrangement proceeded in the same pot as the epoxide opening. We then extensively evaluated additives and temperatures and found that addition of an aqueous 1 M sodium hydroxide solution as relatively high pH proton source significantly improved the yield. Although a number of mechanistic scenarios are possible, we deemed the existence of intermediate **19** to be crucial, with samarium potentially engaging as a Lewis acid to promote the rearrangement.^25^ The basicity of the proton source could provide improved conditions to protonate the initially forming samarium enolate without protonating the tertiary alcohol too rapidly and forcing elimination. Quickly warming up from −78 to 23 °C gave (+)-ineleganolide **1** in 45% isolated yield. While the isolation paper initially provides an X-ray structure of ineleganolide, lower resolution prevented determination of absolute stereochemistry.^7^ This led us to obtain a higher-resolution crystal structure that further confirms the absolute stereochemistry of the naturally occurring enantiomer (+)-ineleganolide, in accordance with observations by Wood^14^ and Pattenden^15^ (Figure 2).

**Figure 2:**
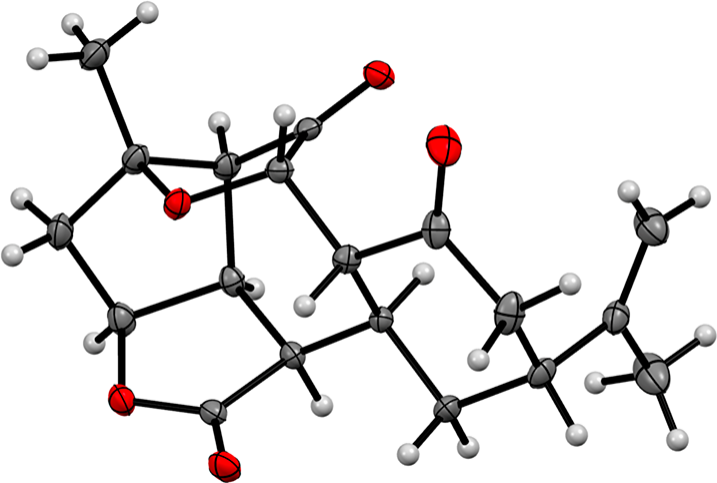
X-ray diffraction structure of (+)-ineleganolide.

In conclusion, we have completed the total synthesis of (+)-ineleganolide in an overall longest linear sequence of 23 steps from (−)-linalool. We based our convergent strategy on the union of two fragments, thereby providing a concise endgame with only seven total steps from our coupling partners **5** and **6**. We were able to access a highly strained enone **5** and develop underutilized esterification conditions for sensitive substrates by employing triphenyl phosphine oxide and oxalyl chloride as activating reagents. Furthermore, we realized an exceptional Michael addition and aldol cascade by constructing a crucial pentacyclic intermediate as a single diastereomer (i.e., **4** → **15**). In the later stage, we discovered a unique air oxidation and epoxidation sequence to install the needed oxidation pattern (i.e., **16** → **17**). Reductive opening of acetoxy epoxide **18** with samarium diiode induced a semipinacol shift in the same pot to furnish (+)-ineleganolide (**1**) in good yield. Future efforts will be directed toward shortening the overall step count, particularly toward the development of a more concise route to enone **5**. However, our present sequence has proved to be scalable and reliable. Additionally, we envision that the developed chemistry can be utilized in solving future problems in the synthesis of related compounds and enable further investigations into the bioactivity of the cembranoid and norcembranoid diterpenoids, specifically (+)-ineleganolide.

## Supporting Information Available

Experimental procedures and spectroscopic data (^1^H NMR, ^13^C NMR, IR, and HRMS) (PDF)
