# Supplementary material for: A Convergent Total Synthesis of (+)-Ineleganolide
Source: J Am Chem Soc. 2023 Mar 29;145(14):7763–7. doi: 10.1021/jacs.3c02142 (PMC10544024; doi:10.1021/jacs.3c02142)
Supplement: Supplementary file 1 — ja3c02142_si_001.pdf [file ja3c02142_si_001.pdf]

A Convergent Total Synthesis of (+)-Ineleganolide

Benjamin M. Gross, Seo-Jung Han, Scott C. Virgil, and Brian M. Stoltz*

Warren and Katharine Schlinger Laboratory of Chemistry and Chemical Engineering, Division of Chemistry and Chemical Engineering, California Institute of Technology, MC 101-20, Pasadena, California 91125, United States

stoltz@caltech.edu

Table of Contents:

Materials and Methods 2
List of Abbreviations 2
Synthesis of Starting Materials 3
Experimental Procedures 4
Comparison of Natural and Synthetic Ineleganolide 18
Spectral Data 22
X-ray Crystal Structure Data 50

Materials and Methods

Unless otherwise stated, reactions were performed in flame-dried glassware under an argon or nitrogen atmosphere using dry, deoxygenated solvents. Solvents were dried by passage through an activated alumina column under argon.[1] Reaction progress was monitored by thin-layer chromatography (TLC). TLC was performed using E. Merck silica gel 60 F254 precoated glass plates (0.25 mm) and visualized by UV fluorescence quenching, p-anisaldehyde, or $KMnO_4$ staining. Silicycle SiliaFlash® P60 Academic Silica gel (particle size 40–63 μm) was used for flash chromatography. ^{1}H NMR spectra were recorded on Varian Inova 500 MHz and 600 MHz and Bruker 400 MHz spectrometers and are reported relative to residual $CHCl_3$ (δ 7.26 ppm), C_6D_6 (δ 7.16 ppm) or CD3OD (δ 3.31 ppm). ^{13}C NMR spectra were recorded on a Varian Inova 500 MHz spectrometer (125 MHz) and Bruker 400 MHz spectrometers (100 MHz) and are reported relative to $CHCl_3$ (δ 77.16 ppm), C_6D_6 (δ 128.06 ppm) or CD_3OD (δ 49.01 ppm). Data for ^{1}H NMR are reported as follows: chemical shift (δ ppm) (multiplicity, coupling constant (Hz), integration). Multiplicities are reported as follows: s = singlet, d = doublet, t = triplet, q = quartet, p = pentet, sept = septuplet, m = multiplet, br s = broad singlet, br d = broad doublet. Data for ^{13}C NMR are reported in terms of chemical shifts (δ ppm). IR spectra were obtained by use of a Perkin Elmer Spectrum BXII spectrometer or Nicolet 6700 FTIR spectrometer using thin films deposited on NaCl plates and reported in frequency of absorption (cm^{-1}). Optical rotations were measured with a Jasco P-2000 polarimeter operating on the sodium D-line (589 nm), using a 100 mm path-length cell. High resolution mass spectra (HRMS) were obtained using an Agilent 6200 Series TOF with an Agilent G1978A Multimode source in electrospray ionization (ESI+) mode.

List of Abbreviations

TBAF – Tetra-n-butylammonium fluoride, DMAP – 4-Dimethylaminopyridine, PPTS – Pyridinium p-toluenesulfonate, HPLC – High performance liquid chromatography, DBU – 1,8-Diazabicyclo[5.4.0]undec-7-ene, DIPEA – Diisopropylethylamine

Synthesis of Starting Materials

Scheme S1: Route to Aldehyde **7**, *Ref. [2] and Ref. [3].*

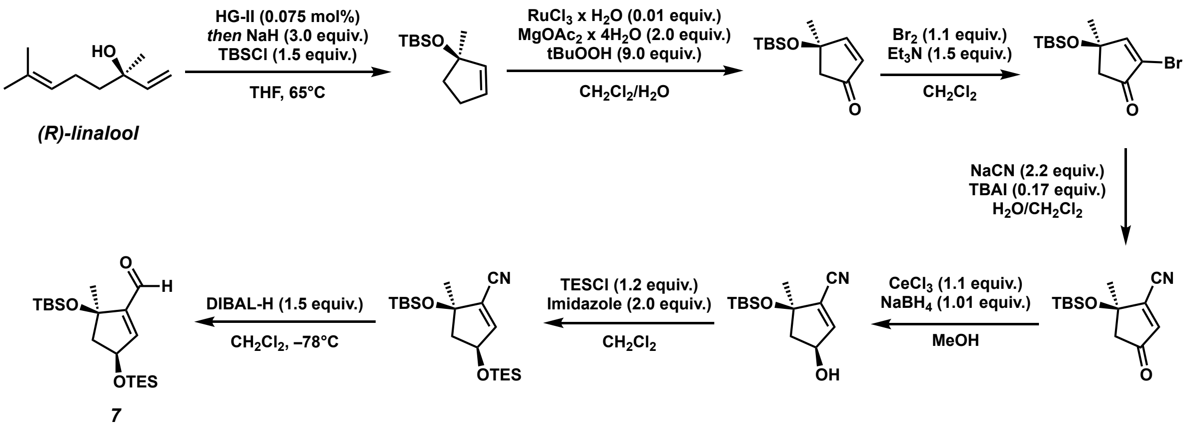

Scheme S2: Route to Carboxylic Acid **6**, *Ref. [4].*

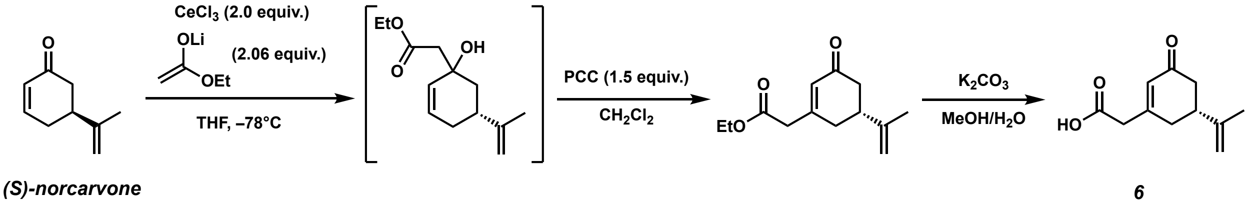

Experimental Procedures

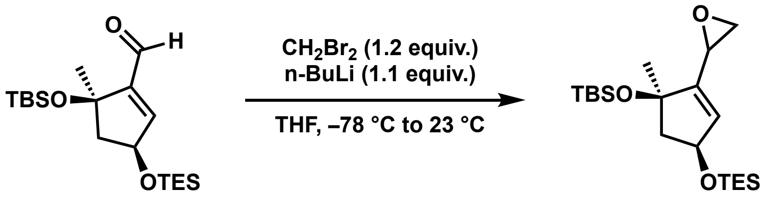

7 ***8***

Epoxide 8: In a flame dried round-bottom flask, Aldehyde **7** (9.4 g, 25.3 mmol, 1.0 equiv.) was dissolved in THF (125 mL, 0.2 M) and CH_2Br_2 (2.1 mL, 30.1 mmol, 1.2 equiv.) was added in one portion. The solution was cooled to –78 °C in a dry ice/acetone bath and *n*-BuLi (2.5 M in hexanes, 10.6 mL, 27.6 mmol, 1.1 equiv.) was added dropwise over the course of one hour. The reaction mixture was left to stir in the dry ice/acetone bath and let warm to 23 °C overnight (12 h). After consumption of starting material as determined by TLC, saturated aqueous NH_4Cl was added, the layers separated, and the aqueous layer was extracted with EtOAc (3x). The combined organic extracts were dried over Na_2SO_4, and the solvent evaporated under reduced pressure. The crude product was purified by flash chromatography on silica gel (hexanes → 5% Et_2O/hexanes) to afford the title compound **8** as a pale-yellow oil (7.0 g, 18.2 mmol, 72% yield). ***8*** *was isolated as an inconsequential mixture of diastereomers (ratio ~2:1). Signals for the major diastereomer are reported*; ^{1}H NMR (600 MHz, $CDCl_3$) δ 5.47 (s, 1H), 4.62 – 4.58 (m, 1H), 3.37 (s, 1H), 2.98 – 2.95 (m, 1H), 2.74 (dd, *J* = 6.0, 2.7 Hz, 1H), 2.44 (dd, *J* = 12.2, 6.8 Hz, 1H), 1.95 (dd, *J* = 12.2, 6.5 Hz, 1H), 1.38 (s, 3H), 0.95 (t, *J* = 7.9 Hz, 9H), 0.86 (s, 9H), 0.60 (q, *J* = 7.9 Hz, 6H), 0.11 (s, 3H), 0.09 (s, 3H); ^{13}C NMR (100 MHz, $CDCl_3$) δ 149.8, 127.3, 82.1, 73.3, 53.1, 49.9, 47.0, 28.6, 25.8, 18.0, 6.9, 4.8, -2.0, -2.4; IR (neat film, NaCl) 2955, 2885, 2855, 1471, 1461, 1359, 1251, 1118, 1004, 834, 739, 671, 645 cm^{-1}; HRMS (ESI+): m/z calc'd for $C_{14}H_{25}SiO_2$ [M–OTBS]$^+$: 253.1618, found 253.1610; $[\alpha]_D^{23}$ + 7.7 (*c* 0.20, $CHCl_3$).

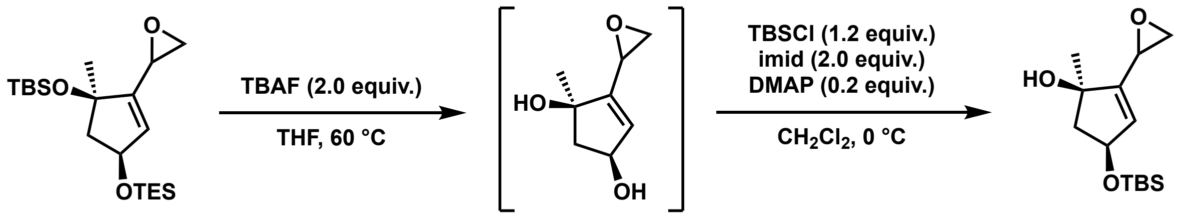

8 ***23*** ***9***

Tertiary Alcohol 9: A round-bottom flask was charged with **8** (12.1 g, 31.4 mmol, 1.0 equiv.) and dissolved in THF (314 mL, 0.1 M). TBAF (1M in THF, 62.9 mL, 2.0 equiv.) was added and the solution heated to 60 °C. After 3h, full consumption of starting material was observed by TLC and the solution was left to cool to 23 °C. The reaction mixture was diluted with hexanes (50 mL) and flushed over a plug of silica gel, eluting with a mixture of EtOAc/Hexanes (9:1). The solvent was evaporated under reduced pressure, and the crude diol **23** (3.8 g) isolated as a yellow oil which was directly used in the next step.

Diol **20** (3.8 g, 24.3 mmol, 1.0 equiv.) was dissolved in CH_2Cl_2 (243 mL, 0.1 M), and imidazole (3.3 g, 48.6 mmol, 2.0 equiv.) added in one portion. The solution was cooled to 0°C, after which TBSCl (4.4 g, 29.2 mmol, 1.2 equiv.) and DMAP (0.59 g, 4.86 mmol, 0.2 equiv.) were subsequently added. The solution was stirred for 1 h, quenched with a saturated aqueous solution of $NaHCO_3$, and the aqueous layer extracted with EtOAc (3x). The combined organic extracts were washed with brine, dried over Na_2SO_4 and the solvent evaporated under reduced pressure. The crude product was purified by flash chromatography on silica gel (20% EtOAc/hexanes), to yield tertiary alcohol **9** (4.8 g, 17.7 mmol, 56% over two steps) as a yellow oil. ***9*** *was isolated as an inconsequential mixture of diastereomers (ratio ~2:1). Signals for the major diastereomer are reported*; ^{1}H NMR (600 MHz, $CDCl_3$) δ 5.68 (s, 1H), 4.63 – 4.58 (m, 1H), 3.47 (s, 1H), 3.02 – 2.98 (m, 1H), 2.87 – 2.82 (m, 1H), 2.41 (dd, J = 13.3, 6.5 Hz, 1H), 2.21 – 2.13 (m, 1H), 1.90 – 1.83 (m, 1H), 1.41 (s, 3H), 0.88 (s, 9H), 0.07 (s, 6H); ^{13}C NMR (100 MHz, $CDCl_3$) δ 148.1, 131.1, 81.0, 73.2, 52.7, 49.4, 47.4, 25.9, 18.2, -4.5, -4.5; IR (neat film, NaCl) 2956, 2855, 1614, 1460, 1422, 1360, 1252, 1166, 1079, 1064, 898, 834, 776, 759, 748, 662 621 cm^{-1}; HRMS (ESI+): m/z calc'd for $C_{14}H_{25}SiO_2$ [M–OH]$^+$: 253.1618, found 253.1611; $[\alpha]_D^{25}$ + 11.5 (c 0.15, $CHCl_3$).

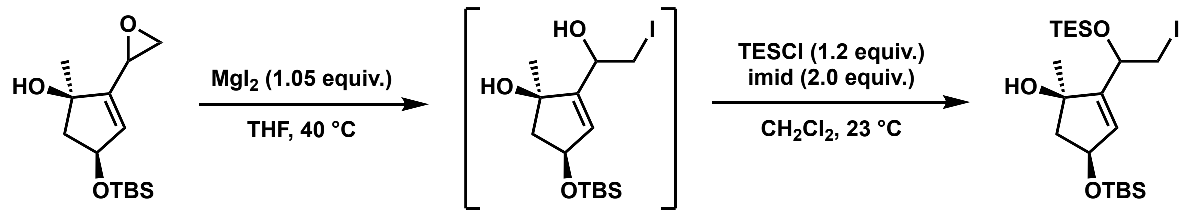

9 ***10*** ***24***

Alkyliodide 24: A flame dried round-bottom flask was charged with **9** (4.8 g, 17.7 mmol, 1.0 equiv.) and transferred into a glovebox. The starting material was dissolved in THF (89 mL, 0.2 M) and anhydrous MgI_2 (5.17 g, 18.6 mmol, 1.05 equiv.) was added in one portion. The flask was sealed with a rubber septum and transferred outside the glovebox. The flask was kept under N_2, placed in a heating mantel set to 40 °C and the reaction mixture stirred for 14 h. H_2O was added, and the aqueous phase extracted with EtOAc (3x). The combined organic extracts were washed with brine, dried over Na_2SO_4 and the solvent evaporated under reduced pressure to yield the crude diol **10** (7.05 g) as a yellow oil, which was directly used in the next step.

Diol **10** (7.05 g, 17.7 mmol, 1.0 equiv.) was dissolved in CH_2Cl_2 (177 mL, 0.1 M), imidazole (2.4 g, 35.4 mmol, 2.0 equiv.) added in one portion and the reaction mixture cooled to 0 °C. TESCl (3.5 mL, 21.2 mmol, 1.2 equiv.) was added dropwise and the reaction mixture stirred for 10 minutes. Brine was added, the phases separated, and the aqueous phase extracted with Et_2O (3x). The combined organic extracts were dried over Na_2SO_4, and the solvent evaporated under reduced pressure. The crude product was purified by flash chromatography on silica gel (hexanes → 10% Et_2O/hexanes) to yield alkyliodide **24** (5.94 g, 11.6 mmol, 66% over two steps) as a yellow oil. ***24*** *was isolated as an inconsequential mixture of diastereomers. Signals for the major diastereomer are reported*; ^1H NMR (600 MHz, $CDCl_3$) δ 5.89 (s, 1H), 4.58 – 4.55 (m, 1H), 4.31 – 4.28 (m, 1H), 3.59 (dd, J = 10.2, 3.9 Hz, 1H), 3.35 (dd, J = 10.1, 6.4 Hz, 1H), 2.23 (dd, J = 13.2, 6.0 Hz, 1H), 1.88 (dd, J = 13.2, 3.0 Hz, 1H), 1.33 (s, 3H), 0.97 (t, J = 7.9 Hz, 9H), 0.88 (s, 9H), 0.66 – 0.58 (m, 6H), 0.08 (s, 6H); ^{13}C NMR (100 MHz, $CDCl_3$) δ 151.7, 132.3, 80.5, 72.7, 69.1, 52.5, 25.9, 25.3, 18.2, 15.3, 7.0, 5.0, -4.3, -4.6; IR (neat film, NaCl) 2956, 2929, 2879, 1362, 1098, 1004, 834, 736, 652 cm^{-1}; HRMS (ESI+): m/z calc'd for $C_{20}H_{40}ISi_2O_2$ $[M–OH]^+$: 495.1606, found 495.1600; $[\alpha]_D^{25}$ + 4.31 (c 0.20, $CHCl_3$).

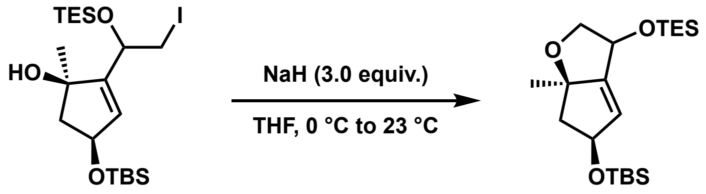

24 ***11***

Bicycle 11: A flame dried round-bottom flask was charged with alkyl iodide **24** (5.94 g, 11.59 mmol, 1.0 equiv.), dissolved in THF (0.013 M, 891 mL) and cooled to 0°C. NaH (60% in mineral oil, 1.4 g, 34.7 mmol, 3.0 equiv.) was added portion wise, after which the ice bath was removed and the reaction stirred at 23°C for 2.5 hours, until full consumption of starting material as determined by TLC. The reaction was quenched with a saturated aqueous solution of NH_4Cl, and the aqueous layer extracted with EtOAc (3x). The combined organic extracts were dried over Na_2SO_4, and the solvent evaporated under reduced pressure. The crude product was purified by flash chromatography on silica gel (hexanes → 10% Et_2O/hexanes) to yield the title compound **11** (4.4 g, 11.4 mmol, 98%) as a colorless oil; ***11** was isolated as an inconsequential mixture of diastereomers. Signals for the major diastereomer are reported*; ^{1}H NMR (600 MHz, $CDCl_3$) δ 5.61 (s, 1H), 5.02 (t, *J* = 6.3 Hz, 1H), 4.62 (d, *J* = 3.1 Hz, 1H), 4.11 (dd, *J* = 9.5, 4.7 Hz, 1H), 4.00 (dd, *J* = 9.6, 1.8 Hz, 1H), 2.44 (dd, *J* = 11.6, 5.5 Hz, 1H), 1.87 (dd, *J* = 11.6, 7.4 Hz, 1H), 1.39 (s, 3H), 0.95 (t, *J* = 7.9 Hz, 9H), 0.89 (s, 9H), 0.60 (q, *J* = 7.9 Hz, 6H), 0.09 (s, 3H), 0.07 (s, 3H); ^{13}C NMR (125 MHz, $CDCl_3$) δ 151.3, 128.7, 90.0, 79.3, 78.2, 68.1, 54.5, 26.0, 24.9, 18.3, 6.8, 4.8, -4.4, -4.6; IR (neat film, NaCl) 2955, 2878, 2856, 1463, 1456, 1362, 1251, 1102, 1051, 1003, 895, 862, 831, 779, 728 cm^{-1}; HRMS (ESI+): m/z calc'd for $C_{20}H_{41}Si_2O_3$ $[M+H]^+$: 385.2589, found 385.2579; $[\alpha]_D^{25}$ + 12.7 (*c* 0.15, $CHCl_3$).

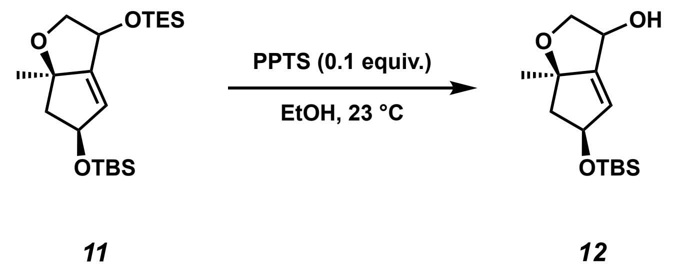

Allylic alcohol 12: A round-bottom flask was charged with **11** (2.25g, 5.85 mmol, 1.0 equiv.) and dissolved in EtOH (58 mL, 0.1 M). PPTS (147.0 mg, 0.585 mmol, 0.1 equiv.) was added in one portion and the reaction mixture stirred for 1 h. After nearly full consumption of starting material as determined by TLC, a saturated aqueous solution of $NaHCO_3$ was added. The aqueous layer was extracted with EtOAc (3x), the combined organic extracts dried over Na_2SO_4, and the solvent evaporated under reduced pressure. The crude product was purified by flash chromatography on silica gel (hexanes → 20% EtOAc/hexanes) to yield the title compound **12** (1.2 g, 4.4 mmol, 76%) as a colorless oil; ***12** was isolated as an inconsequential mixture of diastereomers. Signals for the major diastereomer are reported;* ^{1}H NMR (600 MHz, $CDCl_3$) δ 5.74 (s, 1H), 5.03 (t, J = 6.5 Hz, 1H), 4.73 (dd, J = 4.8, 1.9 Hz, 1H), 4.15 (dd, J = 10.0, 4.9 Hz, 1H), 4.06 (dd, J = 10.1, 1.8 Hz, 1H), 2.45 (dd, J = 11.6, 5.5 Hz, 1H), 1.93 (s, 1H), 1.90 (dd, J = 11.6, 7.5 Hz, 1H), 1.41 (s, 3H), 0.89 (s, 9H), 0.09 (s, 3H), 0.07 (s, 3H); ^{13}C NMR (125 MHz, $CDCl_3$) δ 150.7, 130.2, 89.9, 79.3, 77.2, 68.0, 54.7, 25.9, 25.1, 18.3, -4.4, -4.6; IR (neat film, NaCl) 3402, 2931, 2857, 1471, 1360, 1260, 1213, 1185, 1097, 1047, 977, 828, 775, 674 cm^{-1}; HRMS (ESI+): m/z calc'd for $C_{14}H_{27}SiO_3$ $[M+H]^+$: 271.1724, found 271.1719; $[\alpha]_D^{25}$ + 33.7 (*c* 0.06, $CHCl_3$).

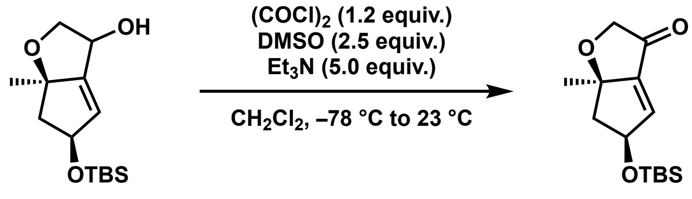

Enone 25: In a flame dried round-bottom, a solution of DMSO (0.78 mL, 11.0 mmol, 2.5 equiv.) in CH_2Cl_2 (44.3 mL) was cooled to –78 °C and oxalyl chloride (0.45 mL, 5.31 mmol, 1.2 equiv.) was added dropwise. After stirring at the same temperature for 15 minutes, alcohol **12** (1.2 g, 4.4 mmol, 1.0 equiv.) was added as a solution in CH_2Cl_2 (44.3 mL). The reaction mixture was stirred for 1 hour at –78 °C, after which Et_3N (3.1 mL, 22.1 mmol, 5.0 equiv.) was added and the reaction mixture let warm to 23 °C. The solvent was evaporated under reduced pressure and the crude mixture purified by flash chromatography on silica gel (hexanes → 10% EtOAc/hexanes). The title compound **25** (854 mg, 3.18 mmol, 72%) was obtained as a colorless oil; ^{1}H NMR (600 MHz, $CDCl_3$) δ 6.48 (s, 1H), 5.18 (t, J = 6.6 Hz, 1H), 4.26 (d, J = 17.1 Hz, 1H), 4.21 (d, J = 17.1 Hz, 1H), 2.64 (dd, J = 11.8, 5.6 Hz, 1H), 2.27 (dd, J = 11.9, 8.0 Hz, 1H), 1.39 (s, 3H), 0.90 (s, 9H), 0.11 (s, 3H), 0.10 (s, 3H); ^{13}C NMR (100 MHz, $CDCl_3$) δ 200.1, 146.4, 138.8, 90.5, 80.0, 74.0, 55.6, 25.8, 22.9, 18.2, -4.5, -4.7; IR (neat film, NaCl) 2955, 2930, 2857, 1732, 1645, 1362, 1258, 1103, 1035, 865, 834, 779, 651 cm^{-1}; HRMS (ESI+): m/z calc'd for $C_{14}H_{25}SiO_3$ $[M+H]^+$: 269.1567, found 269.1561; $[\alpha]_D^{23}$ – 48.0 (c 0.50, $CHCl_3$).

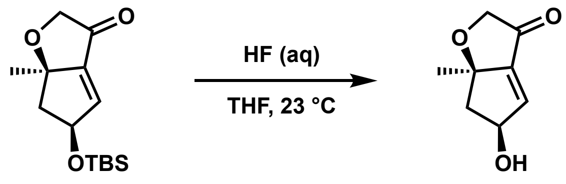

25 5

Alcohol 5: A round-bottom flask was charged with **25** (420 mg, 1.56 mmol, 1.0 equiv.) and dissolved in THF (15 mL, 0.1 M), after which an aqueous solution of HF (45%, 0.6 mL) was added dropwise. After stirring at 23 °C for 5 hours, the reaction was quenched with a saturated aqueous solution of $NaHCO_3$. The aqueous layer was extracted with EtOAc (3x), the combined organic extracts dried over Na_2SO_4, and the solvent evaporated under reduced pressure. The crude product was purified by flash chromatography on silica gel (30% EtOAc/hexanes) to yield the title compound **5** (196 mg, 1.27 mmol, 81%) as a white solid; ^{1}H NMR (600 MHz, $CDCl_3$) δ 6.55 (s, 1H), 5.22 (t, *J* = 6.9 Hz, 1H), 4.28 (d, *J* = 17.2 Hz, 1H), 4.23 (d, *J* = 17.2 Hz, 1H), 2.78 (dd, *J* = 12.0, 5.7 Hz, 1H), 2.20 (dd, *J* = 12.1, 8.0 Hz, 1H), 1.40 (s, 3H); ^{13}C NMR (100 MHz, $CDCl_3$) δ 200.0, 147.3, 137.6, 90.7, 79.9, 74.0, 55.0, 22.7; IR (neat film, NaCl) 3435, 2921, 2852, 1749, 1220 cm^{-1}; HRMS (ESI+): m/z calc'd for $C_8H_{11}O_3$ $[M+H]^+$: 155.0703, found 155.0700; $[\alpha]_D^{23}$ –15.0 (*c* 0.30, $CHCl_3$).

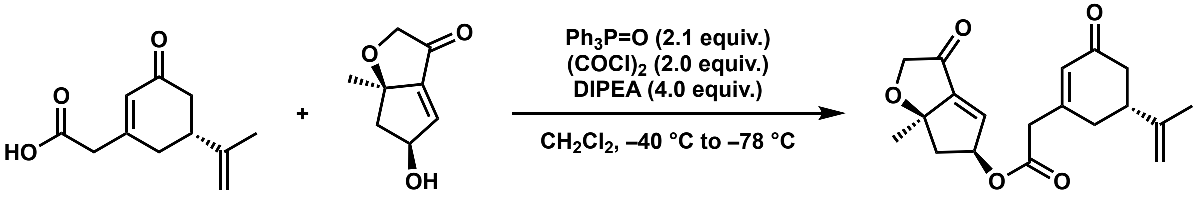

6 **5** **4**

Ester 4: A flame dried round-bottom flask was charged with triphenylphosphine oxide (478 mg, 1.7 mmol, 2.1 equiv.) and dissolved in CH_2Cl_2 (3.3 mL). The reaction mixture was cooled to –10 °C using a dry ice/acetone bath, and oxalyl chloride (0.14 mL, 1.65 mmol, 2.0 equiv.) was added dropwise. After stirring at the same temperature for 10 minutes, the reaction mixture was cooled to –40 °C, after which acid **6** (323 mg, 1.65 mmol, 2.0 equiv.) was added dropwise as a solution in CH_2Cl_2 (1.0 mL). The mixture was stirred for exactly 10 minutes, while the temperature of the dry ice/acetone bath was monitored with a thermostat and kept between –35 °C to –40 °C. Subsequently, the reaction mixture was cooled to –78 °C, after which alcohol **5** (127 mg, 0.8 mmol, 1.0 equiv.) was added as a solution in CH_2Cl_2 (1.0 mL). Following directly, a solution of diisopropylethylamine (0.53 mL, 3.3 mmol, 4.0 equiv.) in CH_2Cl_2 (0.5 mL) was added dropwise to the reaction mixture and stirred at –78 °C for 10 minutes. The reaction mixture was quenched at the same temperature with a saturated aqueous solution of NH_4Cl and let warm to 23 °C. The aqueous layer was extracted with EtOAc (3x), the combined organic extracts dried over Na_2SO_4, and the solvent evaporated under reduced pressure. The crude product was purified by flash chromatography on silica gel (30% EtOAc/hexanes) to yield the title compound **4** (235 mg, 0.71 mmol, 87%) as a colorless oil; ^{1}H NMR (600 MHz, $CDCl_3$) δ 6.45 (s, 1H), 6.04 (t, J = 7.1 Hz, 1H), 5.96 (s, 1H), 4.83 (s, 1H), 4.77 (s, 1H), 4.31 (d, J = 17.3 Hz, 1H), 4.25 (d, J = 17.2 Hz, 1H), 3.29 (s, 2H), 2.81 (dd, J = 12.2, 6.0 Hz, 1H), 2.72 (ddt, J = 14.1, 9.6, 4.9 Hz, 1H), 2.54 (dd, J = 16.3, 3.9 Hz, 1H), 2.46 – 2.39 (m, 2H), 2.35 – 2.28 (m, 2H), 1.76 (s, 3H), 1.44 (s, 3H); ^{13}C NMR (100 MHz, $CDCl_3$) δ 199.1, 198.8, 168.6, 155.4, 149.3, 146.0, 132.4, 128.9, 111.2, 90.5, 81.5, 74.3, 50.9, 43.0, 42.2, 41.9, 34.9, 22.9, 20.6; IR (neat film, NaCl) 2976, 1660, 1136, 1028, 988, 889, 735, 658 cm^{-1}; HRMS (ESI+): m/z calc'd for $C_{19}H_{23}O_5$ [M+H]$^+$: 331.1540, found 331.1535; $[\alpha]_D^{23}$ – 91.6 (*c* 0.50, $CHCl_3$).

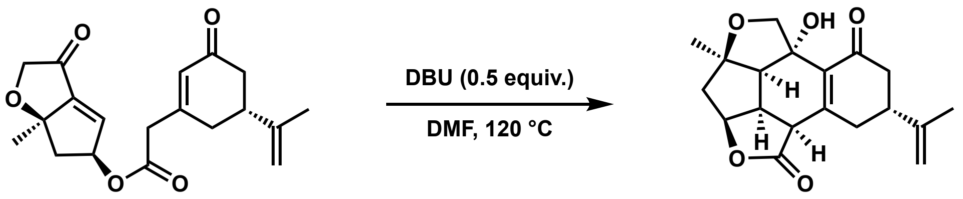

4 **15**

Tertiary Alcohol 15: In a flame dried round-bottom flask, **4** (228 mg, 0.69 mmol, 1.0 equiv.) was dissolved in dry DMF (69 mL, 0.01 M) under N_2 atmosphere and heated in a heating mantel set to 120°C for 10 minutes. DBU (51.6 µL, 0.34 mmol, 0.5 equiv.) was added in one portion via syringe and the reaction mixture stirred for 5 minutes. The reaction mixture was consequently cooled to 23°C and a saturated aqueous solution of NH_4Cl and EtOAc was added. The phases were separated, and the aqueous layer extracted with EtOAc (3x). The combined organic extracts were washed with a 10% aqueous solution of LiCl (3x), then brine, dried over Na_2SO_4 and the solvent evaporated under reduced pressure. The crude product was purified via flash chromatography on silica gel (50% EtOAc/hexanes) and the title compound **15** (193 mg, 0.58 mmol, 84%) isolated as a white solid. ^{1}H NMR (600 MHz, $CDCl_3$) δ 5.52 (s, 1H), 5.03 (t, *J* = 6.1 Hz, 1H), 4.85 (s, 1H), 4.79 (s, 1H), 4.20 (d, *J* = 9.9 Hz, 1H), 3.62 (d, *J* = 10.0 Hz, 1H), 3.53 (d, *J* = 9.8 Hz, 1H), 3.26 (ddd, *J* = 12.3, 9.9, 6.4 Hz, 1H), 3.06 (dd, *J* = 18.0, 4.7 Hz, 1H), 2.84 (ddt, *J* = 14.8, 9.0, 4.2 Hz, 1H), 2.63 – 2.51 (m, 4H), 2.50 – 2.41 (m, 1H), 2.01 (dd, *J* = 15.9, 5.8 Hz, 1H), 1.78 (s, 3H), 1.55 (s, 3H); ^{13}C NMR (100 MHz, $CDCl_3$) δ 201.9, 173.9, 151.4, 145.7, 134.8, 111.3, 95.0, 85.7, 83.5, 80.6, 59.7, 46.1, 44.4, 43.7, 41.2, 40.3, 36.4, 24.2, 20.5; IR (neat film, NaCl) 3458, 3082, 2971, 1755, 1655, 1360, 1114, 1062, 897, 733 cm^{-1}; HRMS (ESI+): m/z calc'd for $C_{19}H_{21}O_4$ [M–OH]$^+$: 313.1434, found 331.1435; $[\alpha]_D^{23}$ + 14.3 (*c* 1.00, $CHCl_3$).

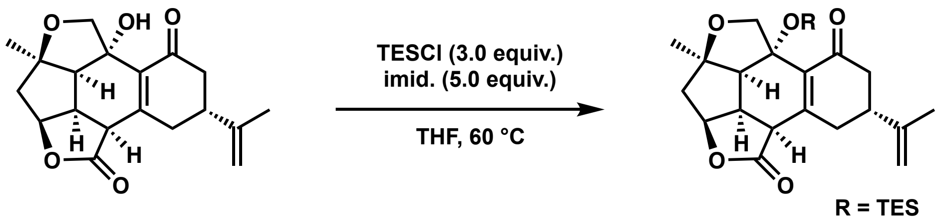

15 ***26***

Silyl ether 26: In a vial, **15** (100 mg, 0.3 mmol, 1.0 equiv.) was dissolved in THF (3 mL, 0.1 M) and imidazole (102 mg, 1.5 mmol, 5.0 equiv.) added in one portion. Subsequently, TESCl (152 µL, 0.9 mmol, 3.0 equiv.) was added dropwise. The vial was sealed with a plastic cap and placed in a heating block set to 60 °C. After stirring for exactly 3 h, the reaction mixture was flushed over a plug of silica gel, eluting with EtOAc. After the solvent was removed under reduced pressure, the crude product was purified by flash chromatography on silica gel (20% EtOAc/hexanes → 60% EtOAc/hexanes) to give the title compound **26** (112 mg, 0.25 mmol, 83%) as a white solid, along with reisolated starting material **15** (15 mg, 0.045 mmol, 15%); ^{1}H NMR (600 MHz, $CDCl_3$) δ 5.01 (t, J = 6.4 Hz, 1H), 4.84 (s, 1H), 4.79 (s, 1H), 4.28 (d, J = 10.2 Hz, 1H), 3.86 (d, J = 10.2 Hz, 1H), 3.41 (d, J = 10.4 Hz, 1H), 3.24 (td, J = 11.2, 6.8 Hz, 1H), 3.15 (dd, J = 17.7, 4.8 Hz, 1H), 2.95 – 2.77 (m, 1H), 2.66 – 2.43 (m, 4H), 2.36 (dd, J = 17.7, 11.3 Hz, 1H), 1.97 (dd, J = 15.6, 6.0 Hz, 1H), 1.78 (s, 3H), 1.50 (s, 3H), 0.90 (t, J = 7.9 Hz, 9H), 0.50 (q, J = 7.9 Hz, 6H). ^{13}C NMR (125 MHz, $CDCl_3$) δ 197.4, 174.1, 146.4, 146.2, 138.1, 110.6, 93.7, 85.1, 83.6, 80.1, 60.8, 44.6, 44.5, 43.3, 42.1, 38.3, 35.8, 22.8, 20.5, 7.1, 6.2; IR (neat film, NaCl) 2955, 1766, 1682, 1372, 1116, 1004, 735, 627 cm^{-1}; HRMS (ESI+): m/z calc'd for $C_{19}H_{21}O_4$ [M–OTES]$^{+}$: 313.1434, found 331.1442; $[\alpha]_D^{23}$ + 10.4 (*c* 0.20, $CHCl_3$).

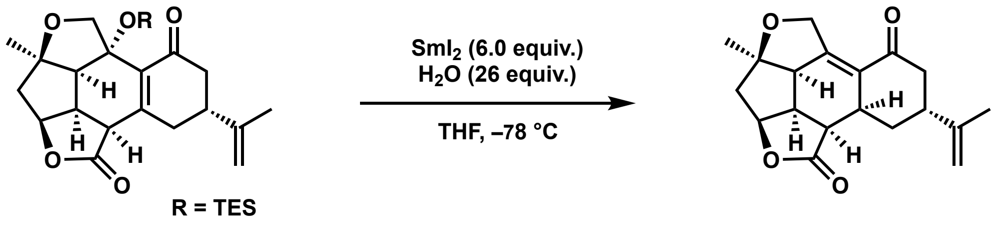

26 **16**

Preparation of Samariumdiiode (0.1 M in THF): Samarium metal (325 mg, 2.16 mmol, 1.7 equiv.) was freshly filed and the resulting fine powder submitted in a Schlenk tube. The tube was set under vacuum and flame-dried, letting it cool back down to 23 °C. THF (125 mL, 0.1 M) was added, followed by EtI_2 (350 mg, 1.24 mmol, 1.0 equiv.). The reaction mixture was stirred for 10 h, becoming dark blue in color, before it was used. The stock solution was kept under N_2 and sealed with a stopcock, storing it for up to two months without observing any loss of reactivity.

Enone 16: A vial was charged with Silylether **26** (19.2 mg, 0.04 mmol, 1.0 equiv.), dissolved in dry THF (0.4 mL, 0.1 M), the reaction mixture cooled to –78 °C with a dry ice/acetone bath and H_2O (17.3 μL, 0.96 mmol, 24.0 equiv.) added dropwise. SmI_2 (0.1 M in THF, 2.6 mL, 6.0 equiv.) was slowly added down the side of the reaction flask and the dark blue reaction mixture stirred for exactly 2 hours at the same temperature. The dry ice/acetone bath was removed and replaced with an ice bath to let the reaction mixture slowly warm up to 0 °C. After one hour, the reaction was quenched with a mixture of a saturated aqueous solution of $Na_2S_2O_3$ and hexanes (4 mL, 1:8). After stirring for 2 minutes, Na_2SO_4 was added and the mixture filtered over a plug of silica gel, eluting with EtOAc. The solvent was evaporated under reduced pressure and the crude product purified by flash chromatography on silica gel (20% EtOAc/hexanes → 30% EtOAc/hexanes) to yield the title compound **16** (8.0 mg, 0.025 mmol, 63%) as a white foam; ^1H NMR (600 MHz, $CDCl_3$) δ 4.96 – 4.89 (m, 2H), 4.85 (td, *J* = 7.6, 3.8 Hz, 1H), 4.82 (s, 1H), 4.51 (s, 1H), 3.38 (td, *J* = 9.7, 7.8 Hz, 1H), 2.99 (dd, *J* = 10.0, 3.9 Hz, 1H), 2.94 (ddd, *J* = 14.3, 10.7, 4.0 Hz, 1H), 2.82 (s, 1H), 2.71 – 2.50 (m, 4H), 2.21 (dd, *J* = 15.6, 7.5 Hz, 1H), 2.14 – 2.07 (m, 1H), 2.01 (dd, *J* = 15.7, 4.0 Hz, 1H), 1.80 (s, 3H), 1.48 (s, 3H); ^{13}C NMR (125 MHz, $CDCl_3$) δ 197.4, 177.2, 156.8, 146.0, 127.5, 111.9, 89.9, 82.6, 71.0, 53.6, 44.2, 43.7, 43.5, 42.2, 38.0, 34.7, 26.5, 25.9, 22.2; IR (neat film, NaCl) 2922, 1761, 1616, 1315, 1110, 950, 798, 637 cm^{-1}; HRMS (ESI+): m/z calc'd for $C_{19}H_{23}O_4$ $[M+H]^+$: 315.1591, found 315.1588; $[\alpha]_D^{23}$ + 10.1 (*c* 0.15, $CHCl_3$).

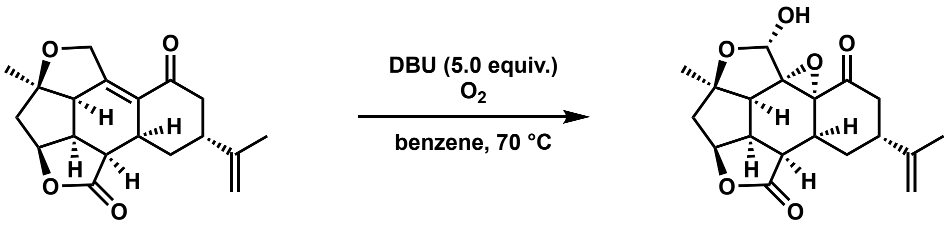

16 **17**

Hemiacetal 17: A vial was charged with starting material **16** (8.0 mg, 0.025 mmol, 1.0 equiv.), dissolved in benzene (2.5 mL, 0.01 M) and DBU (20 µL, 0.12 mmol, 5.0 equiv.) added dropwise. The head space of the vial was briefly flushed with a blow of oxygen (1 second) and sealed shut with a plastic cap. The vial was placed in a preheated heating block set to 70 °C and stirred for 3 h until full consumption of starting material was observed as determined by TLC. The reaction mixture was directly purified by flash chromatography on silica gel (hexanes → EtOAc/hexanes/acetone, 4:5:1) to yield the title compound **17** (5.8 mg, 0.016 mmol, 67%) as a white foam. ***Note:*** *The reaction was very sensitive to the speed of stirring; being kept low (around 200 to 300 rpm), as a loss in yield was observed at higher rates*; ^{1}H NMR (400 MHz, $CDCl_3$) δ 5.60 (s, 1H), 4.98 (s, 1H), 4.87 (t, *J* = 5.0 Hz, 1H), 4.71 (s, 1H), 3.19 (ddd, *J* = 10.8, 8.1, 5.0 Hz, 1H), 3.07 (dd, *J* = 17.2, 5.0 Hz, 1H), 2.99 (dt, *J* = 6.3, 3.2 Hz, 2H), 2.86 – 2.75 (m, 1H), 2.74 – 2.62 (m, 2H), 2.54 (d, *J* = 10.8 Hz, 1H), 2.37 (dddd, *J* = 14.0, 9.6, 4.3, 2.6 Hz, 1H), 2.13 – 2.02 (m, 1H), 1.87 (dd, *J* = 16.1, 5.0 Hz, 1H), 1.81 (s, 3H), 1.71 (s, 3H); ^{13}C NMR (100 MHz, $CDCl_3$) δ 205.6, 176.6, 145.3, 112.7, 95.2, 93.7, 85.1, 74.4, 60.6, 50.4, 45.6, 45.4, 44.9, 44.5, 38.2, 35.5, 29.2, 28.6, 22.1; IR (neat film, NaCl) 3335, 2929, 1756, 1600, 1124, 1007, 735, 624 cm^{-1}; HRMS (ESI+): m/z calc'd for $C_{19}H_{21}O_5$ $[M+H]^+$: 329.1384, found 329.1383; $[\alpha]_D^{23}$ + 7.5 (*c* 0.23, $CHCl_3$).

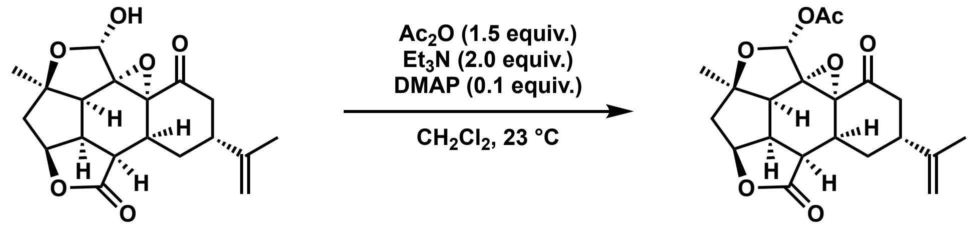

17 ***18***

Acetal 18: A vial was charged with **17** (26.3 mg, 0.076 mmol, 1.0 equiv.) and dissolved in CH_2Cl_2 (0.76 mL, 0.1 M). Et_3N (21.2 μL, 0.15 mmol, 2.0 equiv.) and Acetic anhydride (10.7 μL, 0.11 mmol, 1.5 equiv.) were added dropwise. Subsequently, DMAP (0.9 mg, 0.0076 mmol, 0.1 equiv.) was added and the reaction stirred at 23 °C for 1.5 h. The crude reaction mixture was directly purified by flash chromatography on silica gel (hexanes → EtOAc/hexanes/acetone, 4:5:1) and the title compound **18** (26.8 mg, 0.065 mmol, 85% yield) isolated as a white foam; ^{1}H NMR (600 MHz, $CDCl_3$) δ 6.56 (s, 1H), 4.97 (s, 1H), 4.87 (t, *J* = 5.0 Hz, 1H), 4.71 (s, 1H), 3.20 (ddd, *J* = 11.9, 8.0, 5.1 Hz, 1H), 3.06 (dd, *J* = 17.4, 5.1 Hz, 1H), 3.00 (dd, *J* = 8.3, 2.6 Hz, 1H), 2.99 – 2.95 (m, 1H), 2.79 (dt, *J* = 17.7, 3.1 Hz, 1H), 2.74 – 2.65 (m, 2H), 2.57 (d, *J* = 10.8 Hz, 1H), 2.43 – 2.32 (m, 1H), 2.11 (s, 3H), 2.10 – 2.04 (m, 1H), 1.87 (dd, *J* = 16.2, 5.0 Hz, 1H), 1.80 (s, 3H), 1.64 (s, 3H); ^{13}C NMR (100 MHz, $CDCl_3$) δ 204.4, 176.3, 169.6, 145.3, 112.6, 94.6, 93.8, 84.9, 72.0, 59.9, 50.4, 45.3, 45.1, 44.8, 44.6, 38.1, 35.6, 28.6, 28.5, 22.0, 21.4; IR (neat film, NaCl) 2929, 1748, 1715, 1362, 1228, 1131, 1080, 1005, 940, 736 cm^{-1}; HRMS (ESI+): m/z calc'd for $C_{21}H_{24}O_7Na$ [M+Na]$^+$: 411.1414, found 411.1412; $[\alpha]_D^{23}$ + 18.6 (*c* 0.12, $CHCl_3$).

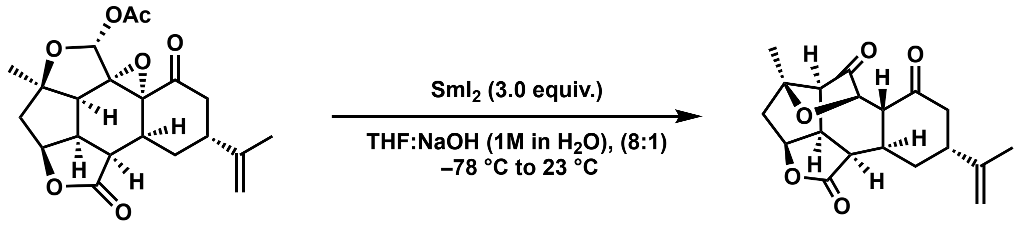

18 ***(+)-ineleganolide (1)***

(+)-ineleganolide (1): A vial was charged with **18** (11.0 mg, 0.026 mmol, 1.0 equiv.), dissolved in THF (1.07 mL, 0.025 M) and the reaction mixture cooled to –78 °C in a dry ice/acetone bath. Aqueous NaOH (1M, 133 μL) was added dropwise, before SmI_2 (0.1 M in THF, 801 μL, 0.08 mmol, 3.0 equiv.) was added dropwise. The reaction mixture was stirred for 3 minutes, before the dry ice/acetone bath was removed. The reaction mixture was stirred for a total of another 3 minutes, while warming up to 23 °C, before it was quenched with a mixture of a saturated aqueous solution of $Na_2S_2O_3$ and hexanes (2 mL, 1:8). After stirring for 2 minutes, Na_2SO_4 was added and the mixture filtered over a plug of silica gel, eluting with EtOAc. The solvent was evaporated under reduced pressure and the crude product purified by preparative HPLC (Agilent Zorbax RX-SIL 5μm (SiO_2), Hexanes:CH_2Cl_2 (3:1) / iPrOH (10% → 25% over 7.5 min), flowrate: 7 mL/min, monitor wavelength = 206 nm, retention time = 3.9 min) to yield (+)-ineleganolide (**1**, 4.0 mg, 0.012 mmol, 45%) as a white solid; ^{1}H NMR (400 MHz, $CDCl_3$) δ 5.12 (t, *J* = 7.4 Hz, 1H), 5.07 (s, 1H), 4.94 (s, 1H), 4.62 (s, 1H), 3.42 (ddd, *J* = 12.1, 9.2, 7.6 Hz, 1H), 3.02 (dd, *J* = 12.0, 2.4 Hz, 1H), 3.00 (m, 1H), 2.78 (br s, 1H), 2.70 (d, *J* = 13.0 Hz, 1H), 2.64 (m, 1H), 2.59 (d, *J* = 9.3 Hz, 1H), 2.52 (d, *J* = 15.6 Hz, 1H), 2.25 (tt, *J* = 12.4, 2.8 Hz, 1H), 2.10 (dd, *J* = 15.5, 7.3 Hz, 1H), 1.78 (dq, *J* = 13.0, 2.8 Hz, 1H), 1.71 (s, 2H), 1.28 (s, 3H). ^{13}C NMR (100 MHz, $CDCl_3$) δ 212.2, 206.4, 176.1, 146.0, 113.8, 91.1, 83.1, 77.4, 62.5, 49.8, 47.0, 45.5, 44.4, 43.8, 40.4, 33.2, 32.7, 22.7, 20.2; IR (neat film, NaCl) 2935, 1759, 1705, 1372, 1322, 1213, 1184, 1026, 905, 835, 736 cm^{-1}; HRMS (ESI+): m/z calc'd for $C_{19}H_{23}O_5$ $[M+H]^+$: 331.1540, found 331.1538; $[\alpha]_D^{23}$ + 25.1 (*c* 0.22, $CHCl_3$).

Comparison of Natural and Synthetic Ineleganolide

Table S1: Comparison of natural, reported and synthetic ineleganolide ^{1}H NMR data.

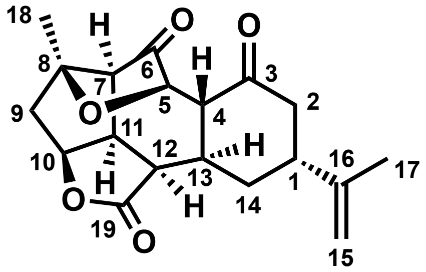

Position	Natural δ ^{1}H (400 MHz) (Ref. 5)	Reported δ ^{1}H (400 MHz) (Ref. 6)	Synthetic δ ^{1}H (400 MHz)
1	2.78 (br s)	2.79 (br s)	2.78 (br s)
2	2.63 (m)	2.64 (m)	2.64 (m)
4	2.70 (d, *J* = 13.0 Hz)	2.70 (d, *J* = 13.1 Hz)	2.70 (d, *J* = 13.0 Hz)
5	5.07 (s)	5.07 (s)	5.07 (s)
7	2.59 (d, *J* = 9.3 Hz)	2.59 (d, *J* = 9.3 Hz)	2.59 (d, *J* = 9.3 Hz)
9α	2.10 (dd, *J* = 15.6, 7.2 Hz)	2.10 (dd, *J* = 15.7, 7.3 Hz)	2.10 (dd, *J* = 15.5, 7.3 Hz)
9β	2.51 (d, *J* = 15.6 Hz)	2.52 (d, *J* = 15.5 Hz)	2.52 (d, *J* = 15.6 Hz)
10	5.13 (t, *J* = 7.2 Hz)	5.12 (t, *J* = 7.3 Hz)	5.12 (t, *J* = 7.4 Hz)
11	3.42 (ddd, *J* = 12.3, 9.3, 7.2 Hz)	3.42 (ddd, *J* = 12.2, 9.3, 7.5 Hz)	3.42 (ddd, *J* = 12.1, 9.2, 7.6 Hz)
12	3.02 (dd, *J* = 12.3, 2.5 Hz)	3.02 (dd, *J* = 12.2, 2.5 Hz)	3.02 (dd, *J* = 12.0, 2.4 Hz)
13	2.24 (tt, *J* = 13.0, 2.5 Hz)	2.25 (tt, *J* = 12.6, 2.7 Hz)	2.25 (tt, *J* = 12.4, 2.8 Hz)
14α	3.00 (m)	3.00 (m)	3.00 (m)
14β	1.79 (m)	1.79 (dq, *J* = 13.0, 2.6 Hz)	1.78 (dq, *J* = 13.0, 2.8 Hz)
16	1.71 (s)	1.71 (s)	1.71 (s)
17	4.62 (s)	4.62 (s)	4.62 (s)
18	4.94 (s)	4.94 (s)	4.94 (s)
19	1.28 (s)	1.28 (s)	1.28 (s)

Table S2: Comparison of natural, reported and synthetic ineleganolide ^{13}C NMR data.

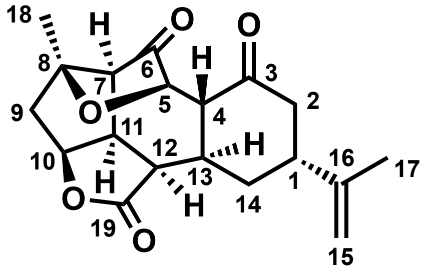

Position	Natural δ ^{13}C (100.6 MHz) (Ref. 5)	Reported δ ^{1}H (100 MHz) (Ref. 6)	Synthetic δ ^{1}H (100 MHz)
1	40.2	40.3	40.4
2	44.3	44.3	44.4
3	206.2	206.3	206.4
4	49.7	49.7	49.8
5	77.4	77.3	77.4
6	211.9	212.1	212.2
7	62.4	62.4	62.5
8	90.9	91.0	91.1
9	45.4	45.4	45.5
10	83.0	83.0	83.1
11	43.7	43.6	43.8
12	46.9	46.9	47.0
13	33.1	33.1	33.2
14	32.6	32.6	32.7
15	145.8	145.9	146.0
16	22.5	22.5	22.7
17	113.6	113.7	113.8
18	20.1	20.1	20.2
19	175.8	175.9	176.1

Figure S1: Overlayed spectra of synthetic and reported ^{1}H NMR spectra of **1** (400 MHz).

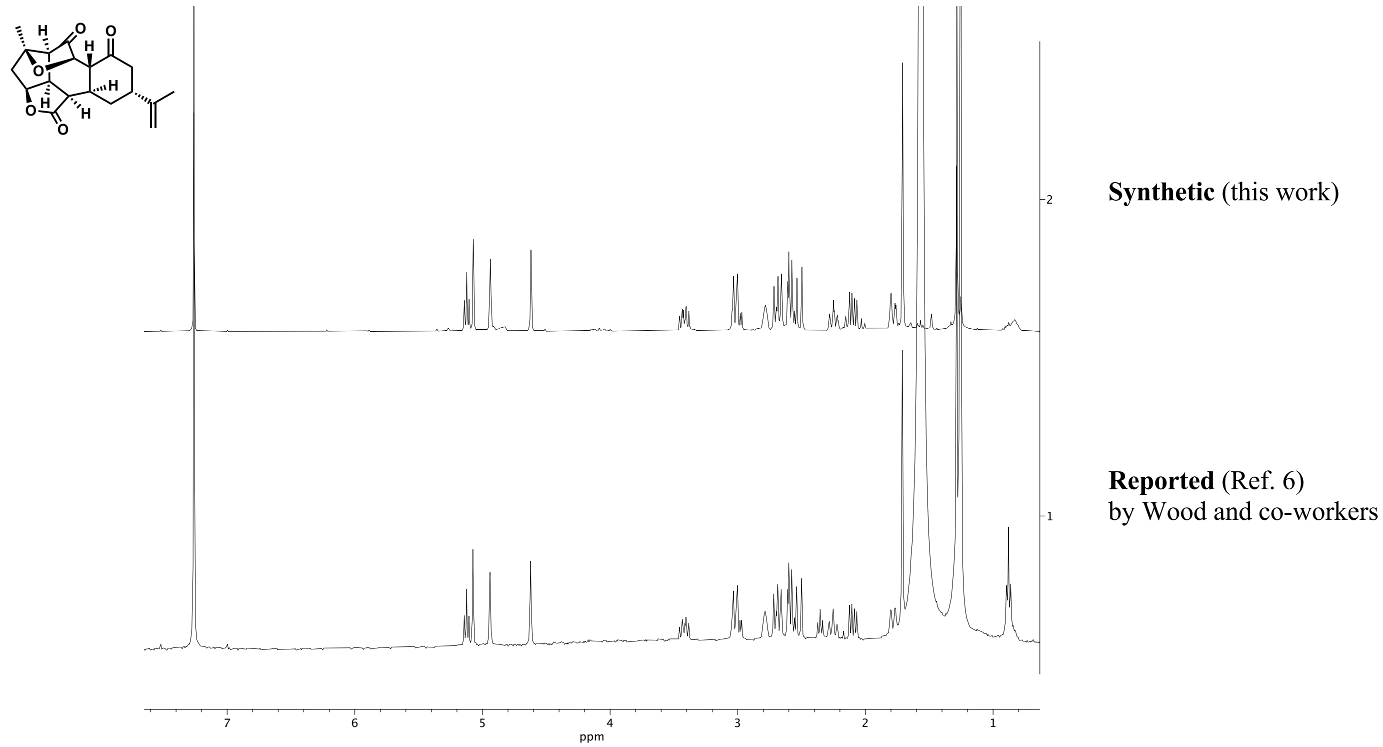

Figure S2: Overlayed spectra of synthetic and reported ^{13}C NMR spectra of **1** (100 MHz).

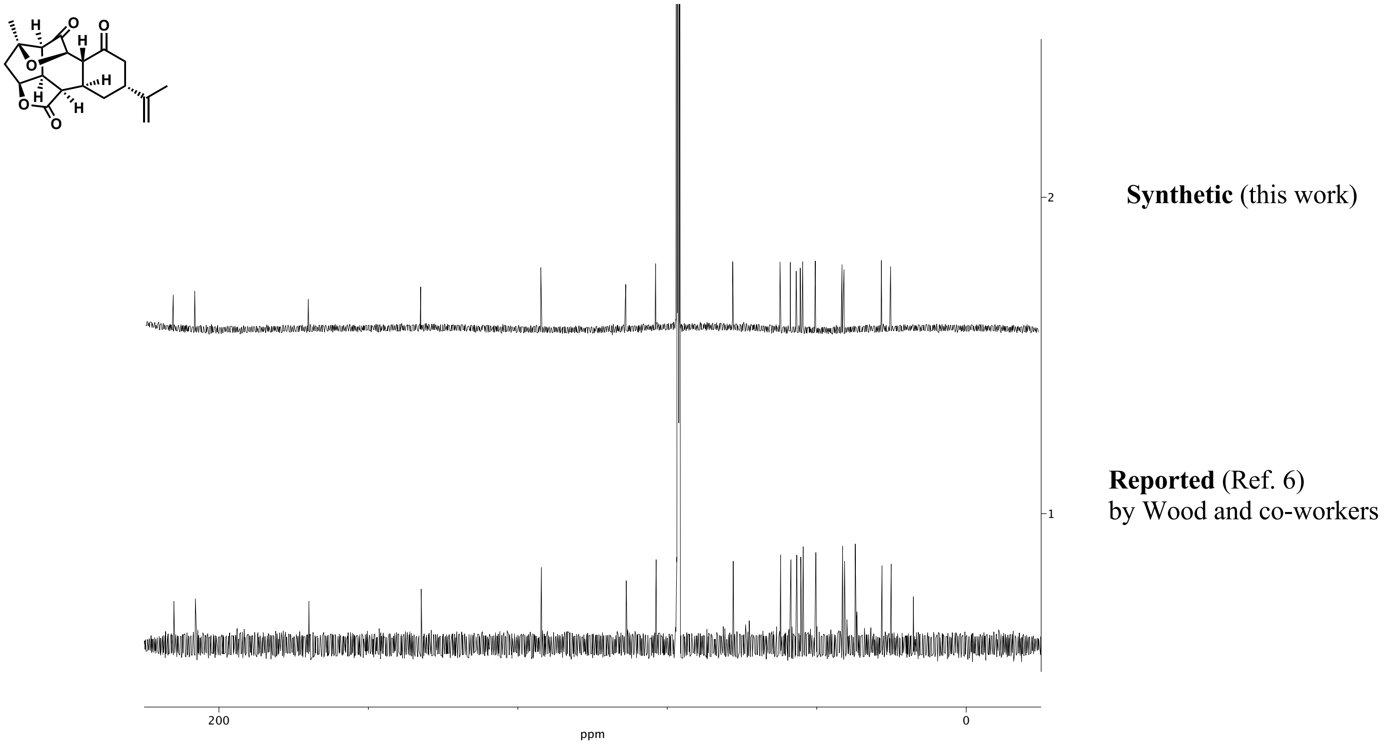

References

1) Pangborn, A. M.; Giardello, M. A.; Grubbs, R. H.; Rosen, R. K.; Timmers, F. J. Safe and Convenient Procedure for Solvent Purification. *Organometallics* **1996**, *15*, 1518–1520.
2) Brill, Z. G.; Grover, H. K.; Maimone, T. J. Enantioselective Synthesis of an Ophiobolin Sesterterpene via a Programmed Radical Cascade. *Science* **2016**, *352*, 1078−1082.
3) Hafeman, N. J.; Chan, M.; Fulton, T. J.; Alexy, E. J.; Loskot, S. A.; Virgil, S. C.; Stoltz, B. M. Asymmetric Total Synthesis of Havellockate. *J. Am. Chem. Soc.* **2022**, *144*, 20232−20236.
4) Craig, R. A.; Roizen, J. L.; Smith, R. C.; Jones, A. C.; Virgil, S. C.; Stoltz, B. M. Enantioselective, convergent synthesis of the ineleganolide core by a tandem annulation cascade. *Chem. Sci.* **2017**, *8*, 507−514.
5) Duh, C. Y.; Wang, S. K.; Chia, M. C.; Chiang, M. Y. A Novel Cytotoxic Norditerpenoid from the Formosan Soft Coral *Sinularia Inelegans*, *Tetrahedron Lett*. **1999**, *40*, 6033–6035.
6) Tuccinardi, J. P.; Wood J. L. Total Syntheses of (+)-Ineleganolide and (−)-Sinulochmodin C. *J. Am. Chem. Soc.* **2022**, *144*, 20539−20547.

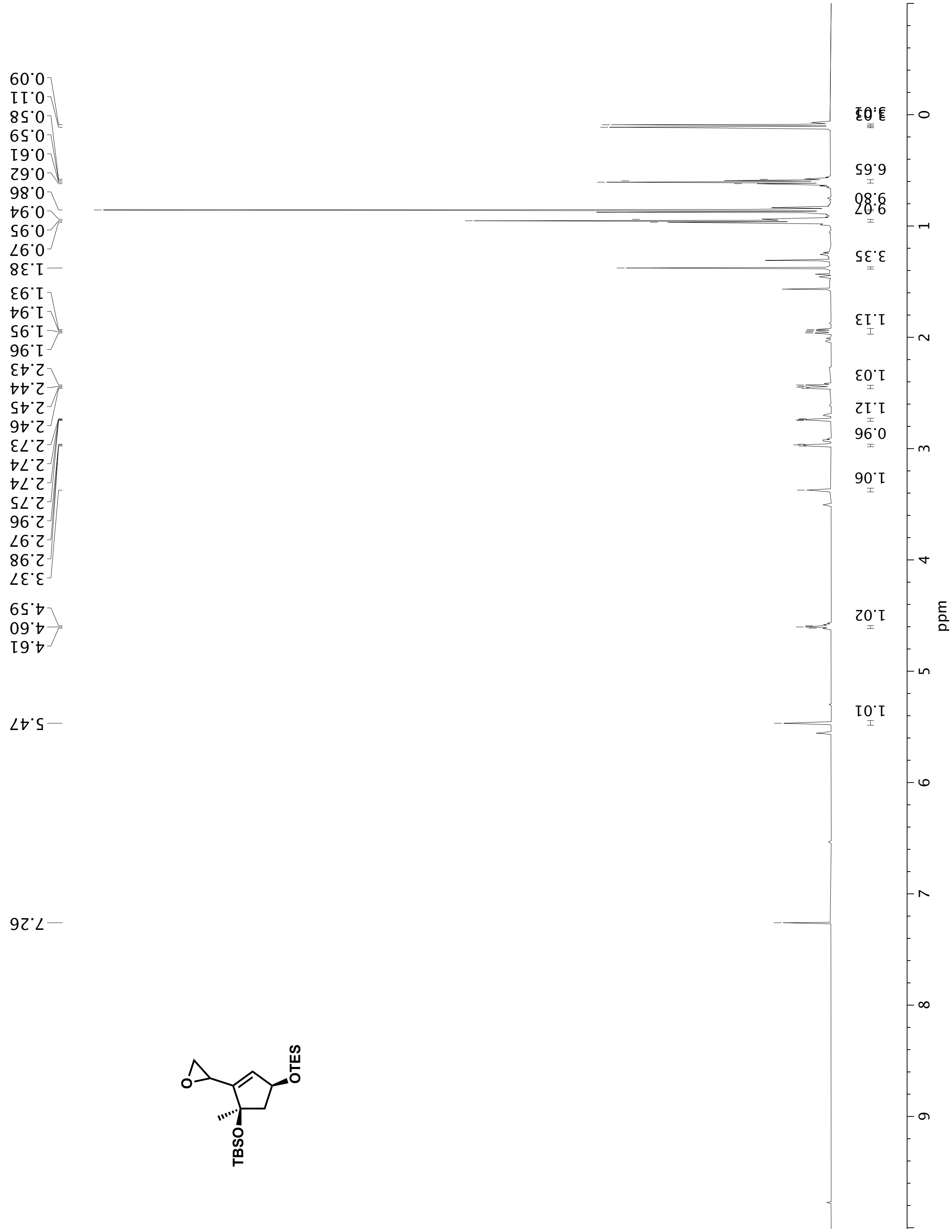

^{1}H NMR (600 MHz, $CDCl_3$) of compound **8**.

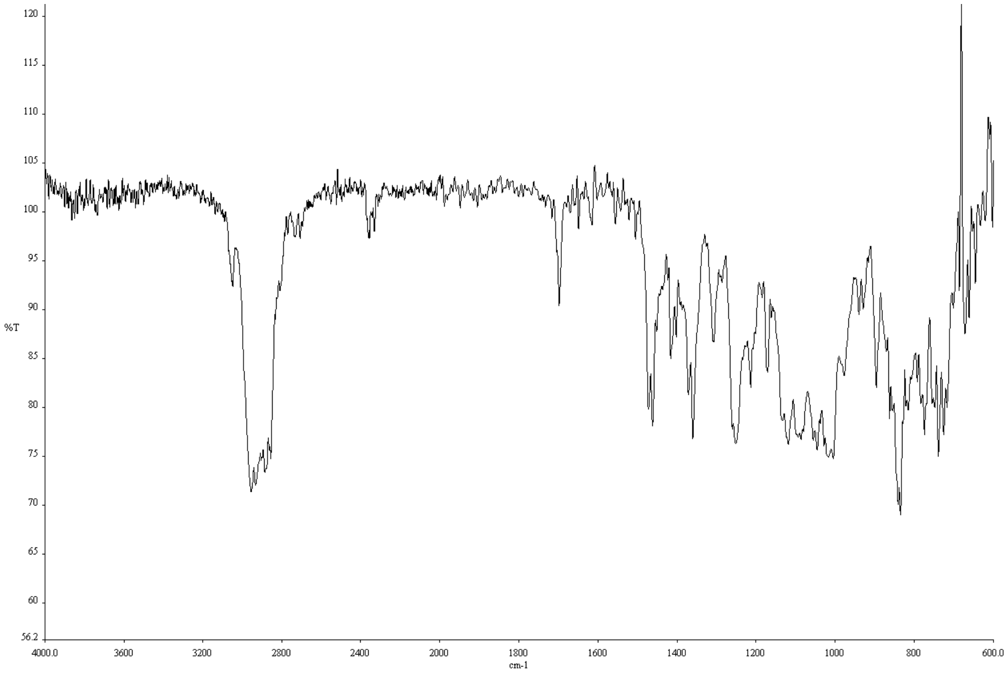

Infrared spectrum (Thin Film, NaCl) of compound **8**.

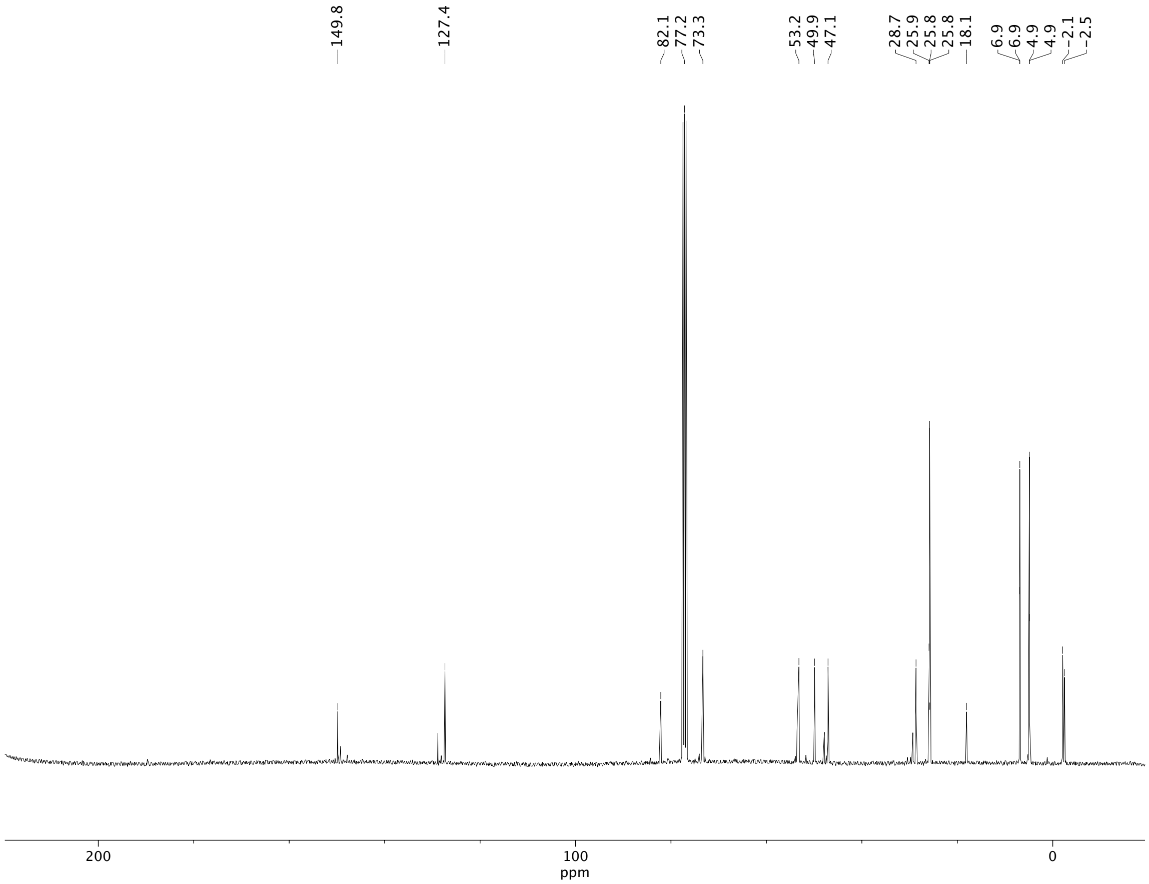

^{13}C NMR (100 MHz, $CDCl_3$) of compound **8**.

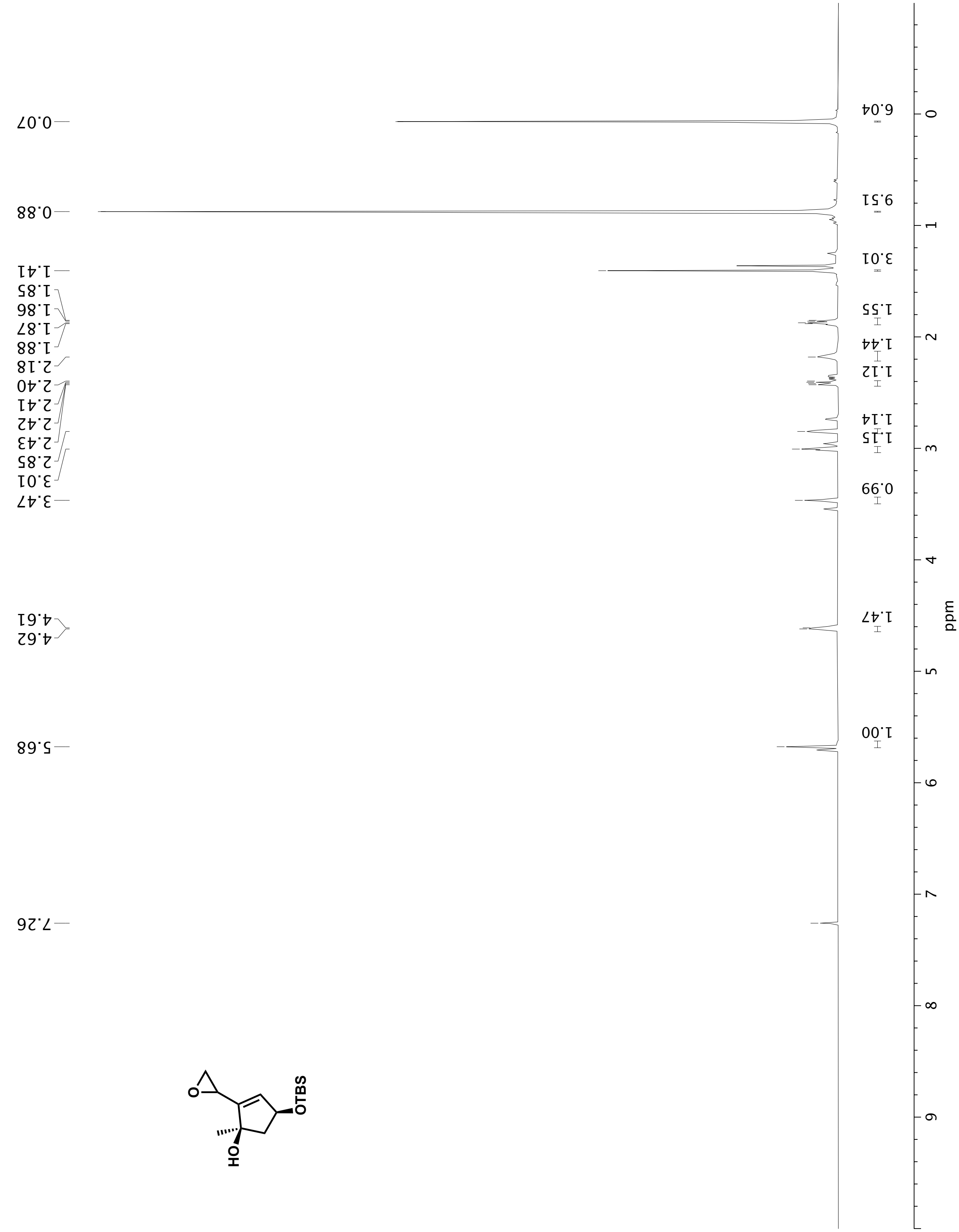

^{1}H NMR (600 MHz, $CDCl_3$) of compound **9**.

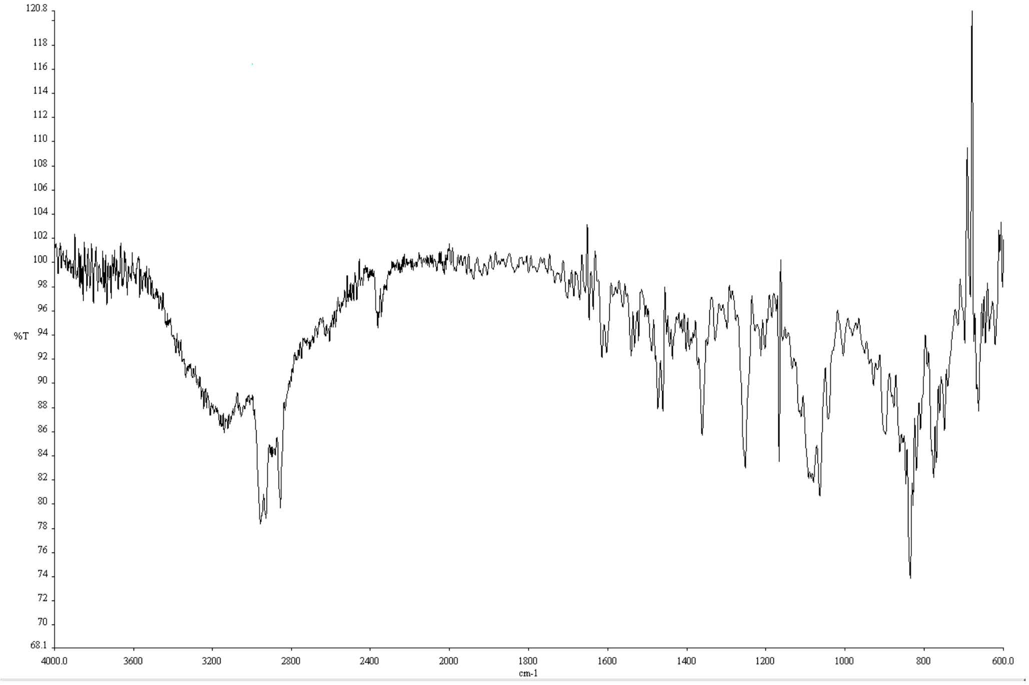

Infrared spectrum (Thin Film, NaCl) of compound **9**.

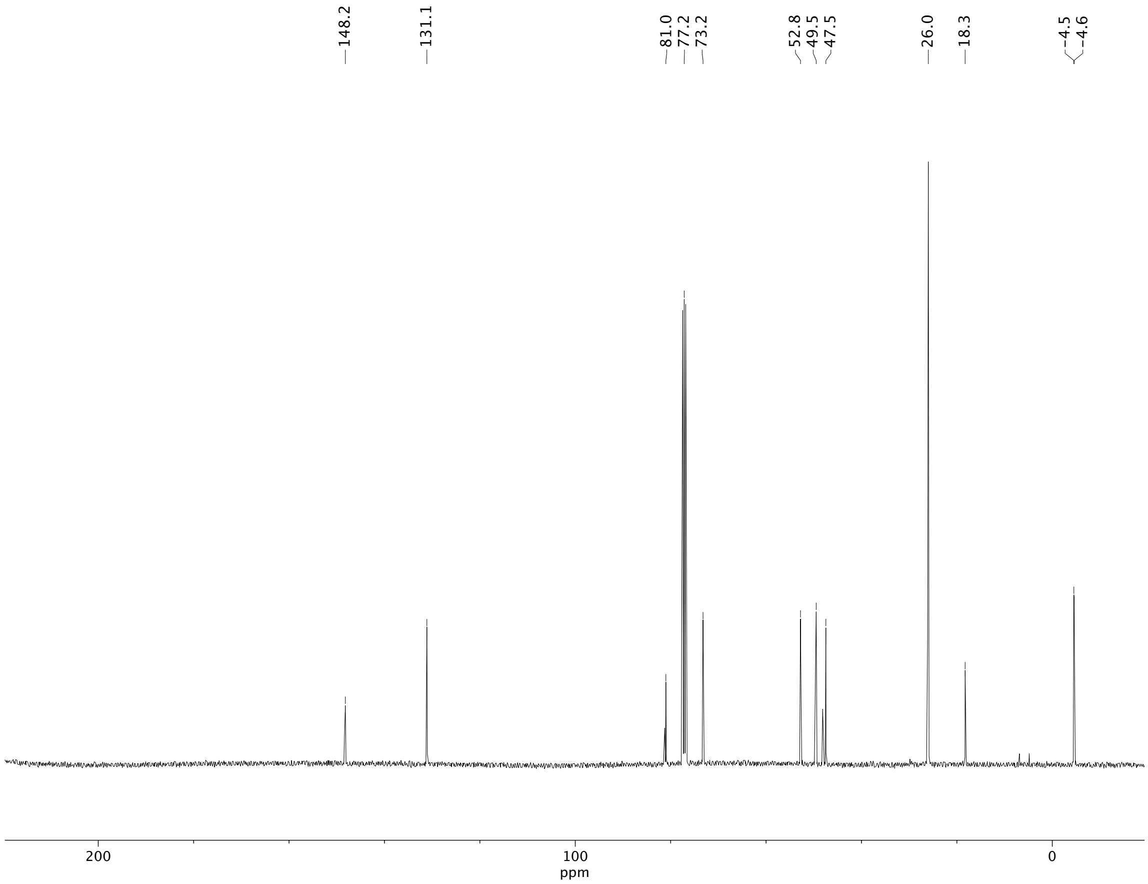

^{13}C NMR (100 MHz, $CDCl_3$) of compound **9**.

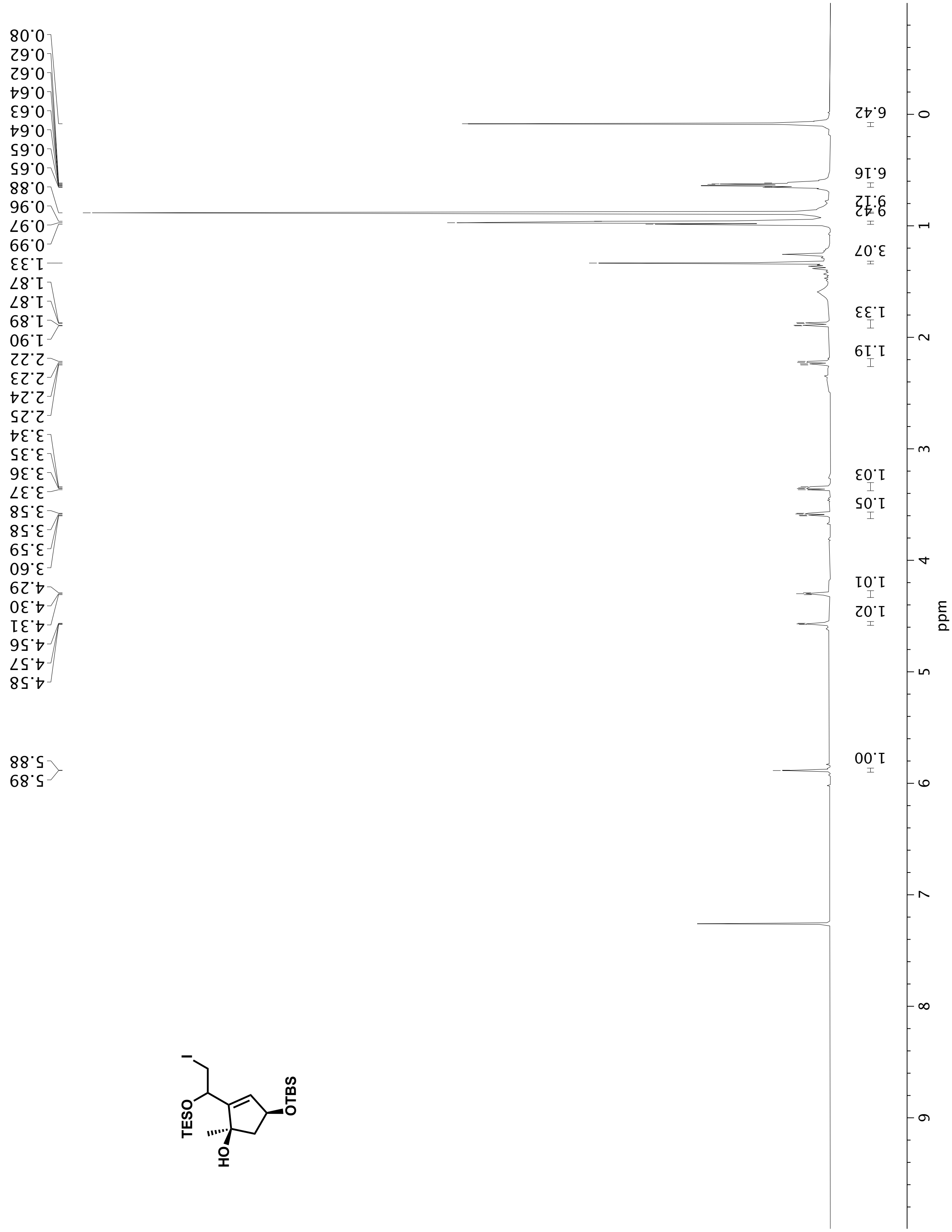

^{1}H NMR (600 MHz, $CDCl_3$) of compound **24**.

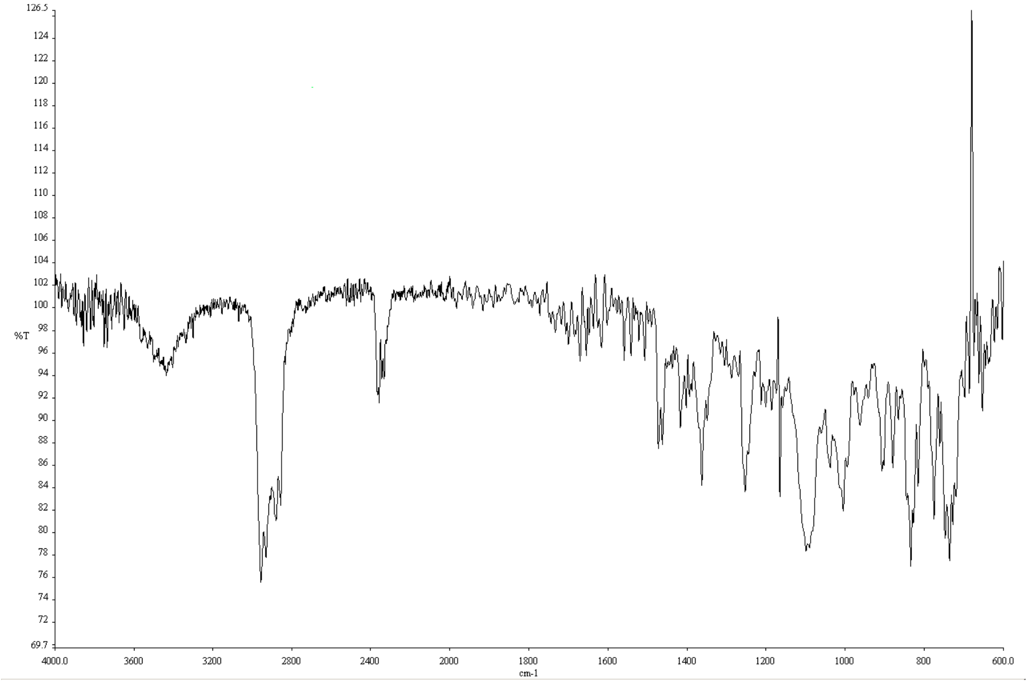

Infrared spectrum (Thin Film, NaCl) of compound **24**.

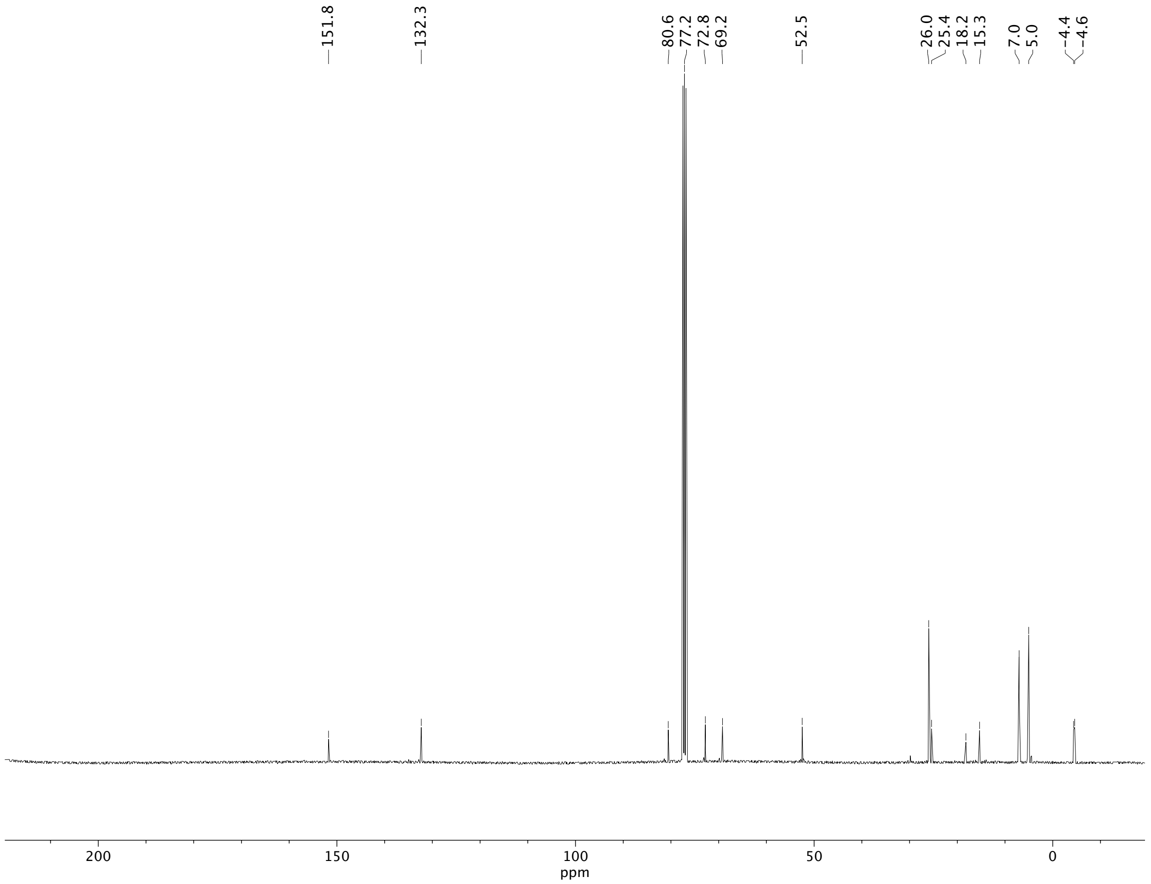

^{13}C NMR (100 MHz, $CDCl_3$) of compound **24**.

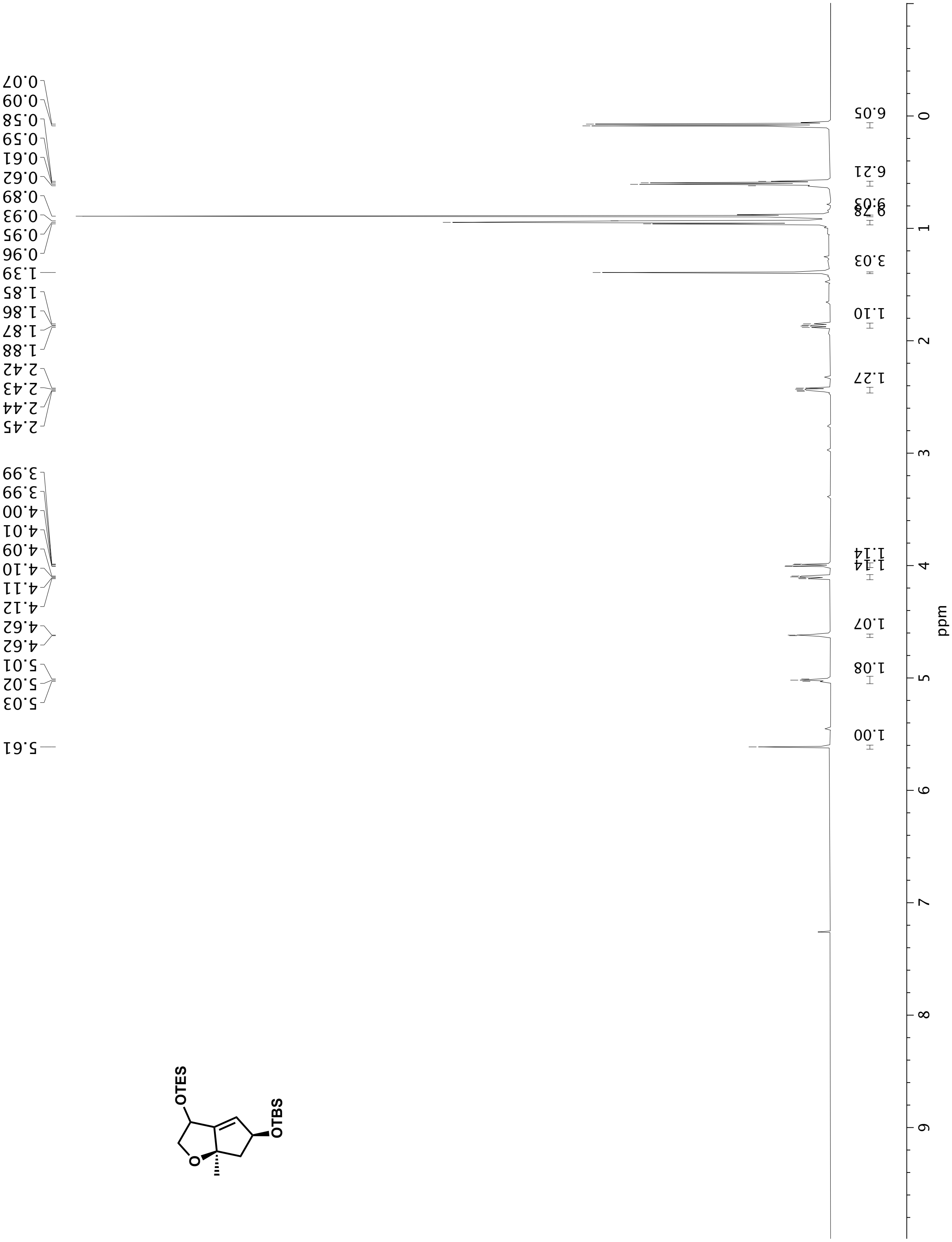

^{1}H NMR (600 MHz, $CDCl_3$) of compound **11**.

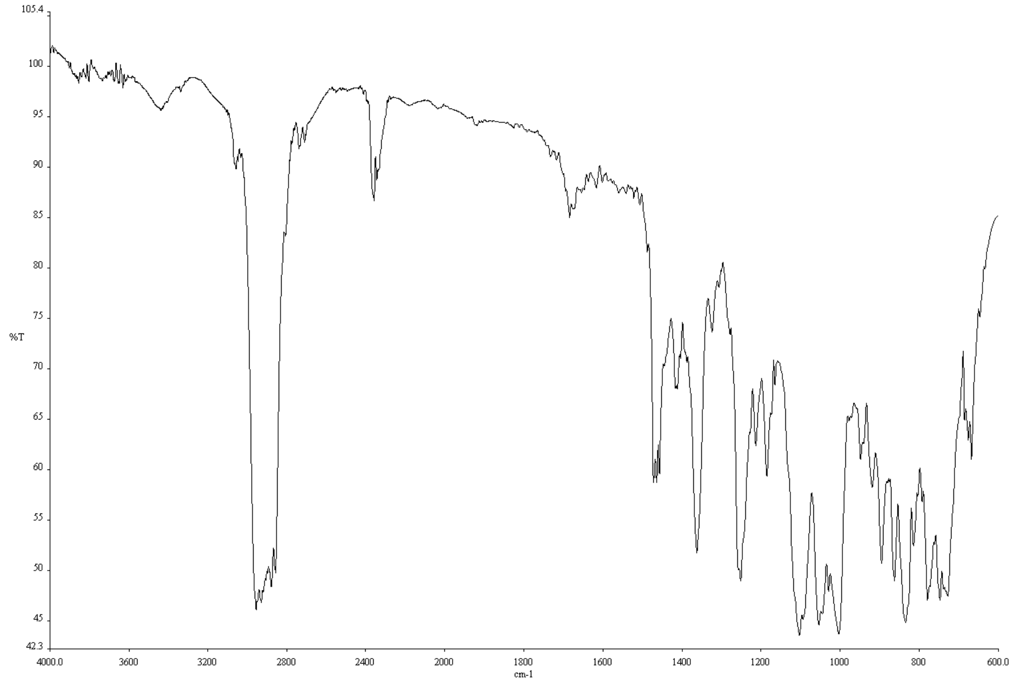

Infrared spectrum (Thin Film, NaCl) of compound **11**.

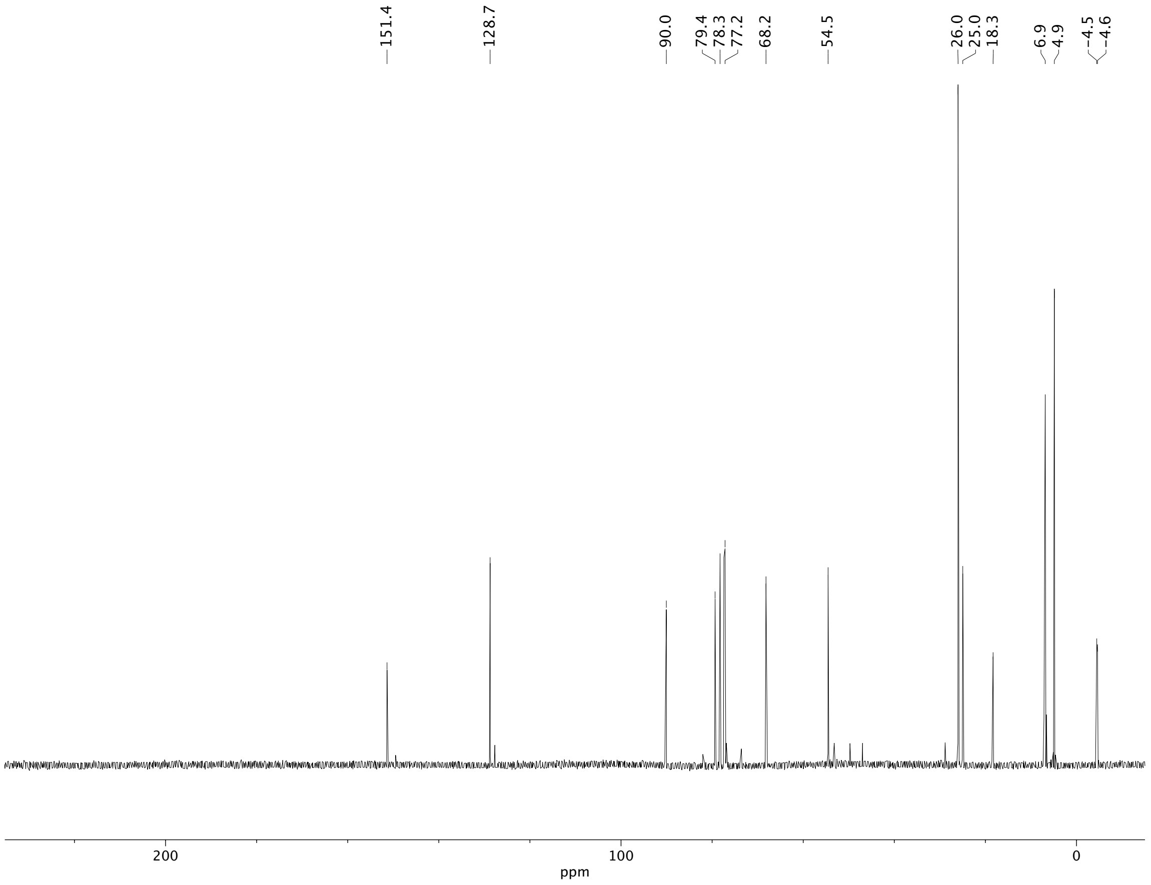

^{13}C NMR (125 MHz, $CDCl_3$) of compound **11**.

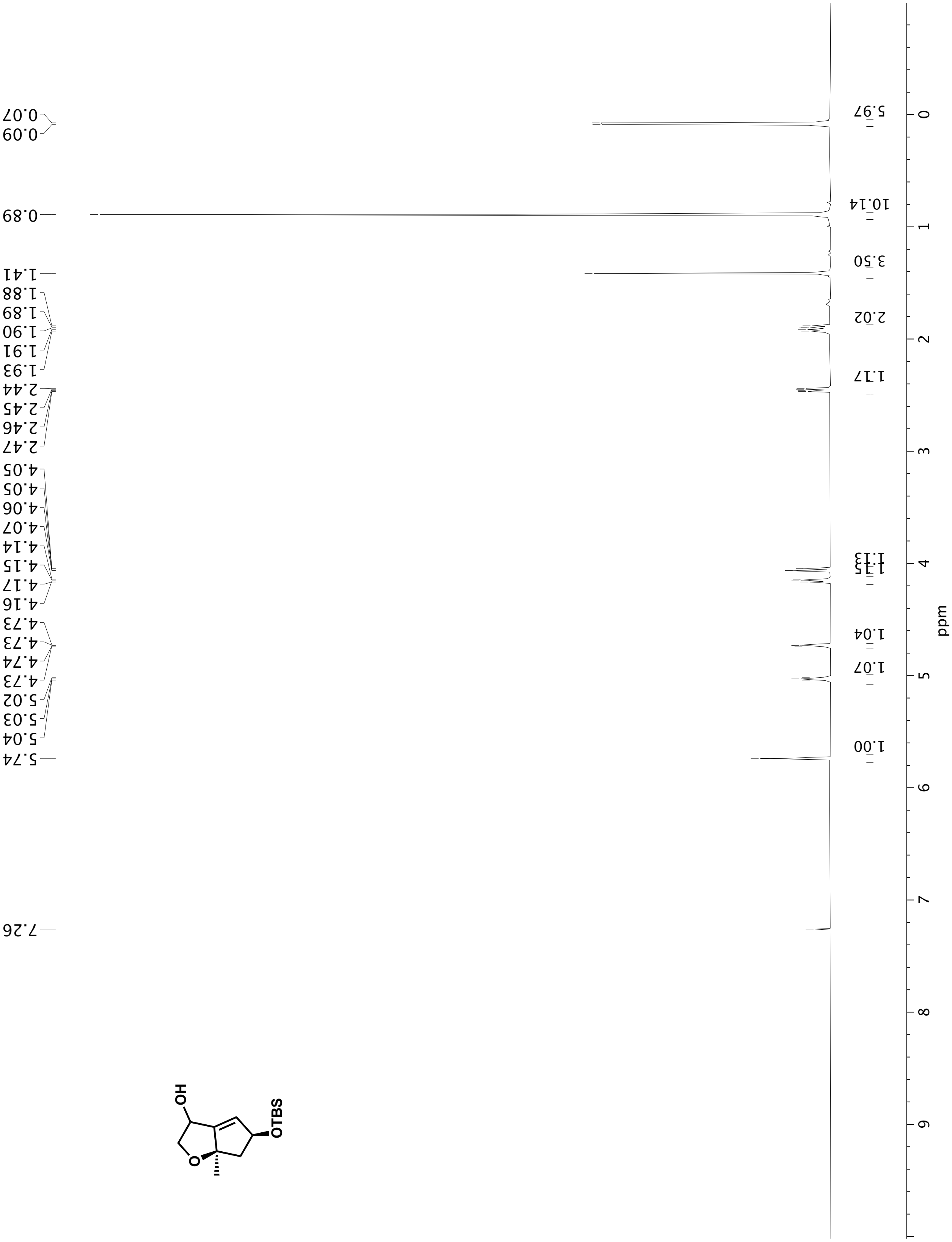

^{1}H NMR (600 MHz, CDCl$_3$) of compound **12**.

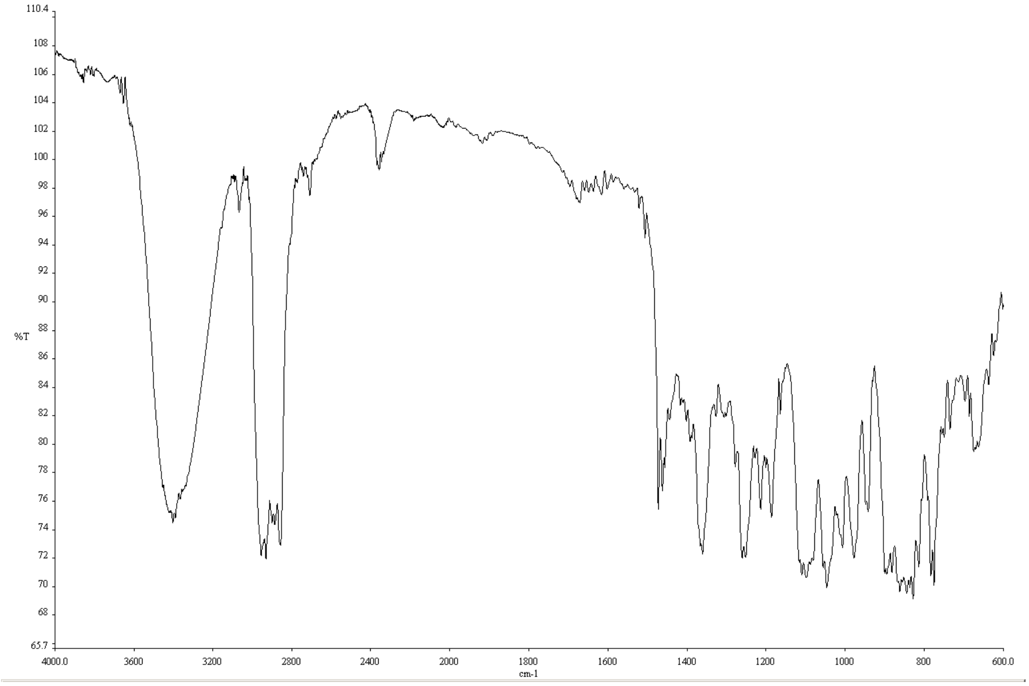

Infrared spectrum (Thin Film, NaCl) of compound **12**.

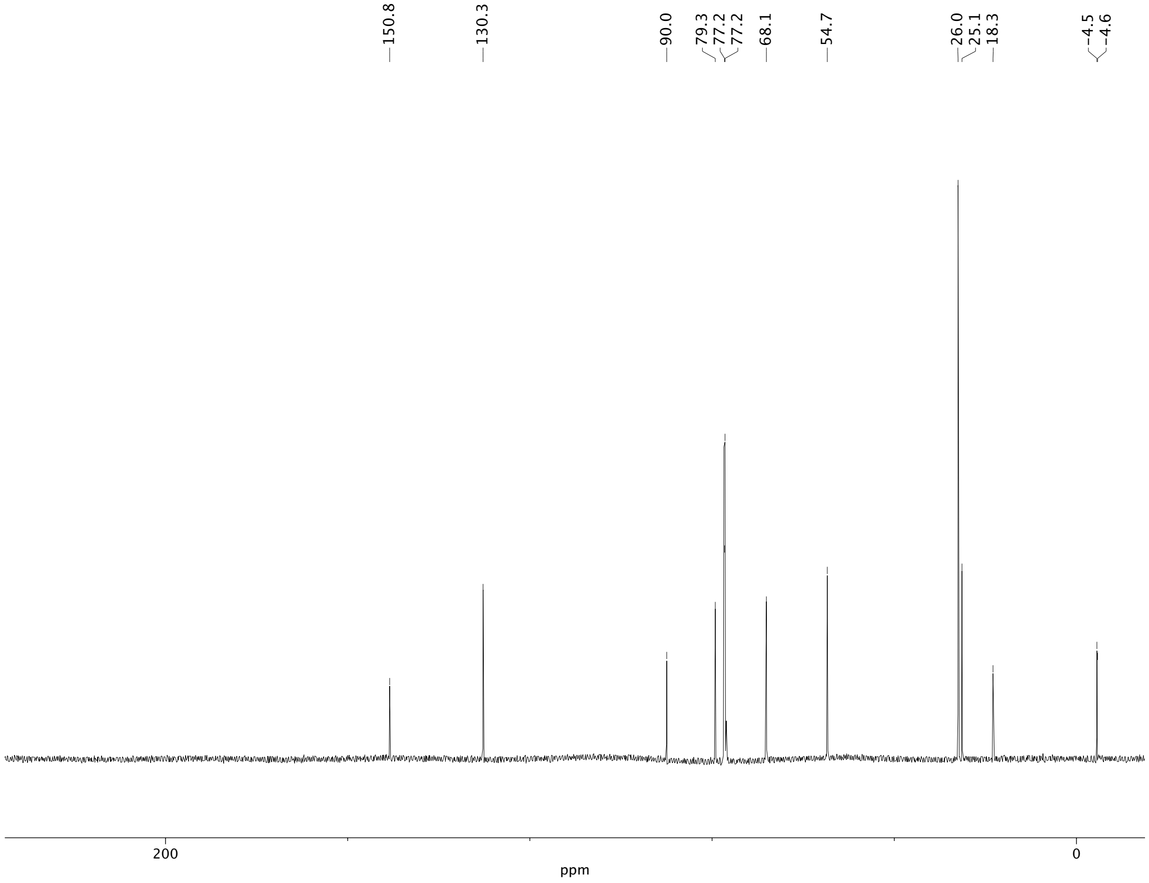

^{13}C NMR (125 MHz, $CDCl_3$) of compound **12**.

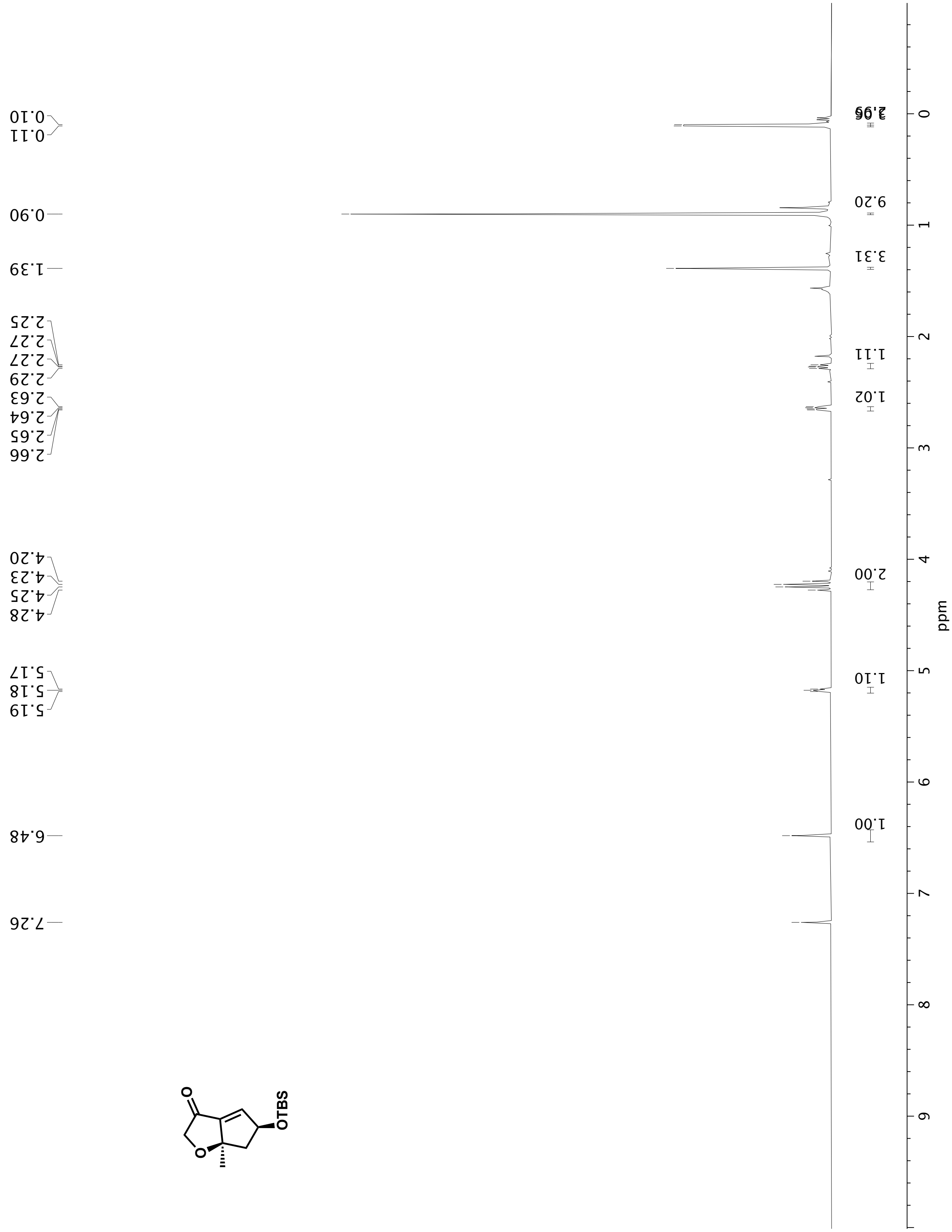

^{1}H NMR (600 MHz, $CDCl_3$) of compound **25**.

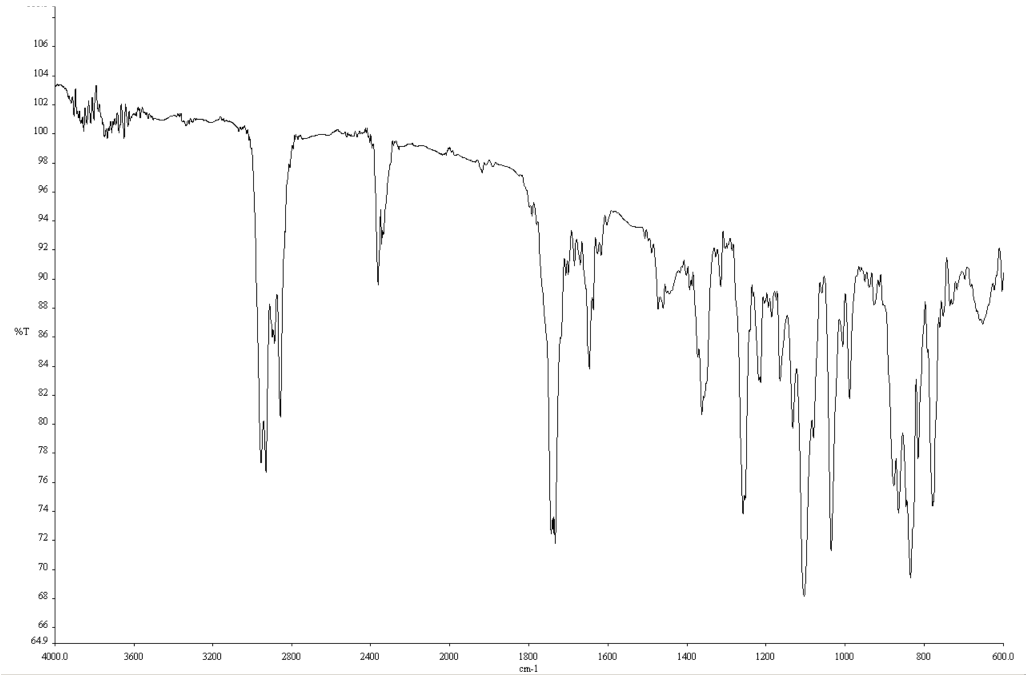

Infrared spectrum (Thin Film, NaCl) of compound **25**.

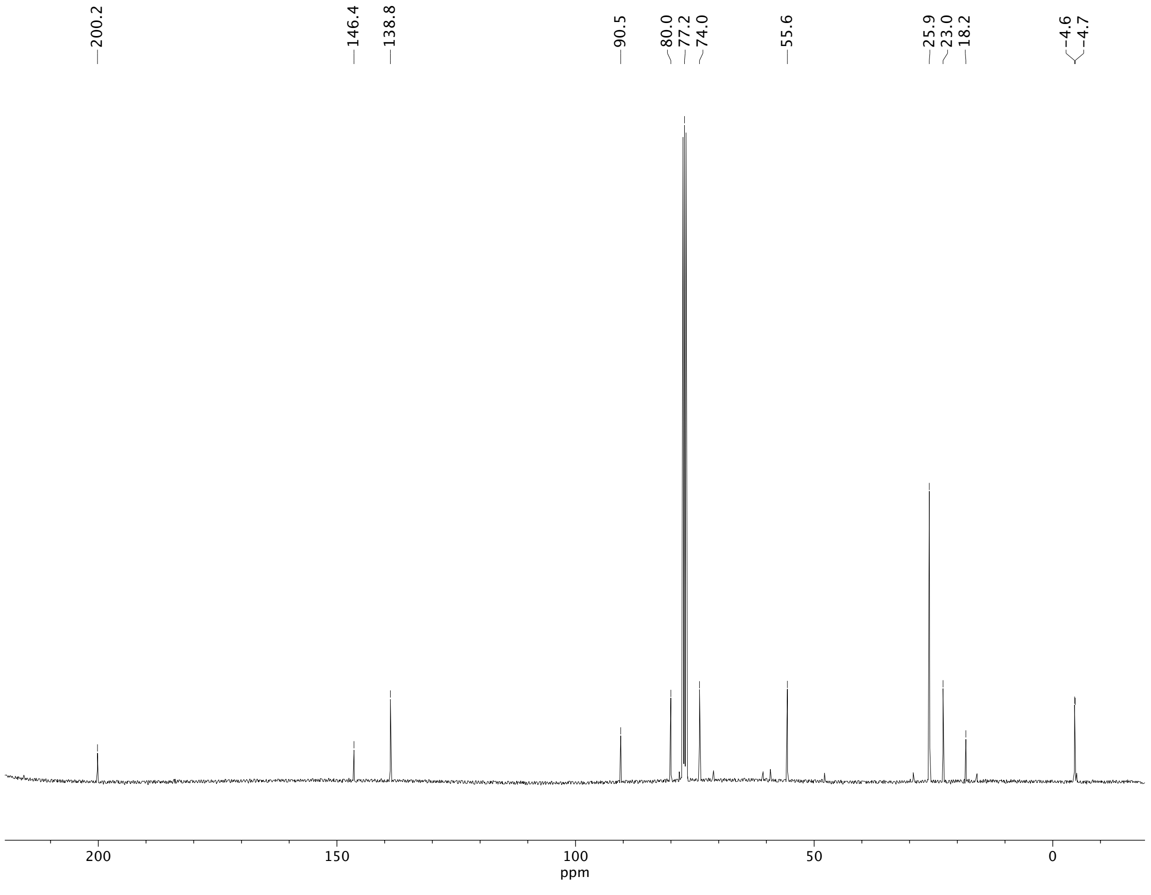

^{13}C NMR (100 MHz, $CDCl_3$) of compound **25**.

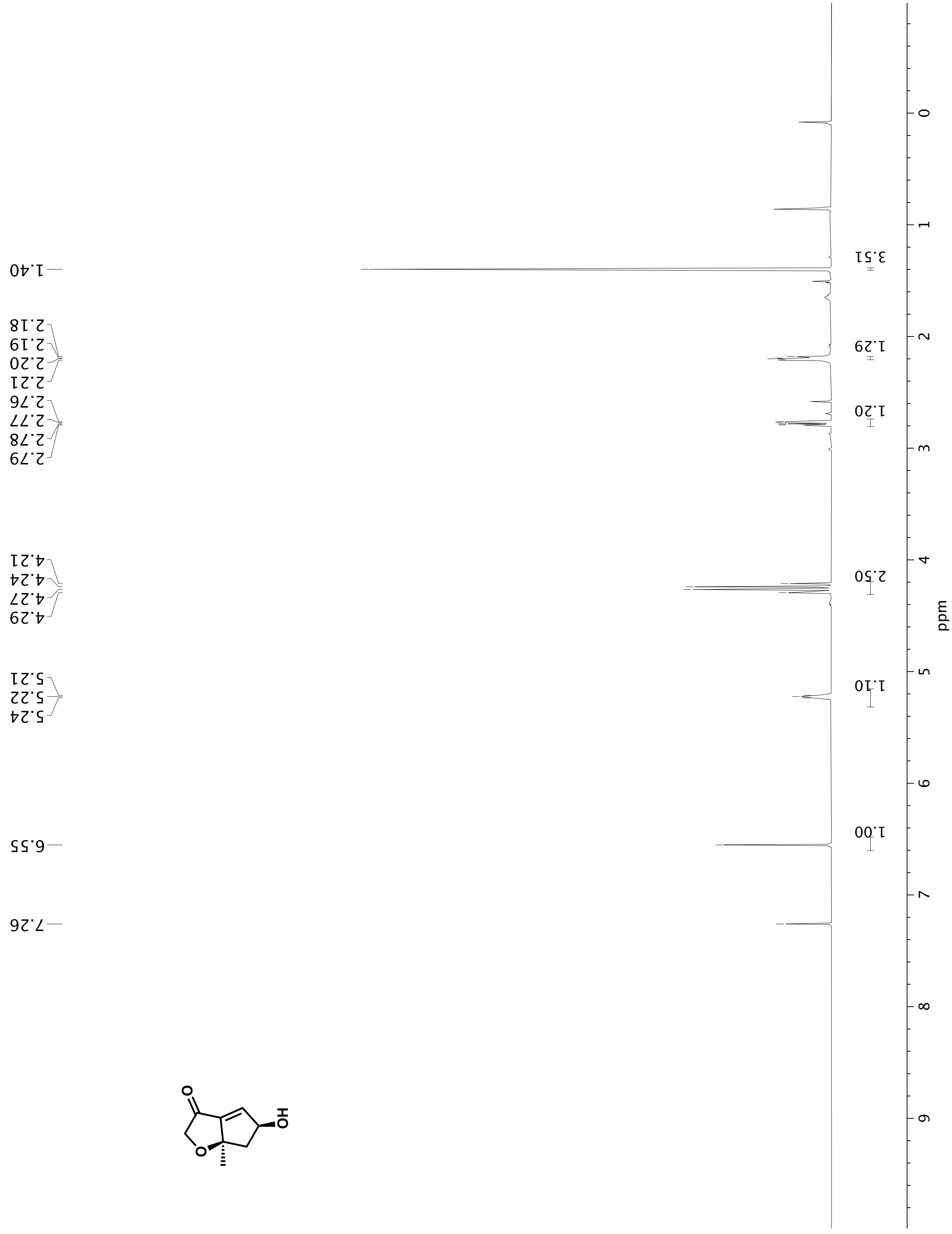

^{1}H NMR (600 MHz, $CDCl_3$) of compound **5**.

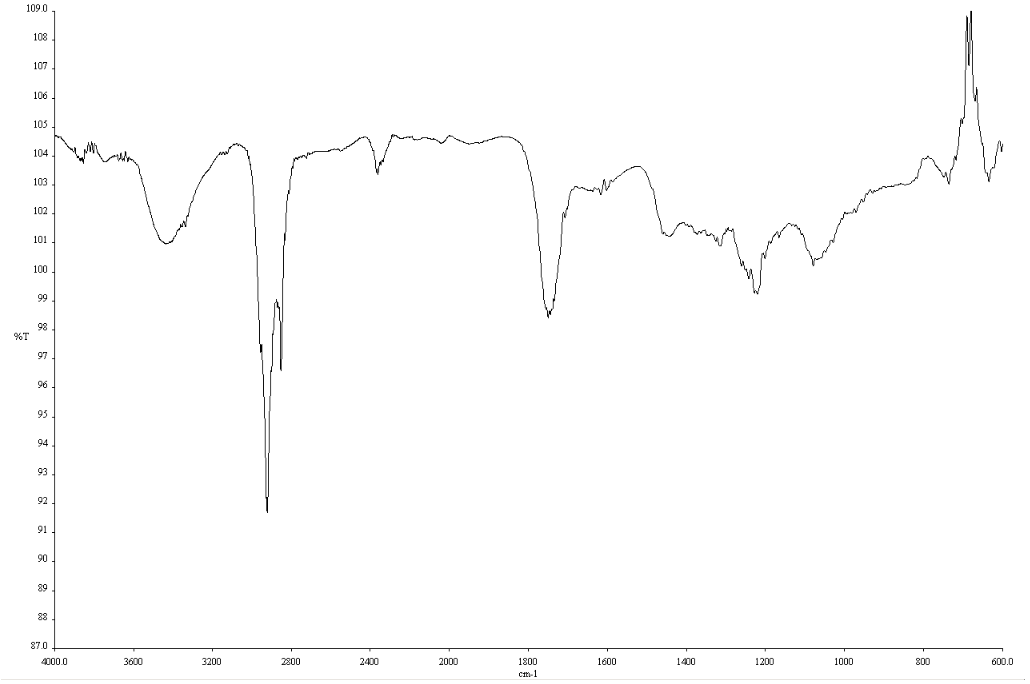

Infrared spectrum (Thin Film, NaCl) of compound **5**.

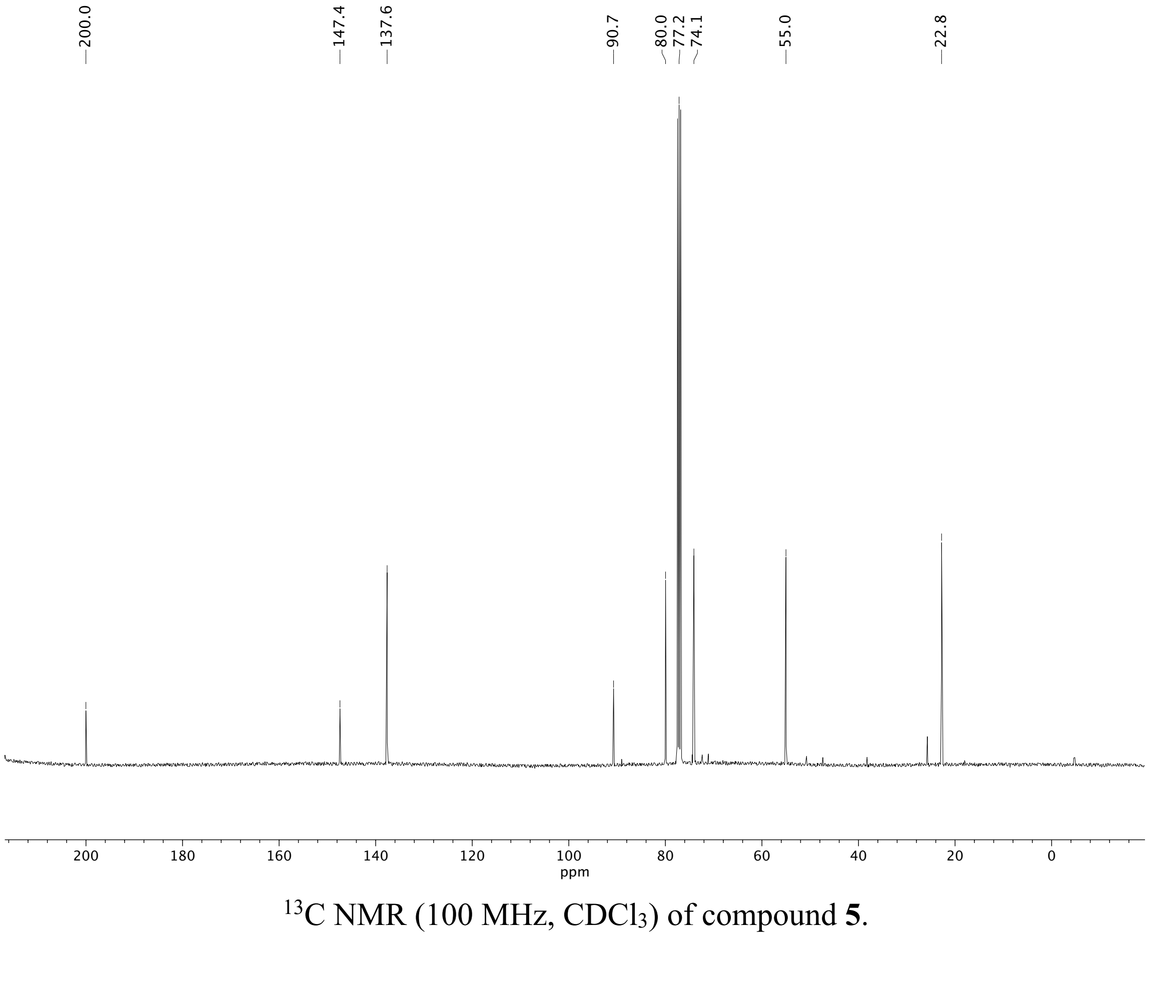

^{13}C NMR (100 MHz, $CDCl_3$) of compound **5**.

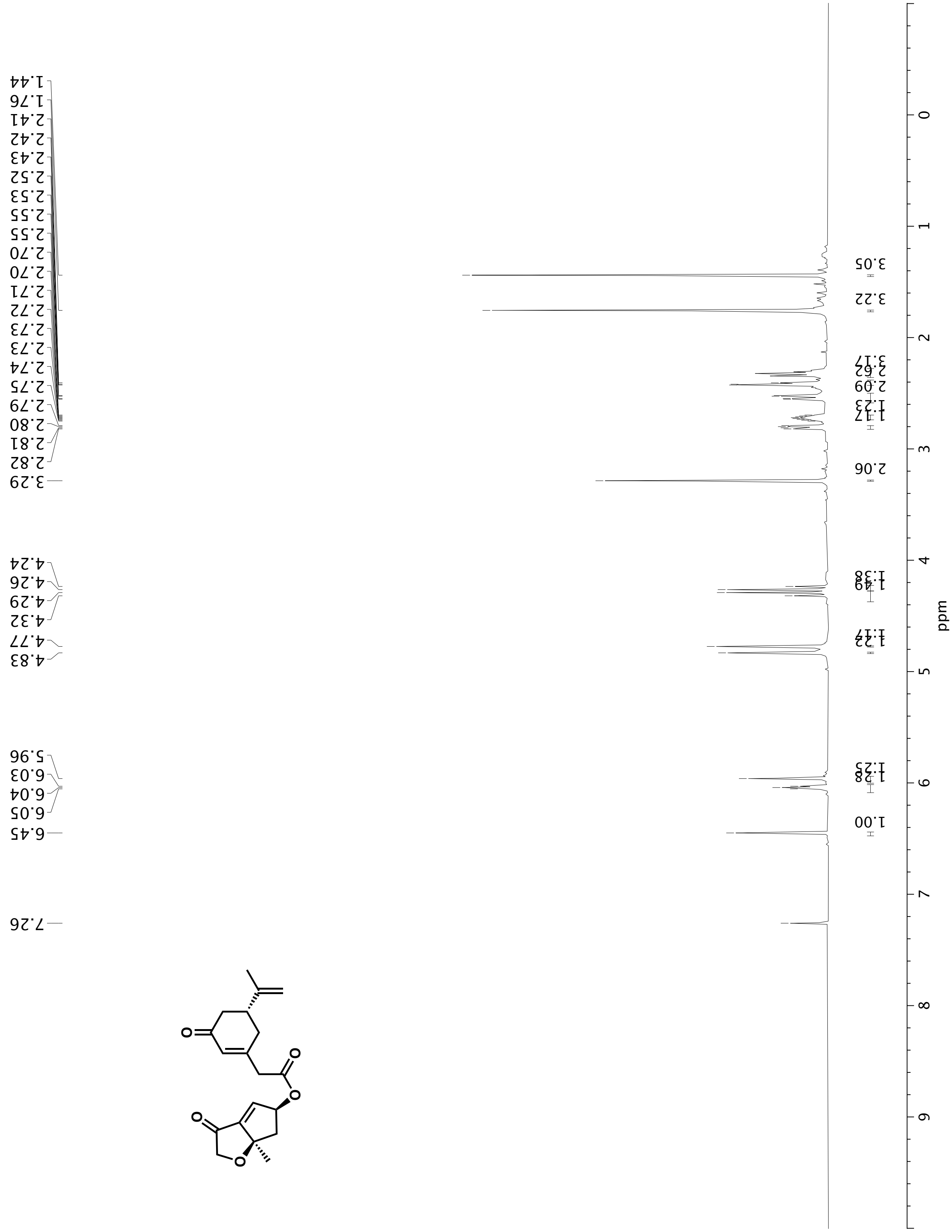

^{1}H NMR (600 MHz, $CDCl_3$) of compound **4**.

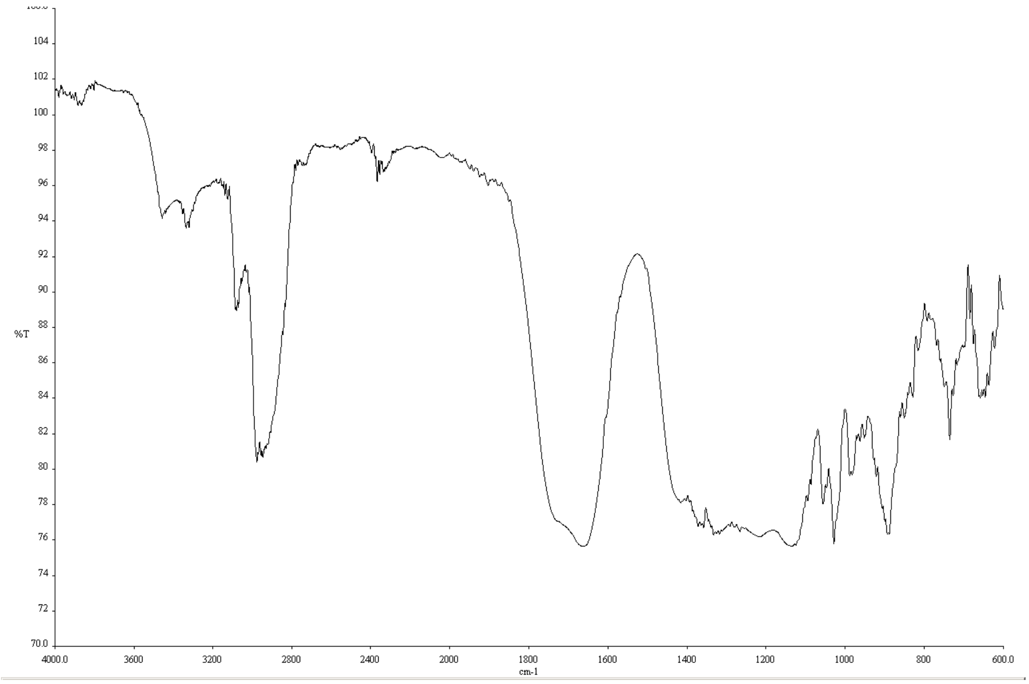

Infrared spectrum (Thin Film, NaCl) of compound **4**.

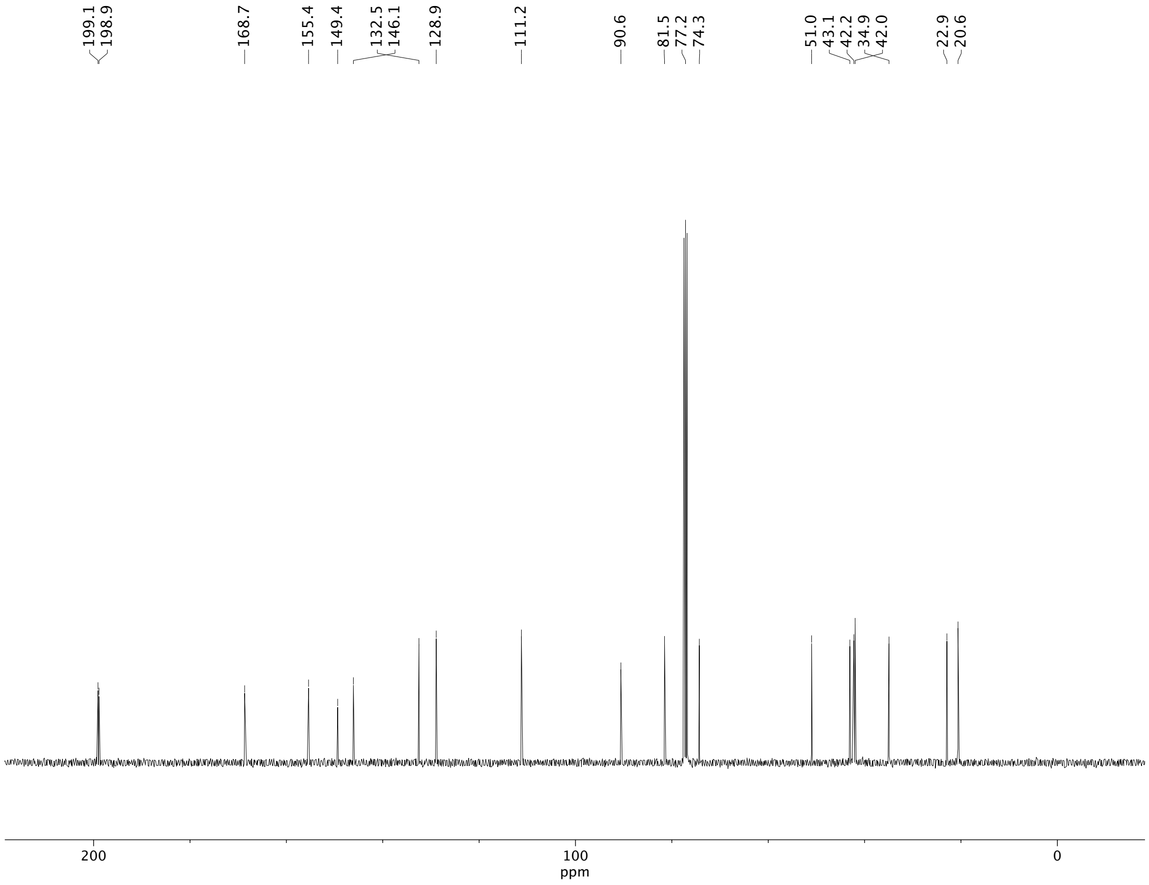

^{13}C NMR (100 MHz, $CDCl_3$) of compound **4**.

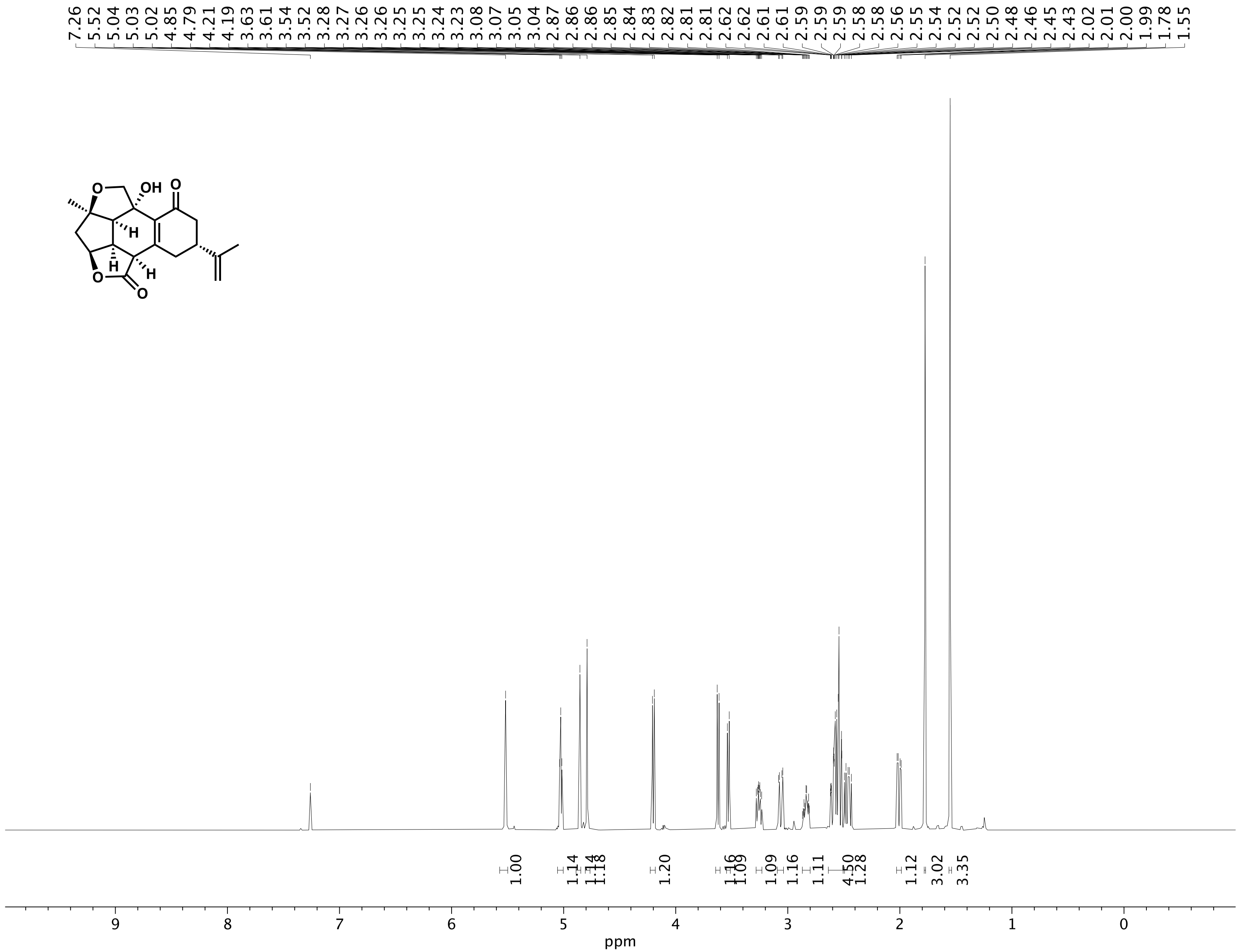

^{1}H NMR (600 MHz, $CDCl_3$) of compound **15**.

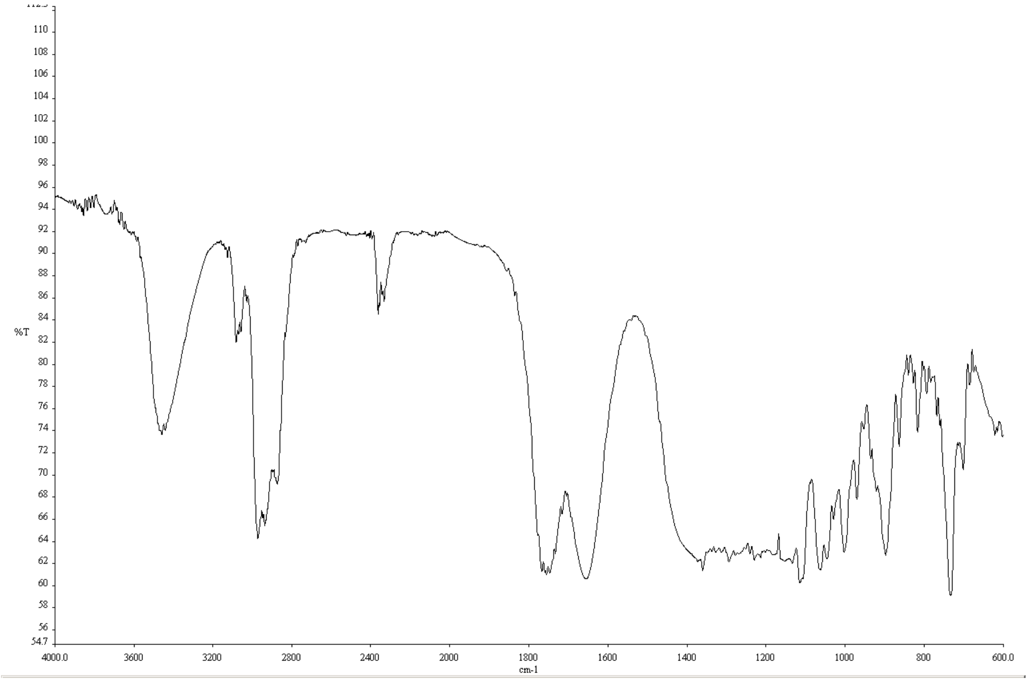

Infrared spectrum (Thin Film, NaCl) of compound **15**.

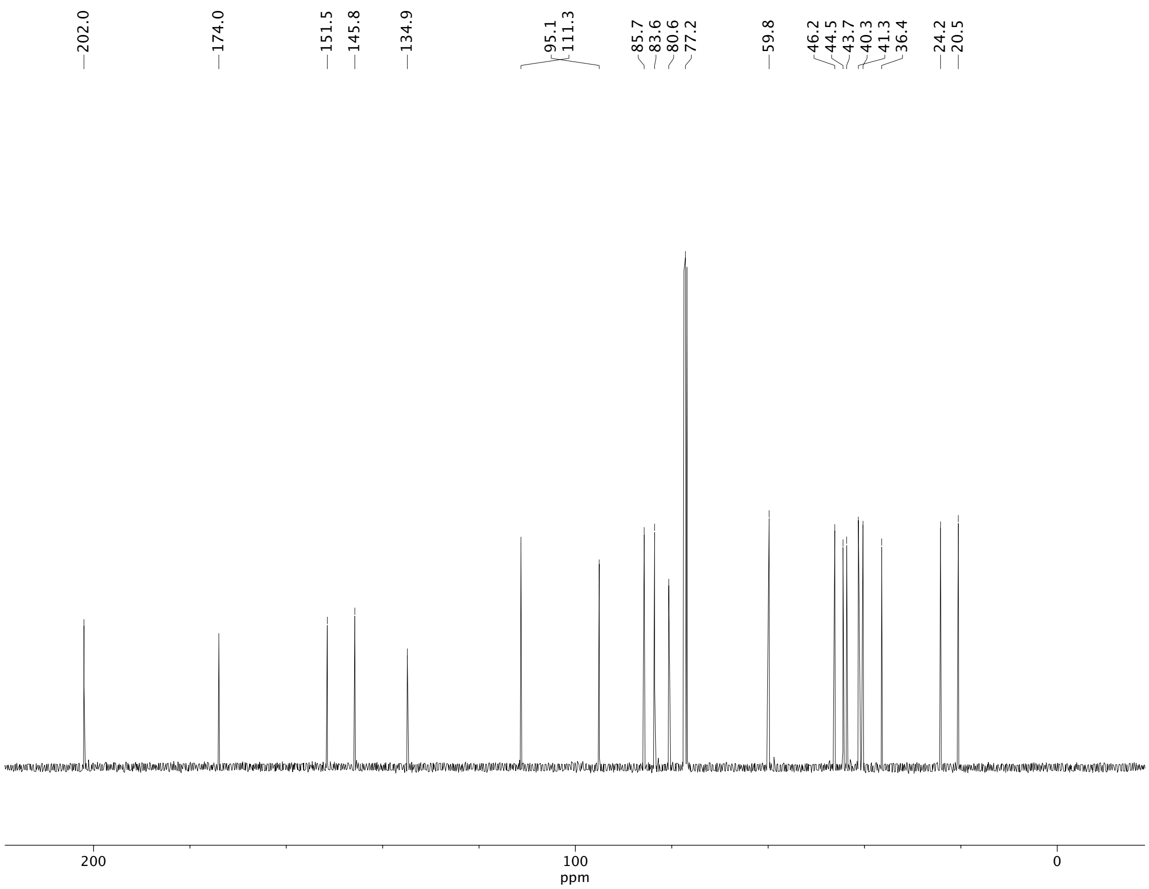

^{13}C NMR (100 MHz, $CDCl_3$) of compound **15**.

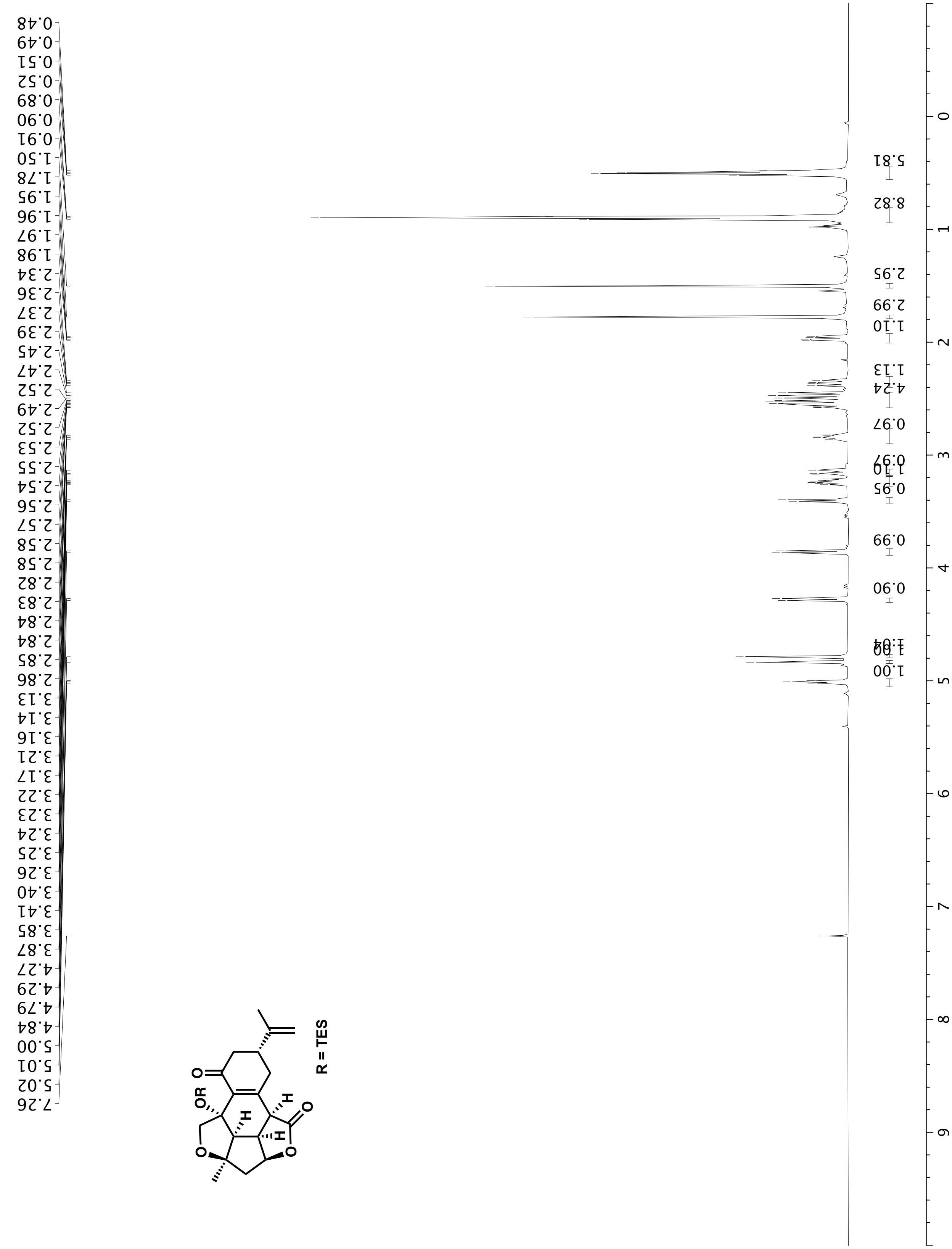

^{1}H NMR (600 MHz, $CDCl_3$) of compound **26**.

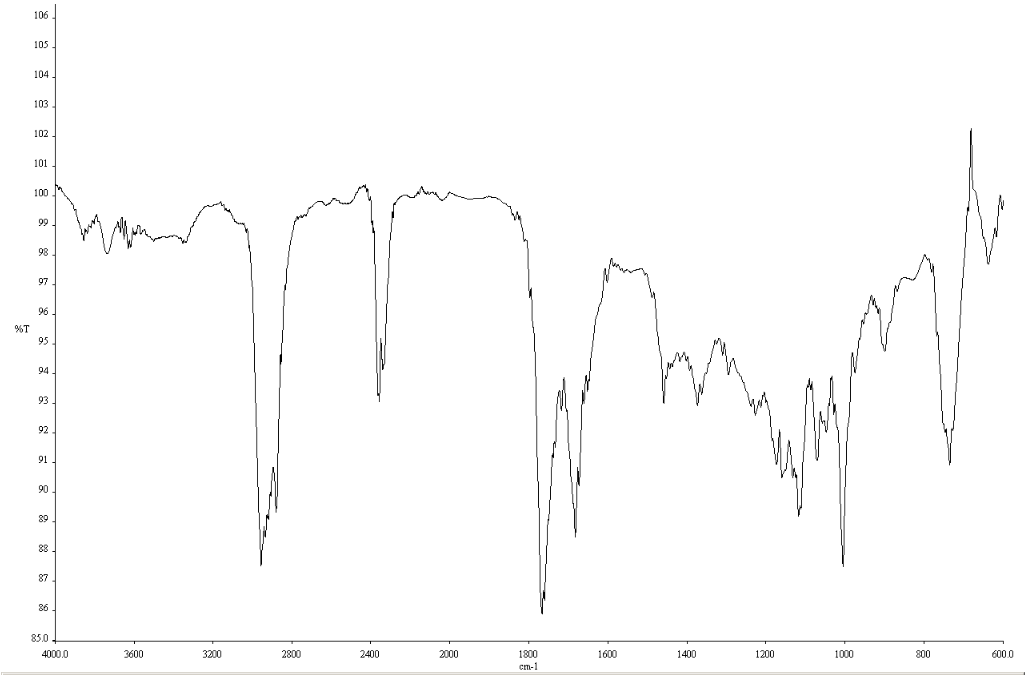

Infrared spectrum (Thin Film, NaCl) of compound **26**.

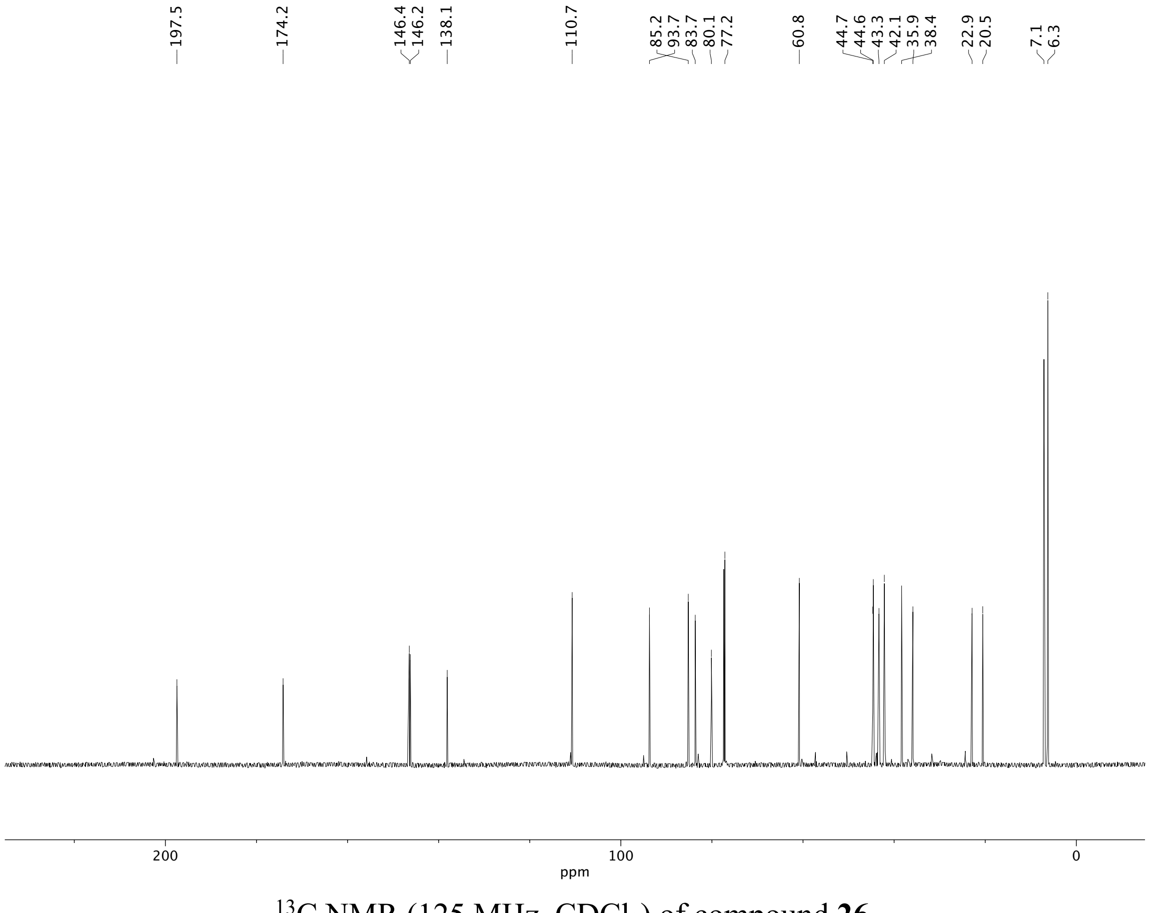

^{13}C NMR (125 MHz, $CDCl_3$) of compound **26**.

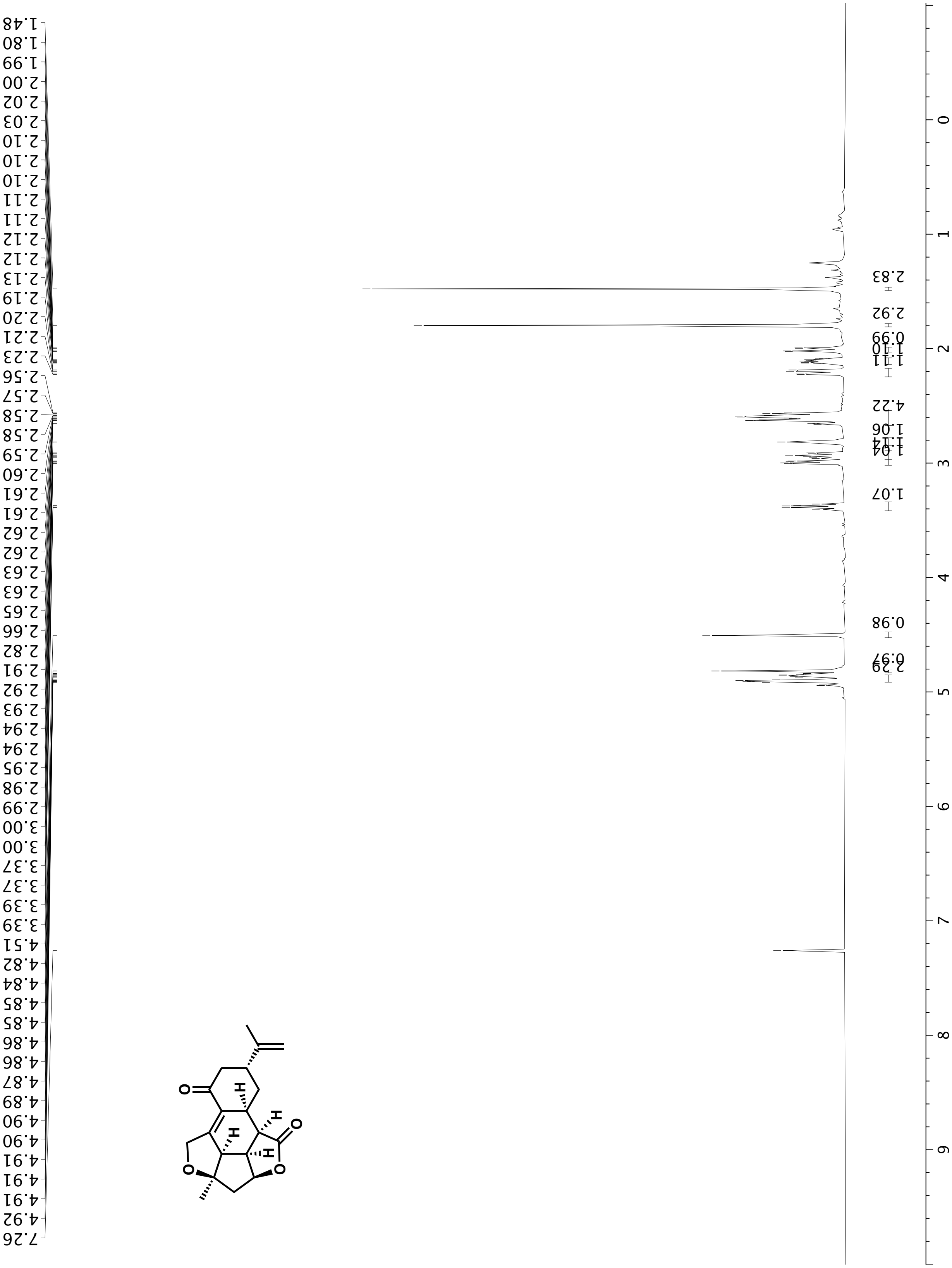

^{1}H NMR (600 MHz, $CDCl_3$) of compound **16**.

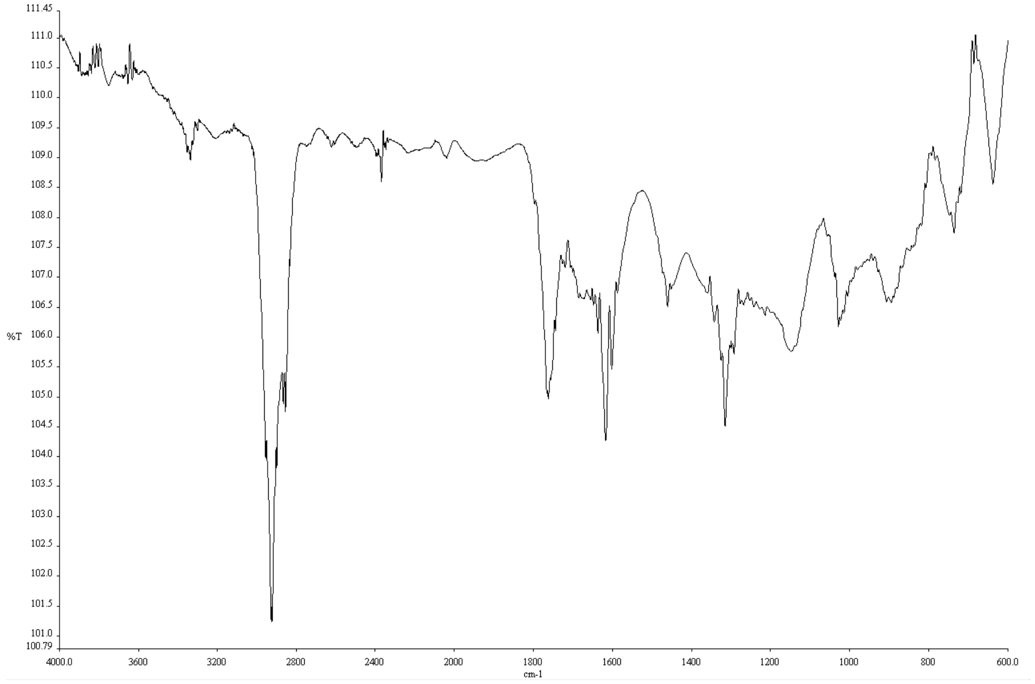

Infrared spectrum (Thin Film, NaCl) of compound **16**.

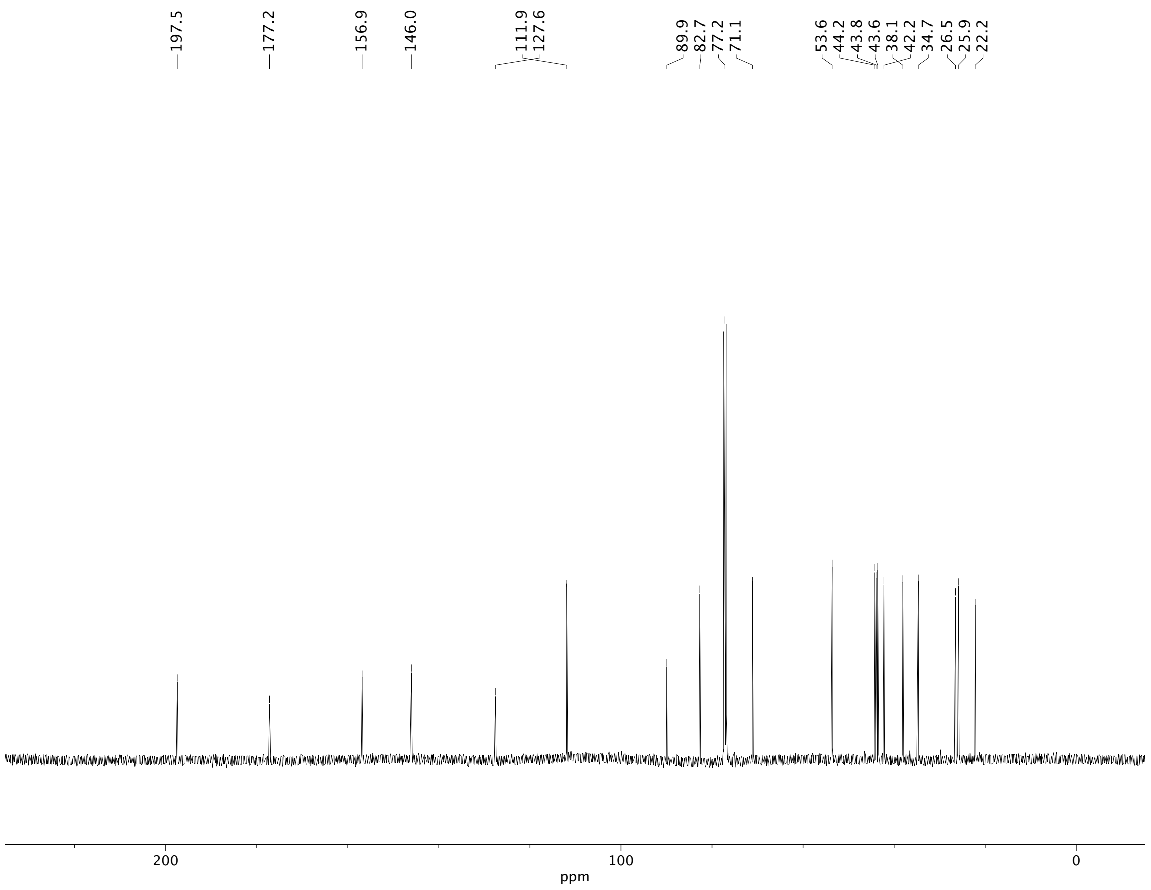

^{13}C NMR (125 MHz, $CDCl_3$) of compound **16**.

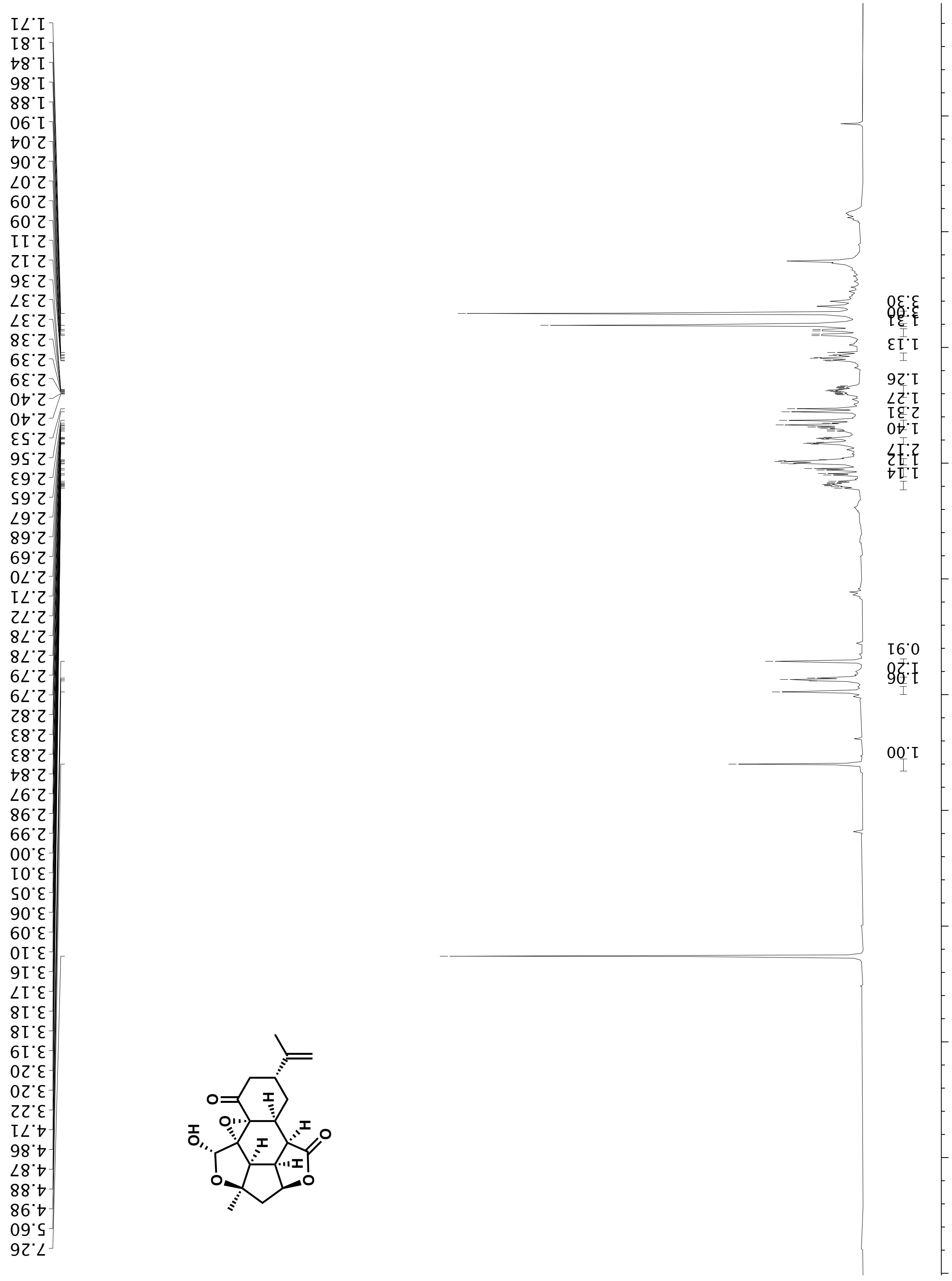

^{1}H NMR (400 MHz, $CDCl_3$) of compound **17**.

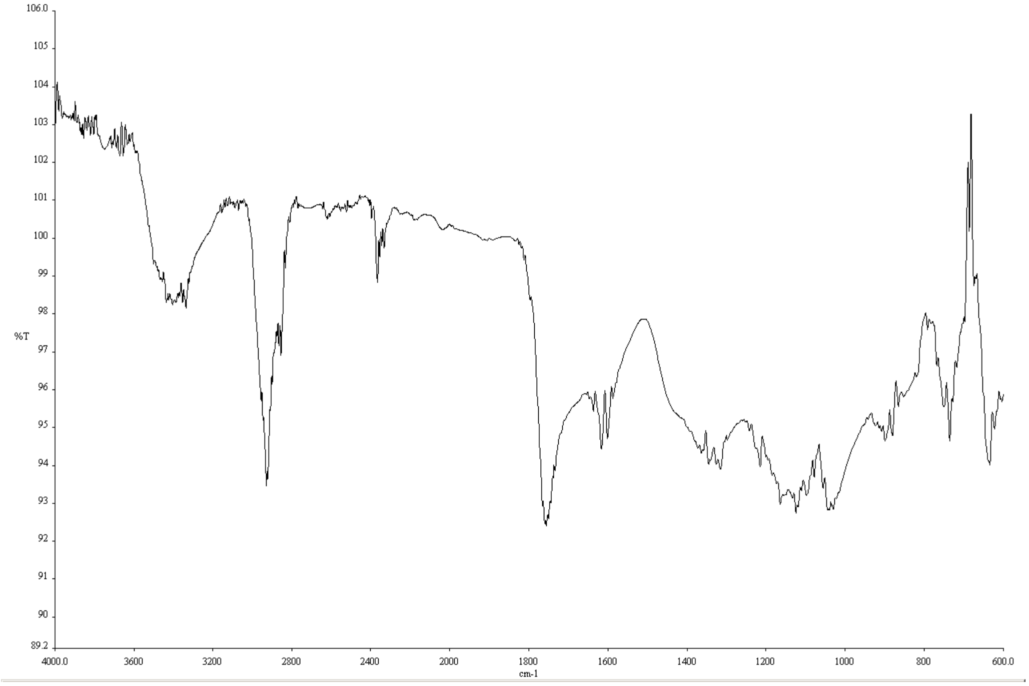

Infrared spectrum (Thin Film, NaCl) of compound **17**.

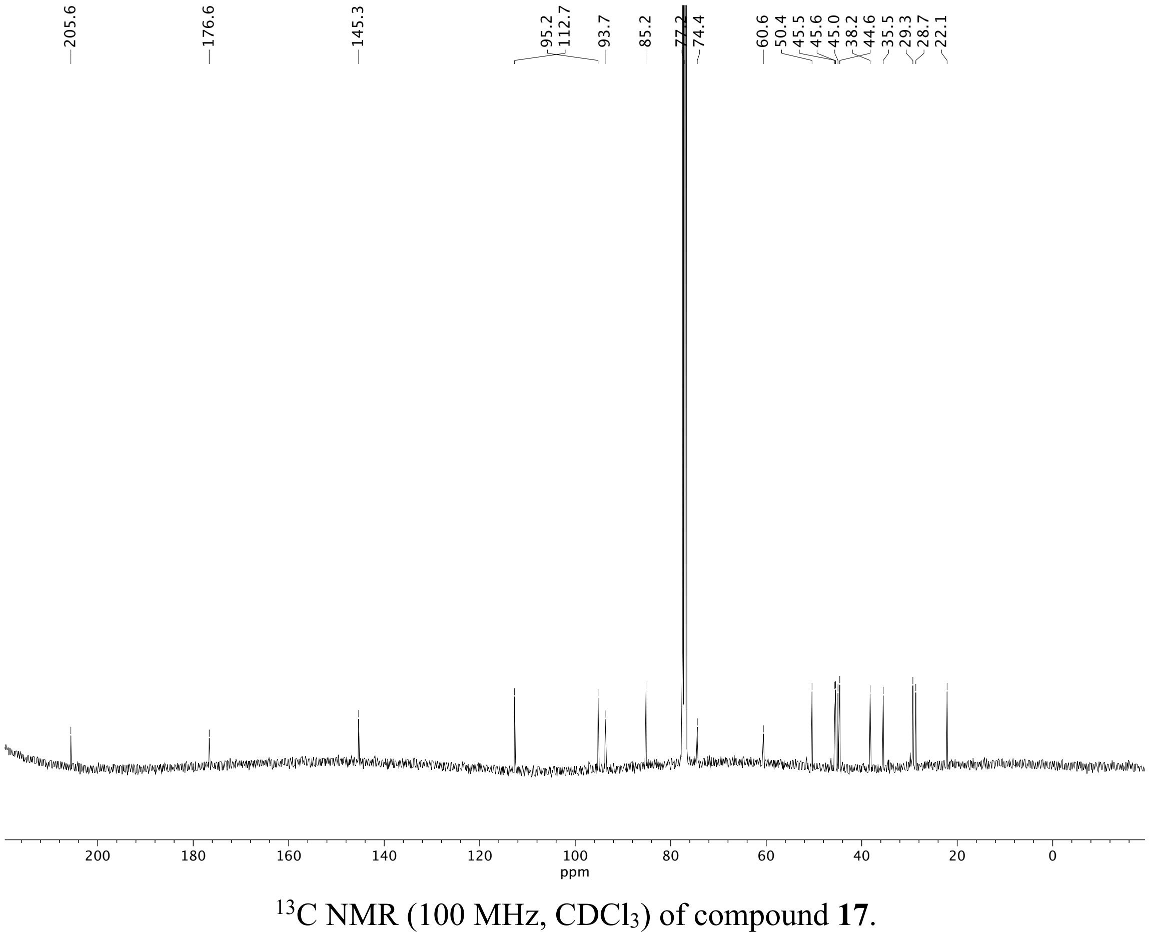

^{13}C NMR (100 MHz, $CDCl_3$) of compound **17**.

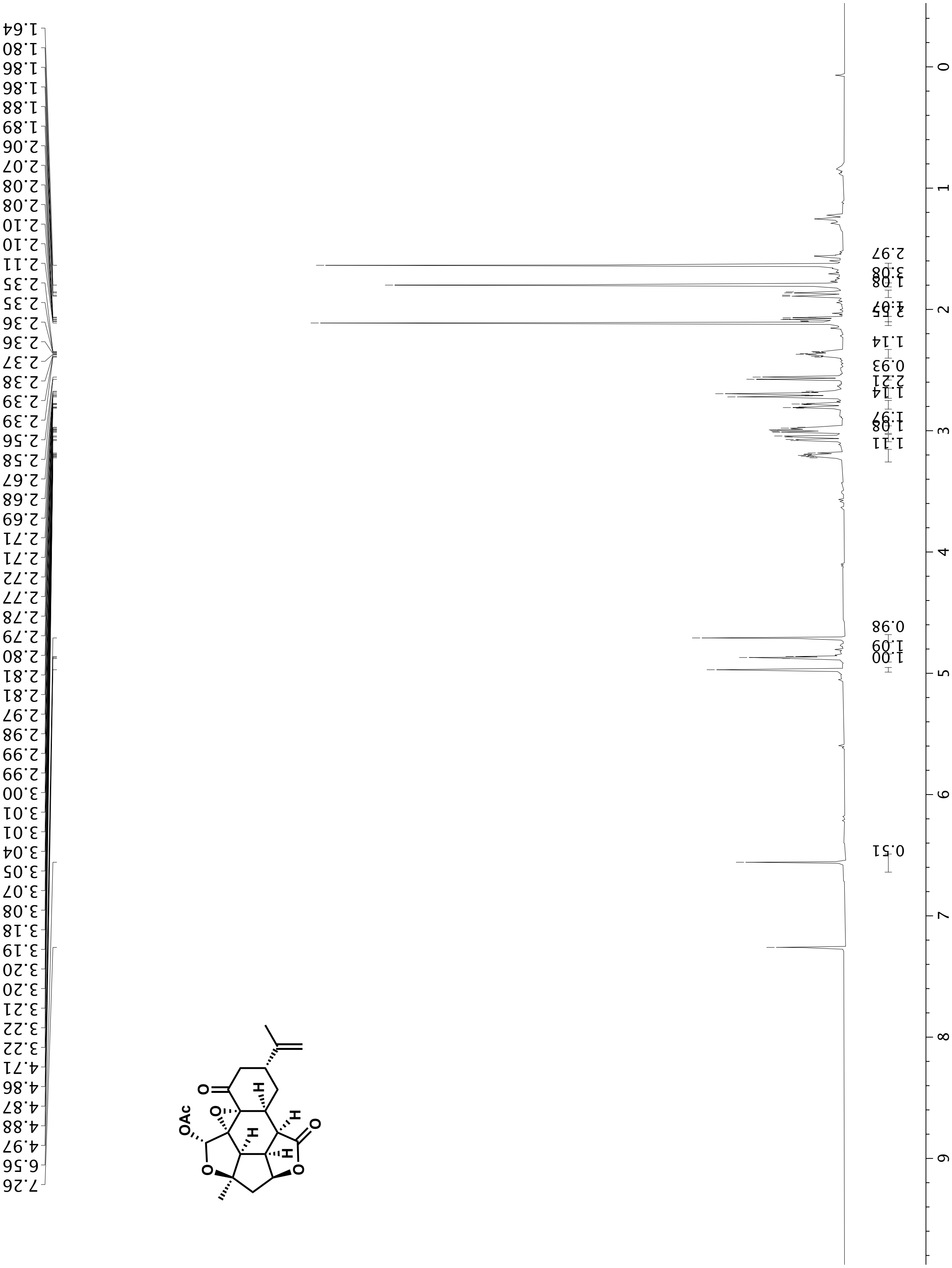

^{1}H NMR (400 MHz, $CDCl_3$) of compound **18**.

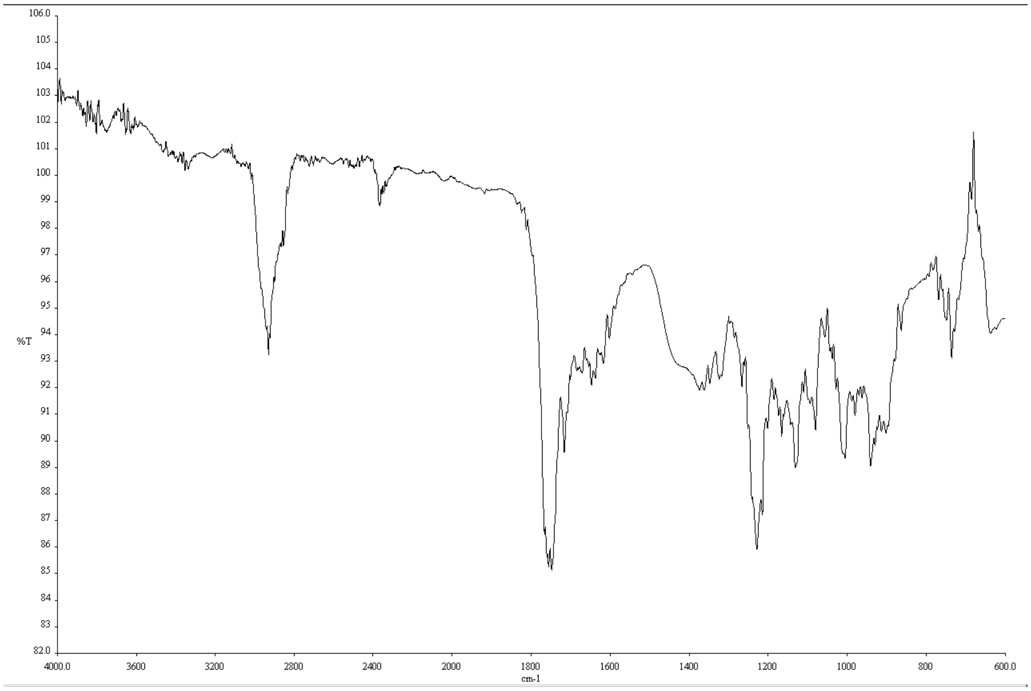

Infrared spectrum (Thin Film, NaCl) of compound **18**.

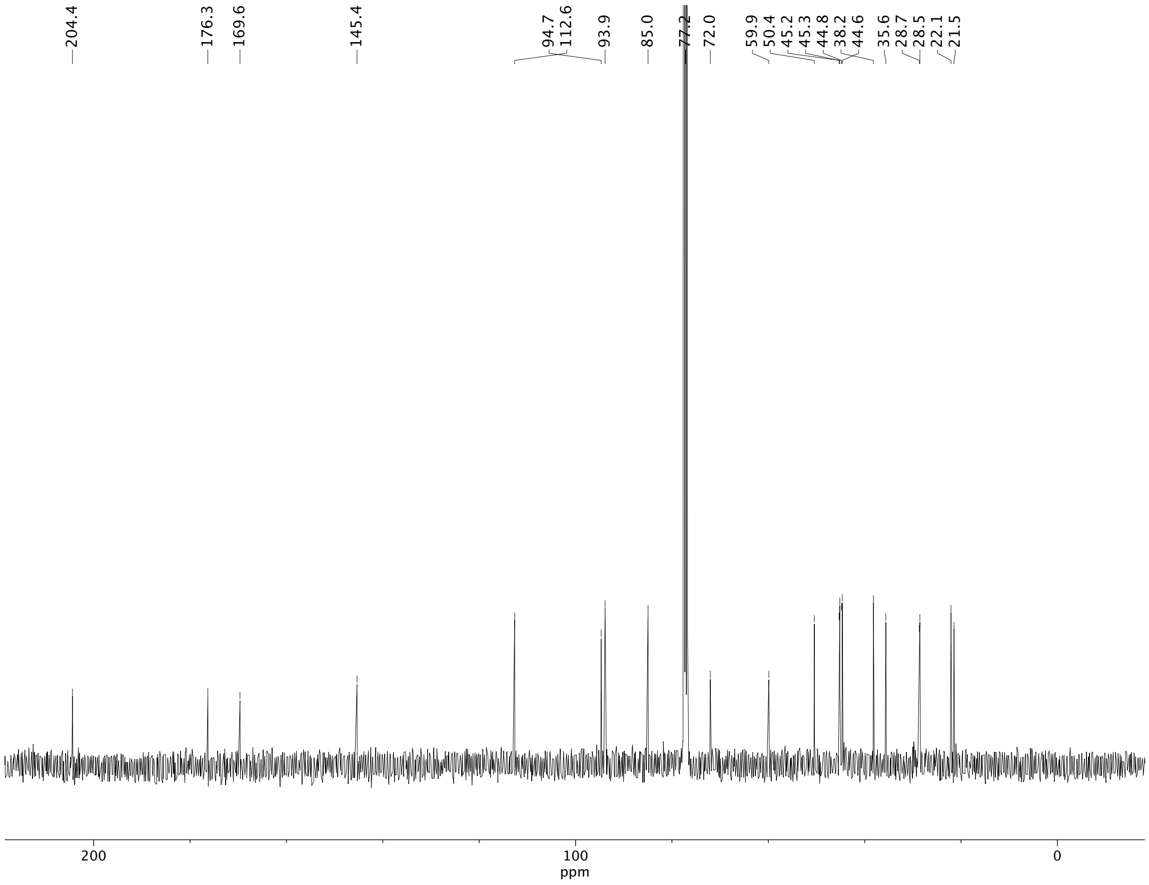

^{13}C NMR (100 MHz, $CDCl_3$) of compound **18**.

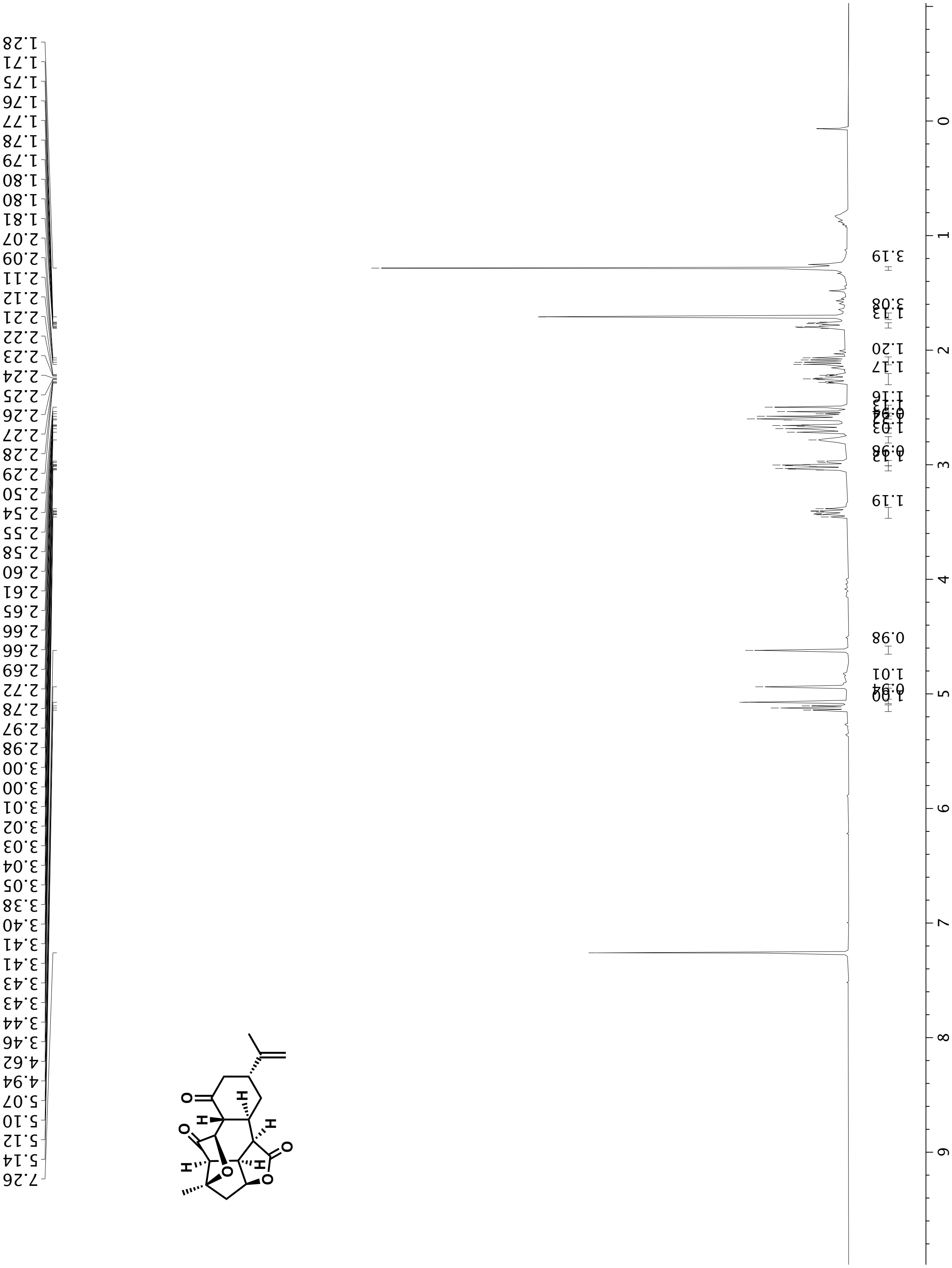

^{1}H NMR (400 MHz, $CDCl_3$) of compound **1**.

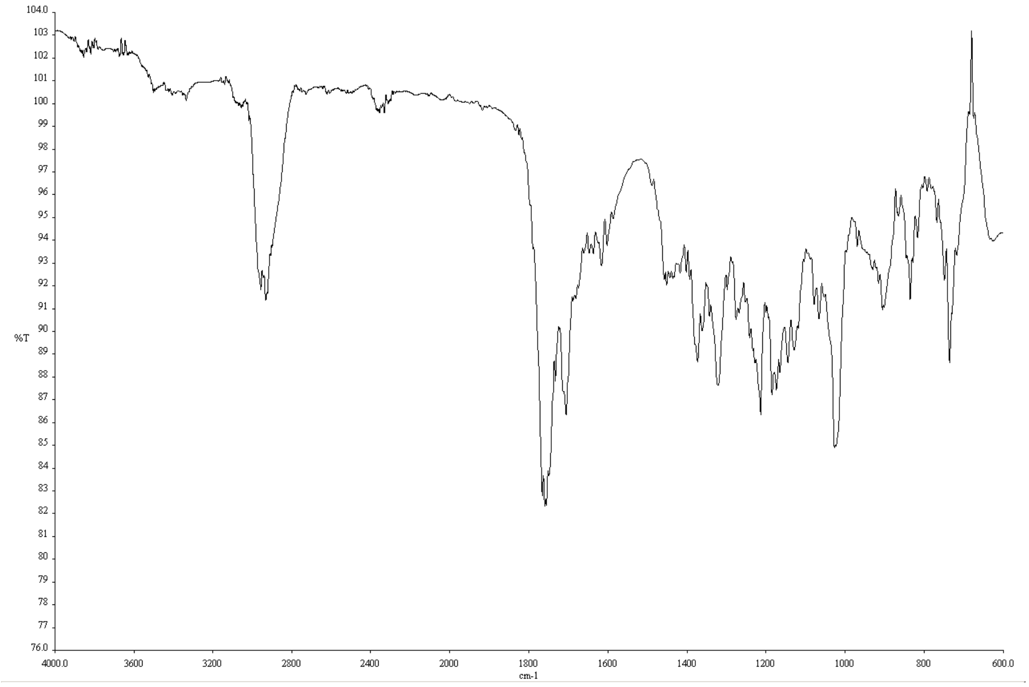

Infrared spectrum (Thin Film, NaCl) of compound **1**.

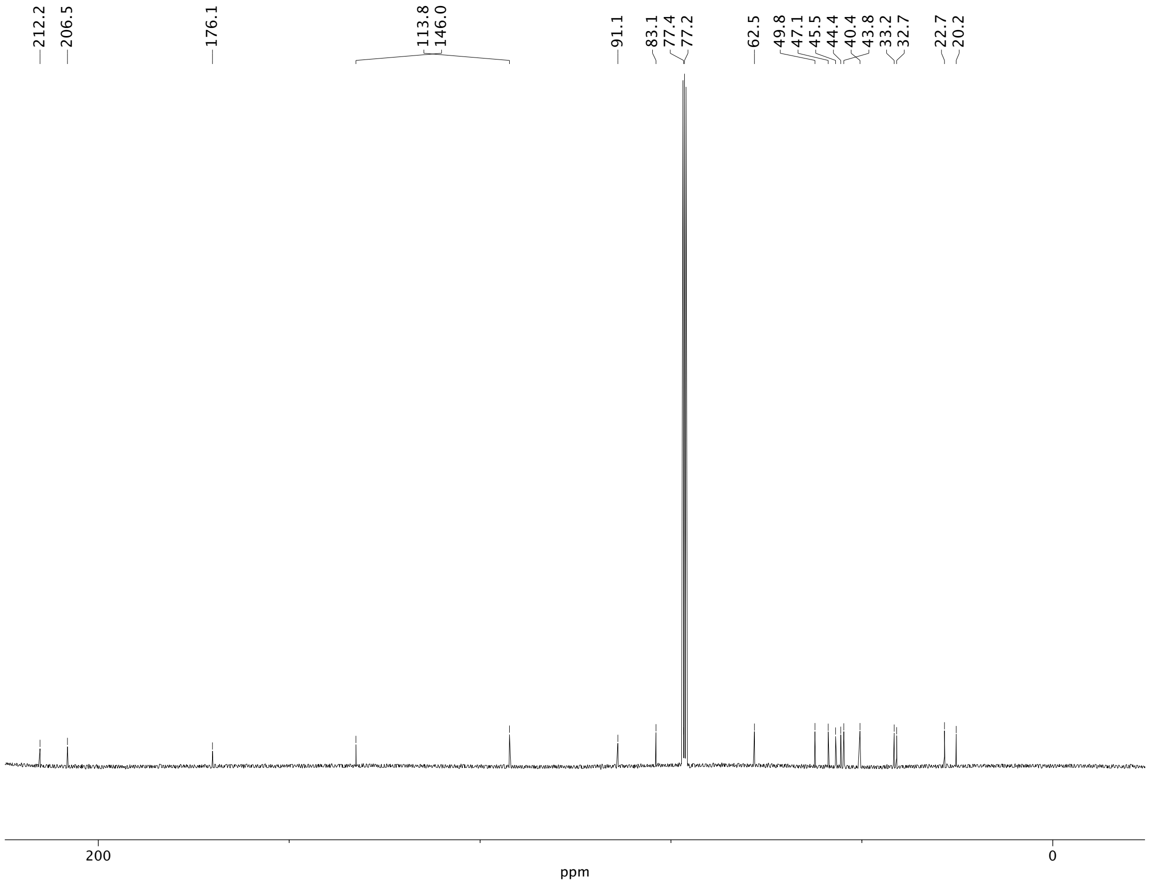

^{13}C NMR (100 MHz, $CDCl_3$) of compound **1**.

X-ray Crystal Structure Data

Figure S3: X-ray coordinate of compound **15**.

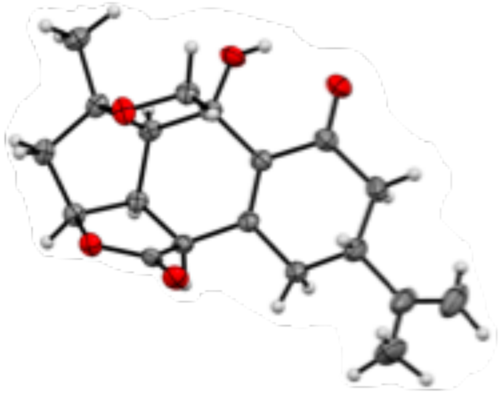

Table S3. Crystal data and structure refinement for V22241.

Identification code	V22241	
Empirical formula	C38 H44 O10	
Formula weight	660.73	
Temperature	100(2) K	
Wavelength	1.54178 Å	
Crystal system	Monoclinic	
Space group	$P2_1$	
Unit cell dimensions	a = 5.4379(6) Å	a= 90°.
	b = 20.759(2) Å	b= 93.475(6)°.
	c = 14.7956(10) Å	g = 90°.
Volume	1667.2(3) Å^3	
Z	2	
Density (calculated)	1.316 Mg/m^3	
Absorption coefficient	0.778 mm^{-1}	
F(000)	704	
Crystal size	0.200 x 0.100 x 0.050 mm^3	
Theta range for data collection	2.992 to 74.571°.	
Index ranges	-6<=h<=6, -25<=k<=25, -18<=l<=18	
Reflections collected	30860	
Independent reflections	6591 [R(int) = 0.0626]	
Completeness to theta = 67.679°	98.8 %	
Absorption correction	Semi-empirical from equivalents	
Max. and min. transmission	0.7538 and 0.5987	

Refinement method	Full-matrix least-squares on F^2
Data / restraints / parameters	6591 / 123 / 472
Goodness-of-fit on F^2	1.043
Final R indices [I>2sigma(I)]	R1 = 0.0388, wR2 = 0.0957
R indices (all data)	R1 = 0.0462, wR2 = 0.0984
Absolute structure parameter	0.12(13)
Extinction coefficient	n/a
Largest diff. peak and hole	0.186 and -0.159 e.Å^{-3}

Table S4. Atomic coordinates (x 10^4) and equivalent isotropic displacement parameters (Å^{2}x 10^3) for V22241. U(eq) is defined as one third of the trace of the orthogonalized U^{ij} tensor.

	x	y	z	U(eq)
O(1)	2700(3)	6720(1)	8394(1)	27(1)
C(1)	3494(5)	6439(1)	9236(2)	25(1)
C(2)	6328(5)	6329(1)	9210(2)	22(1)
O(4)	6584(5)	5571(1)	10834(2)	43(1)
C(3)	6938(5)	5612(1)	9244(2)	23(1)
C(4)	7106(6)	5292(2)	10141(2)	34(1)
O(5)	7629(4)	6666(1)	9937(1)	29(1)
C(5)	7989(9)	4600(2)	10171(2)	49(1)
C(6)	6818(8)	4217(2)	9379(2)	39(1)
C(16)	7455(9)	3504(2)	9376(3)	51(1)
C(17)	9389(9)	3264(2)	9859(3)	58(1)
C(18)	5771(12)	3102(2)	8797(3)	68(1)
C(7)	7501(6)	4538(1)	8501(2)	28(1)
C(8)	7088(5)	5255(1)	8494(2)	22(1)
C(9)	6734(5)	5546(1)	7575(2)	21(1)
C(10)	4061(5)	5489(1)	7203(2)	22(1)
O(2)	3443(4)	6002(1)	6691(1)	24(1)
O(3)	2636(4)	5057(1)	7311(1)	30(1)
C(11)	5399(5)	6489(1)	6739(2)	23(1)
C(12)	4431(5)	7127(1)	7064(2)	26(1)
C(13)	4713(5)	7103(1)	8087(2)	23(1)
C(19)	4785(6)	7765(1)	8531(2)	32(1)
C(14)	6959(5)	6675(1)	8331(2)	20(1)
C(15)	7323(5)	6258(1)	7481(2)	21(1)
O(201)	-2721(3)	3099(1)	4780(1)	26(1)
C(201)	-2411(5)	3593(1)	4137(2)	22(1)
C(202)	340(5)	3599(1)	3953(2)	22(1)
O(204)	476(6)	4016(1)	2088(2)	46(1)
C(203)	1184(5)	4260(1)	3646(2)	24(1)
C(204)	1242(7)	4400(2)	2668(2)	34(1)

O(205)	857(4)	3101(1)	3328(1)	25(1)
C(205)	2297(9)	5037(2)	2402(2)	47(1)
C(206)	1569(8)	5574(2)	3041(2)	44(1)
C(216)	2690(30)	6220(6)	2760(11)	52(3)
C(217)	4390(17)	6547(4)	3206(8)	56(3)
C(218)	1320(30)	6465(5)	1912(7)	76(4)
C(16B)	1970(40)	6256(9)	2640(20)	37(4)
C(17B)	290(30)	6633(6)	2235(13)	45(4)
C(18B)	4630(30)	6430(7)	2733(18)	50(4)
C(207)	2438(7)	5404(2)	4000(2)	36(1)
C(208)	1749(5)	4727(1)	4253(2)	26(1)
C(209)	1661(5)	4603(1)	5250(2)	25(1)
C(210)	-888(5)	4742(1)	5581(2)	26(1)
O(202)	-1542(4)	4272(1)	6152(1)	27(1)
O(203)	-2238(4)	5184(1)	5384(2)	33(1)
C(211)	464(5)	3816(1)	6337(2)	26(1)
C(212)	-466(6)	3128(1)	6262(2)	29(1)
C(213)	-401(5)	2938(1)	5261(2)	24(1)
C(219)	15(6)	2217(1)	5126(2)	30(1)
C(214)	1552(5)	3379(1)	4862(2)	22(1)
C(215)	2197(5)	3910(1)	5567(2)	24(1)

Table S5. Bond lengths [Å] and angles [°] for V22241.

O(1)-C(1)	1.419(3)
O(1)-C(13)	1.449(3)
C(1)-C(2)	1.561(4)
C(1)-H(1A)	0.9900
C(1)-H(1B)	0.9900
C(2)-O(5)	1.433(3)
C(2)-C(3)	1.524(4)
C(2)-C(14)	1.543(4)
O(4)-C(4)	1.225(4)
C(3)-C(8)	1.342(4)
C(3)-C(4)	1.483(4)
C(4)-C(5)	1.515(4)
O(5)-H(5O)	0.83(3)
C(5)-C(6)	1.523(5)
C(5)-H(5A)	0.9900
C(5)-H(5B)	0.9900
C(6)-C(16)	1.520(4)
C(6)-C(7)	1.526(4)
C(6)-H(6)	1.0000
C(16)-C(17)	1.333(7)
C(16)-C(18)	1.474(7)
C(17)-H(17A)	0.9500
C(17)-H(17B)	0.9500
C(18)-H(18A)	0.9800
C(18)-H(18B)	0.9800
C(18)-H(18C)	0.9800
C(7)-C(8)	1.506(4)
C(7)-H(7A)	0.9900
C(7)-H(7B)	0.9900
C(8)-C(9)	1.489(4)
C(9)-C(15)	1.522(4)
C(9)-C(10)	1.527(4)
C(9)-H(9)	1.0000

C(10)-O(3)	1.203(3)
C(10)-O(2)	1.338(3)
O(2)-C(11)	1.465(3)
C(11)-C(12)	1.515(4)
C(11)-C(15)	1.545(4)
C(11)-H(11)	1.0000
C(12)-C(13)	1.513(4)
C(12)-H(12A)	0.9900
C(12)-H(12B)	0.9900
C(13)-C(19)	1.522(4)
C(13)-C(14)	1.536(4)
C(19)-H(19A)	0.9800
C(19)-H(19B)	0.9800
C(19)-H(19C)	0.9800
C(14)-C(15)	1.548(4)
C(14)-H(14)	1.0000
C(15)-H(15)	1.0000
O(201)-C(201)	1.416(3)
O(201)-C(213)	1.450(3)
C(201)-C(202)	1.537(4)
C(201)-H(20A)	0.9900
C(201)-H(20B)	0.9900
C(202)-O(205)	1.427(3)
C(202)-C(203)	1.525(4)
C(202)-C(214)	1.532(4)
O(204)-C(204)	1.224(4)
C(203)-C(208)	1.345(4)
C(203)-C(204)	1.479(4)
C(204)-C(205)	1.504(5)
O(205)-H(05O)	0.82(3)
C(205)-C(206)	1.528(5)
C(205)-H(20C)	0.9900
C(205)-H(20D)	0.9900
C(206)-C(207)	1.510(5)
C(206)-C(216)	1.541(10)

C(206)-C(16B)	1.556(16)
C(206)-H(206)	1.0000
C(216)-C(217)	1.296(14)
C(216)-C(218)	1.507(14)
C(217)-H(21A)	0.9500
C(217)-H(21B)	0.9500
C(218)-H(21C)	0.9800
C(218)-H(21D)	0.9800
C(218)-H(21E)	0.9800
C(16B)-C(17B)	1.32(2)
C(16B)-C(18B)	1.49(2)
C(17B)-H(17C)	0.9500
C(17B)-H(17D)	0.9500
C(18B)-H(18D)	0.9800
C(18B)-H(18E)	0.9800
C(18B)-H(18F)	0.9800
C(207)-C(208)	1.506(4)
C(207)-H(20E)	0.9900
C(207)-H(20F)	0.9900
C(208)-C(209)	1.502(4)
C(209)-C(210)	1.526(4)
C(209)-C(215)	1.536(4)
C(209)-H(209)	1.0000
C(210)-O(203)	1.200(4)
C(210)-O(202)	1.352(4)
O(202)-C(211)	1.458(4)
C(211)-C(212)	1.518(4)
C(211)-C(215)	1.534(4)
C(211)-H(211)	1.0000
C(212)-C(213)	1.535(4)
C(212)-H(21F)	0.9900
C(212)-H(21G)	0.9900
C(213)-C(219)	1.530(4)
C(213)-C(214)	1.545(4)
C(219)-H(21H)	0.9800

C(219)-H(21I)	0.9800
C(219)-H(21J)	0.9800
C(214)-C(215)	1.543(4)
C(214)-H(214)	1.0000
C(215)-H(215)	1.0000

C(1)-O(1)-C(13)	107.8(2)
O(1)-C(1)-C(2)	106.7(2)
O(1)-C(1)-H(1A)	110.4
C(2)-C(1)-H(1A)	110.4
O(1)-C(1)-H(1B)	110.4
C(2)-C(1)-H(1B)	110.4
H(1A)-C(1)-H(1B)	108.6
O(5)-C(2)-C(3)	110.8(2)
O(5)-C(2)-C(14)	106.3(2)
C(3)-C(2)-C(14)	115.0(2)
O(5)-C(2)-C(1)	110.7(2)
C(3)-C(2)-C(1)	110.8(2)
C(14)-C(2)-C(1)	102.9(2)
C(8)-C(3)-C(4)	119.3(2)
C(8)-C(3)-C(2)	122.4(3)
C(4)-C(3)-C(2)	117.9(2)
O(4)-C(4)-C(3)	122.2(3)
O(4)-C(4)-C(5)	120.8(3)
C(3)-C(4)-C(5)	117.0(3)
C(2)-O(5)-H(5O)	105(3)
C(4)-C(5)-C(6)	110.8(3)
C(4)-C(5)-H(5A)	109.5
C(6)-C(5)-H(5A)	109.5
C(4)-C(5)-H(5B)	109.5
C(6)-C(5)-H(5B)	109.5
H(5A)-C(5)-H(5B)	108.1
C(16)-C(6)-C(5)	115.2(3)
C(16)-C(6)-C(7)	110.8(3)
C(5)-C(6)-C(7)	108.4(3)

C(16)-C(6)-H(6)	107.4
C(5)-C(6)-H(6)	107.4
C(7)-C(6)-H(6)	107.4
C(17)-C(16)-C(18)	122.8(4)
C(17)-C(16)-C(6)	122.4(4)
C(18)-C(16)-C(6)	114.8(4)
C(16)-C(17)-H(17A)	120.0
C(16)-C(17)-H(17B)	120.0
H(17A)-C(17)-H(17B)	120.0
C(16)-C(18)-H(18A)	109.5
C(16)-C(18)-H(18B)	109.5
H(18A)-C(18)-H(18B)	109.5
C(16)-C(18)-H(18C)	109.5
H(18A)-C(18)-H(18C)	109.5
H(18B)-C(18)-H(18C)	109.5
C(8)-C(7)-C(6)	113.2(2)
C(8)-C(7)-H(7A)	108.9
C(6)-C(7)-H(7A)	108.9
C(8)-C(7)-H(7B)	108.9
C(6)-C(7)-H(7B)	108.9
H(7A)-C(7)-H(7B)	107.8
C(3)-C(8)-C(9)	121.4(2)
C(3)-C(8)-C(7)	123.9(3)
C(9)-C(8)-C(7)	114.7(2)
C(8)-C(9)-C(15)	117.4(2)
C(8)-C(9)-C(10)	111.4(2)
C(15)-C(9)-C(10)	104.0(2)
C(8)-C(9)-H(9)	107.8
C(15)-C(9)-H(9)	107.8
C(10)-C(9)-H(9)	107.8
O(3)-C(10)-O(2)	121.8(2)
O(3)-C(10)-C(9)	128.0(2)
O(2)-C(10)-C(9)	110.1(2)
C(10)-O(2)-C(11)	111.3(2)
O(2)-C(11)-C(12)	110.7(2)

O(2)-C(11)-C(15)	106.3(2)
C(12)-C(11)-C(15)	106.2(2)
O(2)-C(11)-H(11)	111.2
C(12)-C(11)-H(11)	111.2
C(15)-C(11)-H(11)	111.2
C(13)-C(12)-C(11)	105.8(2)
C(13)-C(12)-H(12A)	110.6
C(11)-C(12)-H(12A)	110.6
C(13)-C(12)-H(12B)	110.6
C(11)-C(12)-H(12B)	110.6
H(12A)-C(12)-H(12B)	108.7
O(1)-C(13)-C(12)	107.4(2)
O(1)-C(13)-C(19)	111.2(2)
C(12)-C(13)-C(19)	113.6(2)
O(1)-C(13)-C(14)	102.3(2)
C(12)-C(13)-C(14)	106.5(2)
C(19)-C(13)-C(14)	115.0(2)
C(13)-C(19)-H(19A)	109.5
C(13)-C(19)-H(19B)	109.5
H(19A)-C(19)-H(19B)	109.5
C(13)-C(19)-H(19C)	109.5
H(19A)-C(19)-H(19C)	109.5
H(19B)-C(19)-H(19C)	109.5
C(13)-C(14)-C(2)	104.7(2)
C(13)-C(14)-C(15)	105.8(2)
C(2)-C(14)-C(15)	118.1(2)
C(13)-C(14)-H(14)	109.3
C(2)-C(14)-H(14)	109.3
C(15)-C(14)-H(14)	109.3
C(9)-C(15)-C(11)	103.2(2)
C(9)-C(15)-C(14)	115.5(2)
C(11)-C(15)-C(14)	106.9(2)
C(9)-C(15)-H(15)	110.3
C(11)-C(15)-H(15)	110.3
C(14)-C(15)-H(15)	110.3

C(201)-O(201)-C(213)	111.2(2)
O(201)-C(201)-C(202)	106.3(2)
O(201)-C(201)-H(20A)	110.5
C(202)-C(201)-H(20A)	110.5
O(201)-C(201)-H(20B)	110.5
C(202)-C(201)-H(20B)	110.5
H(20A)-C(201)-H(20B)	108.7
O(205)-C(202)-C(203)	112.6(2)
O(205)-C(202)-C(214)	105.2(2)
C(203)-C(202)-C(214)	114.1(2)
O(205)-C(202)-C(201)	110.0(2)
C(203)-C(202)-C(201)	111.9(2)
C(214)-C(202)-C(201)	102.3(2)
C(208)-C(203)-C(204)	119.6(3)
C(208)-C(203)-C(202)	120.8(3)
C(204)-C(203)-C(202)	119.5(2)
O(204)-C(204)-C(203)	122.1(3)
O(204)-C(204)-C(205)	120.6(3)
C(203)-C(204)-C(205)	117.3(3)
C(202)-O(205)-H(05O)	108(3)
C(204)-C(205)-C(206)	111.3(3)
C(204)-C(205)-H(20C)	109.4
C(206)-C(205)-H(20C)	109.4
C(204)-C(205)-H(20D)	109.4
C(206)-C(205)-H(20D)	109.4
H(20C)-C(205)-H(20D)	108.0
C(207)-C(206)-C(205)	109.4(3)
C(207)-C(206)-C(216)	110.6(6)
C(205)-C(206)-C(216)	110.3(8)
C(207)-C(206)-C(16B)	121.8(11)
C(205)-C(206)-C(16B)	112.4(12)
C(207)-C(206)-H(206)	108.8
C(205)-C(206)-H(206)	108.8
C(216)-C(206)-H(206)	108.8
C(217)-C(216)-C(218)	123.2(8)

C(217)-C(216)-C(206)	126.9(9)
C(218)-C(216)-C(206)	109.7(9)
C(216)-C(217)-H(21A)	120.0
C(216)-C(217)-H(21B)	120.0
H(21A)-C(217)-H(21B)	120.0
C(216)-C(218)-H(21C)	109.5
C(216)-C(218)-H(21D)	109.5
H(21C)-C(218)-H(21D)	109.5
C(216)-C(218)-H(21E)	109.5
H(21C)-C(218)-H(21E)	109.5
H(21D)-C(218)-H(21E)	109.5
C(17B)-C(16B)-C(18B)	122.7(14)
C(17B)-C(16B)-C(206)	127.2(15)
C(18B)-C(16B)-C(206)	110.1(12)
C(16B)-C(17B)-H(17C)	120.0
C(16B)-C(17B)-H(17D)	120.0
H(17C)-C(17B)-H(17D)	120.0
C(16B)-C(18B)-H(18D)	109.5
C(16B)-C(18B)-H(18E)	109.5
H(18D)-C(18B)-H(18E)	109.5
C(16B)-C(18B)-H(18F)	109.5
H(18D)-C(18B)-H(18F)	109.5
H(18E)-C(18B)-H(18F)	109.5
C(208)-C(207)-C(206)	112.5(3)
C(208)-C(207)-H(20E)	109.1
C(206)-C(207)-H(20E)	109.1
C(208)-C(207)-H(20F)	109.1
C(206)-C(207)-H(20F)	109.1
H(20E)-C(207)-H(20F)	107.8
C(203)-C(208)-C(209)	120.8(3)
C(203)-C(208)-C(207)	123.9(3)
C(209)-C(208)-C(207)	115.3(2)
C(208)-C(209)-C(210)	111.5(2)
C(208)-C(209)-C(215)	116.4(2)
C(210)-C(209)-C(215)	103.7(2)

C(208)-C(209)-H(209)	108.3
C(210)-C(209)-H(209)	108.3
C(215)-C(209)-H(209)	108.3
O(203)-C(210)-O(202)	121.5(3)
O(203)-C(210)-C(209)	128.4(3)
O(202)-C(210)-C(209)	110.1(2)
C(210)-O(202)-C(211)	111.3(2)
O(202)-C(211)-C(212)	110.8(2)
O(202)-C(211)-C(215)	105.3(2)
C(212)-C(211)-C(215)	106.4(2)
O(202)-C(211)-H(211)	111.4
C(212)-C(211)-H(211)	111.4
C(215)-C(211)-H(211)	111.4
C(211)-C(212)-C(213)	106.5(2)
C(211)-C(212)-H(21F)	110.4
C(213)-C(212)-H(21F)	110.4
C(211)-C(212)-H(21G)	110.4
C(213)-C(212)-H(21G)	110.4
H(21F)-C(212)-H(21G)	108.6
O(201)-C(213)-C(219)	106.9(2)
O(201)-C(213)-C(212)	110.2(2)
C(219)-C(213)-C(212)	112.9(2)
O(201)-C(213)-C(214)	105.9(2)
C(219)-C(213)-C(214)	114.8(2)
C(212)-C(213)-C(214)	105.9(2)
C(213)-C(219)-H(21H)	109.5
C(213)-C(219)-H(21I)	109.5
H(21H)-C(219)-H(21I)	109.5
C(213)-C(219)-H(21J)	109.5
H(21H)-C(219)-H(21J)	109.5
H(21I)-C(219)-H(21J)	109.5
C(202)-C(214)-C(215)	116.6(2)
C(202)-C(214)-C(213)	104.1(2)
C(215)-C(214)-C(213)	107.3(2)
C(202)-C(214)-H(214)	109.5

C(215)-C(214)-H(214)	109.5
C(213)-C(214)-H(214)	109.5
C(211)-C(215)-C(209)	103.4(2)
C(211)-C(215)-C(214)	106.6(2)
C(209)-C(215)-C(214)	115.6(2)
C(211)-C(215)-H(215)	110.3
C(209)-C(215)-H(215)	110.3
C(214)-C(215)-H(215)	110.3

Symmetry transformations used to generate equivalent atoms:

Table S6. Anisotropic displacement parameters (Å^{2}x 10^3) for V22241. The anisotropic displacement factor exponent takes the form: $-2p^2[h^2 a^{*2}U^{11} + ... + 2 h k a^* b^* U^{12}]$

	U^{11}	U^{22}	U^{33}	U^{23}	U^{13}	U^{12}
O(1)	16(1)	38(1)	28(1)	5(1)	2(1)	1(1)
C(1)	20(1)	29(1)	27(1)	2(1)	8(1)	3(1)
C(2)	17(1)	23(1)	24(1)	-3(1)	1(1)	2(1)
O(4)	73(2)	36(1)	21(1)	-1(1)	9(1)	7(1)
C(3)	20(1)	26(1)	23(1)	-1(1)	2(1)	3(1)
C(4)	46(2)	33(2)	23(2)	0(1)	3(1)	9(1)
O(5)	32(1)	32(1)	22(1)	-6(1)	-3(1)	-2(1)
C(5)	86(3)	38(2)	22(2)	6(1)	5(2)	24(2)
C(6)	63(2)	26(1)	31(2)	4(1)	16(2)	10(1)
C(16)	91(3)	26(2)	40(2)	9(1)	34(2)	15(2)
C(17)	78(3)	34(2)	66(3)	16(2)	29(2)	22(2)
C(18)	122(4)	28(2)	56(3)	-5(2)	12(3)	5(2)
C(7)	36(2)	24(1)	24(1)	0(1)	6(1)	7(1)
C(8)	20(1)	24(1)	23(1)	1(1)	4(1)	4(1)
C(9)	22(1)	21(1)	20(1)	-3(1)	4(1)	2(1)
C(10)	26(1)	23(1)	17(1)	-4(1)	0(1)	-2(1)
O(2)	22(1)	27(1)	25(1)	1(1)	-1(1)	-4(1)
O(3)	32(1)	28(1)	29(1)	-1(1)	0(1)	-11(1)
C(11)	21(1)	25(1)	24(1)	1(1)	2(1)	-3(1)
C(12)	22(1)	25(1)	30(1)	6(1)	1(1)	1(1)
C(13)	16(1)	24(1)	30(1)	1(1)	2(1)	1(1)
C(19)	36(2)	24(1)	36(2)	-3(1)	3(1)	7(1)
C(14)	13(1)	21(1)	26(1)	-2(1)	2(1)	-2(1)
C(15)	15(1)	24(1)	23(1)	1(1)	5(1)	0(1)
O(201)	15(1)	28(1)	34(1)	1(1)	1(1)	-2(1)
C(201)	15(1)	22(1)	29(1)	-4(1)	-1(1)	1(1)
C(202)	19(1)	24(1)	24(1)	-6(1)	1(1)	1(1)
O(204)	80(2)	31(1)	26(1)	-5(1)	-6(1)	-8(1)
C(203)	18(1)	27(1)	26(1)	-5(1)	3(1)	0(1)
C(204)	42(2)	32(2)	27(2)	-6(1)	4(1)	-1(1)

O(205)	28(1)	24(1)	25(1)	-7(1)	4(1)	2(1)
C(205)	75(3)	41(2)	25(2)	-6(1)	15(2)	-19(2)
C(206)	69(2)	30(2)	35(2)	-2(1)	13(2)	-14(2)
C(216)	91(9)	37(4)	30(4)	1(3)	11(5)	-22(5)
C(217)	93(5)	33(3)	40(5)	-2(3)	5(4)	-28(3)
C(218)	137(11)	47(5)	42(5)	12(4)	-9(6)	-33(6)
C(16B)	55(8)	27(6)	31(10)	-7(5)	19(7)	-6(5)
C(17B)	74(8)	23(5)	37(8)	3(5)	5(6)	-4(5)
C(18B)	64(7)	40(6)	46(11)	-2(6)	13(6)	-14(5)
C(207)	51(2)	30(2)	28(2)	-7(1)	14(1)	-16(1)
C(208)	22(1)	29(1)	27(1)	-5(1)	6(1)	-5(1)
C(209)	24(1)	26(1)	26(1)	-8(1)	5(1)	-7(1)
C(210)	26(1)	28(1)	23(1)	-10(1)	3(1)	-3(1)
O(202)	24(1)	28(1)	29(1)	-6(1)	7(1)	-4(1)
O(203)	35(1)	32(1)	33(1)	-6(1)	6(1)	6(1)
C(211)	24(1)	31(1)	24(1)	-2(1)	4(1)	-3(1)
C(212)	29(2)	29(1)	30(2)	1(1)	4(1)	0(1)
C(213)	17(1)	26(1)	30(1)	-2(1)	1(1)	1(1)
C(219)	31(2)	26(1)	35(2)	-2(1)	2(1)	4(1)
C(214)	13(1)	26(1)	28(1)	-3(1)	3(1)	2(1)
C(215)	14(1)	34(1)	23(1)	-3(1)	1(1)	-3(1)

Table S7. Hydrogen coordinates (x 10^4) and isotropic displacement parameters (Å^{2}x 10 ^{3}) for V22241.

	x	y	z	U(eq)
H(1A)	2639	6024	9321	30
H(1B)	3134	6730	9741	30
H(5O)	7220(80)	6480(20)	10400(20)	44
H(5A)	7560	4399	10747	59
H(5B)	9804	4590	10147	59
H(6)	4990	4252	9408	47
H(17A)	9729	2815	9838	70
H(17B)	10427	3540	10223	70
H(18A)	6176	2647	8896	103
H(18B)	5949	3212	8160	103
H(18C)	4068	3181	8950	103
H(7A)	9259	4451	8409	34
H(7B)	6510	4343	7988	34
H(9)	7784	5301	7161	25
H(11)	6153	6537	6143	28
H(12A)	5394	7489	6830	31
H(12B)	2680	7184	6857	31
H(19A)	3242	7993	8372	48
H(19B)	6172	8012	8317	48
H(19C)	4989	7715	9190	48
H(14)	8444	6953	8456	24
H(15)	9028	6316	7275	25
H(20A)	-2911	4014	4381	27
H(20B)	-3424	3505	3572	27
H(05O)	470(70)	3231(19)	2813(19)	38
H(20C)	1691	5144	1776	56
H(20D)	4115	5005	2416	56
H(206)	-267	5613	3006	53
H(21A)	4833	6962	3000	67

H(21B)	5195	6371	3738	67
H(21C)	1823	6910	1799	114
H(21D)	-453	6450	1987	114
H(21E)	1719	6194	1398	114
H(17C)	762	7033	1986	54
H(17D)	-1387	6504	2191	54
H(18D)	5558	6145	2350	75
H(18E)	5239	6381	3366	75
H(18F)	4839	6877	2542	75
H(20E)	4252	5450	4069	43
H(20F)	1709	5710	4422	43
H(209)	2872	4896	5578	30
H(211)	1333	3899	6941	31
H(21F)	-2168	3099	6461	35
H(21G)	602	2838	6645	35
H(21H)	-1373	1976	5350	46
H(21I)	1543	2086	5461	46
H(21J)	140	2127	4480	46
H(214)	3061	3121	4755	27
H(215)	3952	3868	5802	29

Table S8. Torsion angles [°] for V22241.

C(13)-O(1)-C(1)-C(2)	-31.1(3)
O(1)-C(1)-C(2)-O(5)	121.4(2)
O(1)-C(1)-C(2)-C(3)	-115.2(2)
O(1)-C(1)-C(2)-C(14)	8.2(3)
O(5)-C(2)-C(3)-C(8)	-145.2(3)
C(14)-C(2)-C(3)-C(8)	-24.7(4)
C(1)-C(2)-C(3)-C(8)	91.5(3)
O(5)-C(2)-C(3)-C(4)	41.3(3)
C(14)-C(2)-C(3)-C(4)	161.9(2)
C(1)-C(2)-C(3)-C(4)	-82.0(3)
C(8)-C(3)-C(4)-O(4)	-167.8(3)
C(2)-C(3)-C(4)-O(4)	5.9(5)
C(8)-C(3)-C(4)-C(5)	13.0(5)
C(2)-C(3)-C(4)-C(5)	-173.4(3)
O(4)-C(4)-C(5)-C(6)	138.4(4)
C(3)-C(4)-C(5)-C(6)	-42.3(5)
C(4)-C(5)-C(6)-C(16)	-176.3(3)
C(4)-C(5)-C(6)-C(7)	59.0(4)
C(5)-C(6)-C(16)-C(17)	-18.4(5)
C(7)-C(6)-C(16)-C(17)	105.1(4)
C(5)-C(6)-C(16)-C(18)	161.7(4)
C(7)-C(6)-C(16)-C(18)	-74.9(5)
C(16)-C(6)-C(7)-C(8)	-175.7(3)
C(5)-C(6)-C(7)-C(8)	-48.4(4)
C(4)-C(3)-C(8)-C(9)	175.2(3)
C(2)-C(3)-C(8)-C(9)	1.8(4)
C(4)-C(3)-C(8)-C(7)	-2.0(4)
C(2)-C(3)-C(8)-C(7)	-175.3(3)
C(6)-C(7)-C(8)-C(3)	21.0(4)
C(6)-C(7)-C(8)-C(9)	-156.4(3)
C(3)-C(8)-C(9)-C(15)	26.5(4)
C(7)-C(8)-C(9)-C(15)	-156.1(2)
C(3)-C(8)-C(9)-C(10)	-93.3(3)

C(7)-C(8)-C(9)-C(10)	84.1(3)
C(8)-C(9)-C(10)-O(3)	-35.0(4)
C(15)-C(9)-C(10)-O(3)	-162.5(3)
C(8)-C(9)-C(10)-O(2)	146.4(2)
C(15)-C(9)-C(10)-O(2)	18.9(3)
O(3)-C(10)-O(2)-C(11)	174.3(2)
C(9)-C(10)-O(2)-C(11)	-7.0(3)
C(10)-O(2)-C(11)-C(12)	-122.5(2)
C(10)-O(2)-C(11)-C(15)	-7.7(3)
O(2)-C(11)-C(12)-C(13)	87.4(3)
C(15)-C(11)-C(12)-C(13)	-27.5(3)
C(1)-O(1)-C(13)-C(12)	152.7(2)
C(1)-O(1)-C(13)-C(19)	-82.4(3)
C(1)-O(1)-C(13)-C(14)	40.8(3)
C(11)-C(12)-C(13)-O(1)	-78.7(3)
C(11)-C(12)-C(13)-C(19)	157.9(2)
C(11)-C(12)-C(13)-C(14)	30.3(3)
O(1)-C(13)-C(14)-C(2)	-33.9(2)
C(12)-C(13)-C(14)-C(2)	-146.4(2)
C(19)-C(13)-C(14)-C(2)	86.8(3)
O(1)-C(13)-C(14)-C(15)	91.6(2)
C(12)-C(13)-C(14)-C(15)	-21.0(3)
C(19)-C(13)-C(14)-C(15)	-147.8(2)
O(5)-C(2)-C(14)-C(13)	-100.7(2)
C(3)-C(2)-C(14)-C(13)	136.3(2)
C(1)-C(2)-C(14)-C(13)	15.8(3)
O(5)-C(2)-C(14)-C(15)	142.1(2)
C(3)-C(2)-C(14)-C(15)	19.1(3)
C(1)-C(2)-C(14)-C(15)	-101.5(2)
C(8)-C(9)-C(15)-C(11)	-145.6(2)
C(10)-C(9)-C(15)-C(11)	-21.9(3)
C(8)-C(9)-C(15)-C(14)	-29.4(3)
C(10)-C(9)-C(15)-C(14)	94.3(2)
O(2)-C(11)-C(15)-C(9)	18.7(3)
C(12)-C(11)-C(15)-C(9)	136.6(2)

O(2)-C(11)-C(15)-C(14)	-103.5(2)
C(12)-C(11)-C(15)-C(14)	14.4(3)
C(13)-C(14)-C(15)-C(9)	-110.1(2)
C(2)-C(14)-C(15)-C(9)	6.6(3)
C(13)-C(14)-C(15)-C(11)	4.0(3)
C(2)-C(14)-C(15)-C(11)	120.7(2)
C(213)-O(201)-C(201)-C(202)	20.2(3)
O(201)-C(201)-C(202)-O(205)	80.4(3)
O(201)-C(201)-C(202)-C(203)	-153.6(2)
O(201)-C(201)-C(202)-C(214)	-31.0(3)
O(205)-C(202)-C(203)-C(208)	-154.0(3)
C(214)-C(202)-C(203)-C(208)	-34.2(4)
C(201)-C(202)-C(203)-C(208)	81.4(3)
O(205)-C(202)-C(203)-C(204)	29.6(4)
C(214)-C(202)-C(203)-C(204)	149.4(3)
C(201)-C(202)-C(203)-C(204)	-95.0(3)
C(208)-C(203)-C(204)-O(204)	-171.4(3)
C(202)-C(203)-C(204)-O(204)	5.0(5)
C(208)-C(203)-C(204)-C(205)	8.8(5)
C(202)-C(203)-C(204)-C(205)	-174.8(3)
O(204)-C(204)-C(205)-C(206)	142.0(4)
C(203)-C(204)-C(205)-C(206)	-38.2(5)
C(204)-C(205)-C(206)-C(207)	57.7(5)
C(204)-C(205)-C(206)-C(216)	179.6(7)
C(204)-C(205)-C(206)-C(16B)	-163.7(9)
C(207)-C(206)-C(216)-C(217)	9(2)
C(205)-C(206)-C(216)-C(217)	-112(2)
C(207)-C(206)-C(216)-C(218)	-165.5(10)
C(205)-C(206)-C(216)-C(218)	73.3(13)
C(207)-C(206)-C(16B)-C(17B)	-128(3)
C(205)-C(206)-C(16B)-C(17B)	99(3)
C(207)-C(206)-C(16B)-C(18B)	55(2)
C(205)-C(206)-C(16B)-C(18B)	-78(2)
C(205)-C(206)-C(207)-C(208)	-48.7(4)
C(216)-C(206)-C(207)-C(208)	-170.3(8)

C(16B)-C(206)-C(207)-C(208)	177.4(11)
C(204)-C(203)-C(208)-C(209)	178.9(3)
C(202)-C(203)-C(208)-C(209)	2.5(4)
C(204)-C(203)-C(208)-C(207)	0.5(5)
C(202)-C(203)-C(208)-C(207)	-175.9(3)
C(206)-C(207)-C(208)-C(203)	20.7(5)
C(206)-C(207)-C(208)-C(209)	-157.8(3)
C(203)-C(208)-C(209)-C(210)	-89.1(3)
C(207)-C(208)-C(209)-C(210)	89.5(3)
C(203)-C(208)-C(209)-C(215)	29.6(4)
C(207)-C(208)-C(209)-C(215)	-151.8(3)
C(208)-C(209)-C(210)-O(203)	-42.0(4)
C(215)-C(209)-C(210)-O(203)	-168.1(3)
C(208)-C(209)-C(210)-O(202)	136.9(2)
C(215)-C(209)-C(210)-O(202)	10.8(3)
O(203)-C(210)-O(202)-C(211)	-175.8(2)
C(209)-C(210)-O(202)-C(211)	5.2(3)
C(210)-O(202)-C(211)-C(212)	-133.8(2)
C(210)-O(202)-C(211)-C(215)	-19.2(3)
O(202)-C(211)-C(212)-C(213)	86.5(3)
C(215)-C(211)-C(212)-C(213)	-27.5(3)
C(201)-O(201)-C(213)-C(219)	-123.5(2)
C(201)-O(201)-C(213)-C(212)	113.4(2)
C(201)-O(201)-C(213)-C(214)	-0.7(3)
C(211)-C(212)-C(213)-O(201)	-89.9(3)
C(211)-C(212)-C(213)-C(219)	150.6(2)
C(211)-C(212)-C(213)-C(214)	24.2(3)
O(205)-C(202)-C(214)-C(215)	156.8(2)
C(203)-C(202)-C(214)-C(215)	32.9(3)
C(201)-C(202)-C(214)-C(215)	-88.3(3)
O(205)-C(202)-C(214)-C(213)	-85.2(2)
C(203)-C(202)-C(214)-C(213)	150.8(2)
C(201)-C(202)-C(214)-C(213)	29.7(3)
O(201)-C(213)-C(214)-C(202)	-18.9(3)
C(219)-C(213)-C(214)-C(202)	98.8(3)

C(212)-C(213)-C(214)-C(202)	-136.0(2)
O(201)-C(213)-C(214)-C(215)	105.3(2)
C(219)-C(213)-C(214)-C(215)	-137.0(2)
C(212)-C(213)-C(214)-C(215)	-11.8(3)
O(202)-C(211)-C(215)-C(209)	24.6(3)
C(212)-C(211)-C(215)-C(209)	142.2(2)
O(202)-C(211)-C(215)-C(214)	-97.7(2)
C(212)-C(211)-C(215)-C(214)	19.9(3)
C(208)-C(209)-C(215)-C(211)	-144.1(2)
C(210)-C(209)-C(215)-C(211)	-21.2(3)
C(208)-C(209)-C(215)-C(214)	-27.9(3)
C(210)-C(209)-C(215)-C(214)	94.9(3)
C(202)-C(214)-C(215)-C(211)	111.3(3)
C(213)-C(214)-C(215)-C(211)	-4.9(3)
C(202)-C(214)-C(215)-C(209)	-3.0(3)
C(213)-C(214)-C(215)-C(209)	-119.2(3)

Symmetry transformations used to generate equivalent atoms:

Table S9. Hydrogen bonds for V22241 [Å and °].

D-H...A	d(D-H)	d(H...A)	d(D...A)	<(DHA)
O(5)-H(5O)...O(4)	0.83(3)	2.04(3)	2.710(3)	138(4)
C(5)-H(5A)...O(204)#1	0.99	2.59	3.299(4)	128.6
C(9)-H(9)...O(202)#2	1.00	2.65	3.542(3)	149.2
C(9)-H(9)...O(203)#2	1.00	2.64	3.406(3)	133.5
C(12)-H(12A)...O(205)#3	0.99	2.43	3.342(3)	153.7
C(14)-H(14)...O(1)#2	1.00	2.37	3.119(3)	130.8
C(15)-H(15)...O(1)#2	1.00	2.65	3.288(3)	121.6
O(205)-H(05O)...O(204)	0.82(3)	1.95(3)	2.640(3)	141(4)
C(209)-H(209)...O(3)	1.00	2.60	3.206(3)	119.2
C(211)-H(211)...O(3)	1.00	2.56	3.146(3)	117.5
C(214)-H(214)...O(201)#2	1.00	2.29	3.178(3)	147.0
C(215)-H(215)...O(202)#2	1.00	2.61	3.542(3)	154.9

Symmetry transformations used to generate equivalent atoms:

#1 x+1,y,z+1 #2 x+1,y,z #3 -x+1,y+1/2,-z+1

Figure S4: X-ray coordinate of compound **17**.

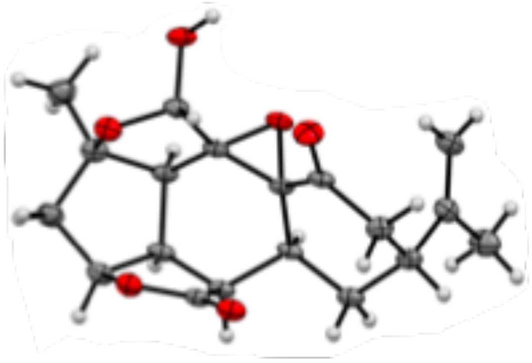

Table S10. Crystal data and structure refinement for V22369.

Identification code	V22369	
Empirical formula	C19 H22 O6	
Formula weight	346.36	
Temperature	100(2) K	
Wavelength	1.54178 Å	
Crystal system	Orthorhombic	
Space group	$P2_12_12_1$	
Unit cell dimensions	a = 6.0176(4) Å	a= 90°.
	b = 12.9933(17) Å	b= 90°.
	c = 20.752(2) Å	g = 90°.
Volume	1622.6(3) Å^3	
Z	4	
Density (calculated)	1.418 Mg/m^3	
Absorption coefficient	0.874 mm^{-1}	
F(000)	736	
Crystal size	0.300 x 0.300 x 0.050 mm^3	
Theta range for data collection	4.014 to 74.419°.	
Index ranges	-7<=h<=7, -14<=k<=16, -25<=l<=22	
Reflections collected	17915	
Independent reflections	3315 [R(int) = 0.0964]	
Completeness to theta = 67.679°	100.0 %	
Absorption correction	Semi-empirical from equivalents	
Max. and min. transmission	0.7538 and 0.4675	
Refinement method	Full-matrix least-squares on F^2	

Data / restraints / parameters	3315 / 1 / 231
Goodness-of-fit on F^2	1.068
Final R indices [I>2sigma(I)]	R1 = 0.0516, wR2 = 0.1264
R indices (all data)	R1 = 0.0594, wR2 = 0.1320
Absolute structure parameter	0.02(14)
Extinction coefficient	n/a
Largest diff. peak and hole	0.457 and -0.323 e.Å^{-3}

Table S11. Atomic coordinates (x 10^4) and equivalent isotropic displacement parameters ($Å^2$x 10^3) for V22369. U(eq) is defined as one third of the trace of the orthogonalized U^{ij} tensor.

	x	y	z	U(eq)
O(1)	2960(4)	4788(2)	8897(1)	25(1)
O(2)	1892(4)	6270(2)	8370(1)	27(1)
C(1)	2981(6)	5314(2)	8307(2)	21(1)
C(2)	1690(5)	4607(2)	7847(2)	19(1)
O(3)	633(4)	5051(2)	7295(1)	23(1)
C(3)	2389(5)	4301(2)	7184(2)	19(1)
C(4)	4363(5)	4811(2)	6862(2)	20(1)
O(4)	5136(5)	5596(2)	7076(1)	30(1)
C(5)	5223(5)	4335(2)	6245(2)	23(1)
C(6)	3506(6)	3683(2)	5891(2)	22(1)
C(16)	1575(6)	4278(3)	5608(2)	23(1)
C(17)	1223(6)	5275(3)	5695(2)	29(1)
C(18)	80(7)	3656(3)	5173(2)	33(1)
C(7)	2766(6)	2818(2)	6349(2)	22(1)
C(8)	1674(5)	3218(2)	6976(2)	18(1)
C(9)	2147(5)	2494(2)	7547(2)	18(1)
C(10)	4520(5)	2638(2)	7789(2)	19(1)
O(5)	4575(4)	2656(2)	8443(1)	23(1)
O(6)	6185(4)	2784(2)	7481(1)	23(1)
C(11)	2371(6)	2430(2)	8699(2)	24(1)
C(12)	1785(6)	3126(3)	9261(2)	27(1)
C(13)	1006(6)	4139(2)	8956(2)	24(1)
C(19)	-769(7)	4696(3)	9340(2)	31(1)
C(14)	314(5)	3881(2)	8254(2)	20(1)
C(15)	800(5)	2712(2)	8150(2)	20(1)

Table S12. Bond lengths [Å] and angles [°] for V22369.

O(1)-C(1)	1.403(4)
O(1)-C(13)	1.452(4)
O(2)-C(1)	1.410(4)
O(2)-H(2O)	0.85(3)
C(1)-C(2)	1.536(5)
C(1)-H(1)	1.0000
C(2)-O(3)	1.432(4)
C(2)-C(3)	1.492(5)
C(2)-C(14)	1.513(4)
O(3)-C(3)	1.456(4)
C(3)-C(4)	1.516(4)
C(3)-C(8)	1.533(4)
C(4)-O(4)	1.206(4)
C(4)-C(5)	1.512(5)
C(5)-C(6)	1.525(5)
C(5)-H(5A)	0.9900
C(5)-H(5B)	0.9900
C(6)-C(16)	1.514(5)
C(6)-C(7)	1.538(4)
C(6)-H(6)	1.0000
C(16)-C(17)	1.325(5)
C(16)-C(18)	1.510(5)
C(17)-H(17A)	0.9500
C(17)-H(17B)	0.9500
C(18)-H(18A)	0.9800
C(18)-H(18B)	0.9800
C(18)-H(18C)	0.9800
C(7)-C(8)	1.547(5)
C(7)-H(7A)	0.9900
C(7)-H(7B)	0.9900
C(8)-C(9)	1.540(4)
C(8)-H(8)	1.0000
C(9)-C(15)	1.518(5)

C(9)-C(10)	1.525(4)
C(9)-H(9)	1.0000
C(10)-O(6)	1.203(4)
C(10)-O(5)	1.358(4)
O(5)-C(11)	1.458(4)
C(11)-C(12)	1.519(5)
C(11)-C(15)	1.524(5)
C(11)-H(11)	1.0000
C(12)-C(13)	1.534(5)
C(12)-H(12A)	0.9900
C(12)-H(12B)	0.9900
C(13)-C(19)	1.516(5)
C(13)-C(14)	1.552(5)
C(19)-H(19A)	0.9800
C(19)-H(19B)	0.9800
C(19)-H(19C)	0.9800
C(14)-C(15)	1.563(4)
C(14)-H(14)	1.0000
C(15)-H(15)	1.0000

C(1)-O(1)-C(13)	111.3(3)
C(1)-O(2)-H(2O)	116(4)
O(1)-C(1)-O(2)	110.1(3)
O(1)-C(1)-C(2)	104.3(2)
O(2)-C(1)-C(2)	110.5(3)
O(1)-C(1)-H(1)	110.6
O(2)-C(1)-H(1)	110.6
C(2)-C(1)-H(1)	110.6
O(3)-C(2)-C(3)	59.7(2)
O(3)-C(2)-C(14)	117.1(3)
C(3)-C(2)-C(14)	120.2(3)
O(3)-C(2)-C(1)	118.7(2)
C(3)-C(2)-C(1)	126.1(3)
C(14)-C(2)-C(1)	107.6(3)
C(2)-O(3)-C(3)	62.2(2)

O(3)-C(3)-C(2)	58.11(19)
O(3)-C(3)-C(4)	110.2(2)
C(2)-C(3)-C(4)	120.8(3)
O(3)-C(3)-C(8)	117.1(3)
C(2)-C(3)-C(8)	115.2(3)
C(4)-C(3)-C(8)	119.8(3)
O(4)-C(4)-C(5)	121.8(3)
O(4)-C(4)-C(3)	120.6(3)
C(5)-C(4)-C(3)	117.6(3)
C(4)-C(5)-C(6)	113.8(3)
C(4)-C(5)-H(5A)	108.8
C(6)-C(5)-H(5A)	108.8
C(4)-C(5)-H(5B)	108.8
C(6)-C(5)-H(5B)	108.8
H(5A)-C(5)-H(5B)	107.7
C(16)-C(6)-C(5)	115.0(3)
C(16)-C(6)-C(7)	113.0(3)
C(5)-C(6)-C(7)	107.7(3)
C(16)-C(6)-H(6)	106.9
C(5)-C(6)-H(6)	106.9
C(7)-C(6)-H(6)	106.9
C(17)-C(16)-C(18)	120.7(3)
C(17)-C(16)-C(6)	124.7(3)
C(18)-C(16)-C(6)	114.6(3)
C(16)-C(17)-H(17A)	120.0
C(16)-C(17)-H(17B)	120.0
H(17A)-C(17)-H(17B)	120.0
C(16)-C(18)-H(18A)	109.5
C(16)-C(18)-H(18B)	109.5
H(18A)-C(18)-H(18B)	109.5
C(16)-C(18)-H(18C)	109.5
H(18A)-C(18)-H(18C)	109.5
H(18B)-C(18)-H(18C)	109.5
C(6)-C(7)-C(8)	113.4(2)
C(6)-C(7)-H(7A)	108.9

C(8)-C(7)-H(7A)	108.9
C(6)-C(7)-H(7B)	108.9
C(8)-C(7)-H(7B)	108.9
H(7A)-C(7)-H(7B)	107.7
C(3)-C(8)-C(9)	107.0(2)
C(3)-C(8)-C(7)	115.2(3)
C(9)-C(8)-C(7)	111.3(2)
C(3)-C(8)-H(8)	107.7
C(9)-C(8)-H(8)	107.7
C(7)-C(8)-H(8)	107.7
C(15)-C(9)-C(10)	101.9(3)
C(15)-C(9)-C(8)	115.0(2)
C(10)-C(9)-C(8)	110.6(3)
C(15)-C(9)-H(9)	109.7
C(10)-C(9)-H(9)	109.7
C(8)-C(9)-H(9)	109.7
O(6)-C(10)-O(5)	120.5(3)
O(6)-C(10)-C(9)	128.6(3)
O(5)-C(10)-C(9)	110.7(3)
C(10)-O(5)-C(11)	109.7(3)
O(5)-C(11)-C(12)	111.7(3)
O(5)-C(11)-C(15)	104.2(3)
C(12)-C(11)-C(15)	106.7(3)
O(5)-C(11)-H(11)	111.3
C(12)-C(11)-H(11)	111.3
C(15)-C(11)-H(11)	111.3
C(11)-C(12)-C(13)	105.3(3)
C(11)-C(12)-H(12A)	110.7
C(13)-C(12)-H(12A)	110.7
C(11)-C(12)-H(12B)	110.7
C(13)-C(12)-H(12B)	110.7
H(12A)-C(12)-H(12B)	108.8
O(1)-C(13)-C(19)	109.7(3)
O(1)-C(13)-C(12)	106.6(3)
C(19)-C(13)-C(12)	114.1(3)

O(1)-C(13)-C(14)	105.3(3)
C(19)-C(13)-C(14)	114.0(3)
C(12)-C(13)-C(14)	106.5(3)
C(13)-C(19)-H(19A)	109.5
C(13)-C(19)-H(19B)	109.5
H(19A)-C(19)-H(19B)	109.5
C(13)-C(19)-H(19C)	109.5
H(19A)-C(19)-H(19C)	109.5
H(19B)-C(19)-H(19C)	109.5
C(2)-C(14)-C(13)	104.1(3)
C(2)-C(14)-C(15)	115.3(3)
C(13)-C(14)-C(15)	106.8(3)
C(2)-C(14)-H(14)	110.1
C(13)-C(14)-H(14)	110.1
C(15)-C(14)-H(14)	110.1
C(9)-C(15)-C(11)	103.8(3)
C(9)-C(15)-C(14)	113.3(3)
C(11)-C(15)-C(14)	104.3(3)
C(9)-C(15)-H(15)	111.6
C(11)-C(15)-H(15)	111.6
C(14)-C(15)-H(15)	111.6

Symmetry transformations used to generate equivalent atoms:

Table S13. Anisotropic displacement parameters ($Å^2$x 10^3) for V22369. The anisotropic displacement factor exponent takes the form: $-2p^2[h^2 a^{*2}U^{11} + ... + 2 h k a^* b^* U^{12}]$

	U^{11}	U^{22}	U^{33}	U^{23}	U^{13}	U^{12}
O(1)	22(1)	23(1)	31(1)	0(1)	-3(1)	-1(1)
O(2)	29(1)	15(1)	37(1)	-2(1)	5(1)	1(1)
C(1)	20(2)	16(1)	28(2)	1(1)	2(1)	-1(1)
C(2)	14(1)	14(1)	28(2)	0(1)	-2(1)	2(1)
O(3)	21(1)	15(1)	31(1)	2(1)	-2(1)	5(1)
C(3)	16(1)	14(1)	26(2)	-1(1)	-1(1)	4(1)
C(4)	19(1)	11(1)	30(2)	0(1)	0(1)	1(1)
O(4)	33(1)	20(1)	37(1)	-2(1)	4(1)	-11(1)
C(5)	17(2)	21(1)	30(2)	-2(1)	2(1)	-2(1)
C(6)	21(2)	17(1)	28(2)	-2(1)	1(1)	2(1)
C(16)	20(2)	23(2)	27(2)	3(1)	0(1)	0(1)
C(17)	28(2)	23(2)	35(2)	3(1)	-1(2)	5(1)
C(18)	28(2)	31(2)	41(2)	-2(2)	-10(2)	1(2)
C(7)	20(2)	15(1)	30(2)	-3(1)	-2(1)	1(1)
C(8)	13(1)	12(1)	29(2)	1(1)	-4(1)	0(1)
C(9)	13(1)	11(1)	30(2)	0(1)	-2(1)	0(1)
C(10)	18(2)	11(1)	29(2)	0(1)	-4(1)	1(1)
O(5)	16(1)	25(1)	29(1)	1(1)	-4(1)	2(1)
O(6)	14(1)	21(1)	35(1)	0(1)	-2(1)	1(1)
C(11)	20(2)	19(1)	32(2)	4(1)	1(1)	2(1)
C(12)	24(2)	27(2)	30(2)	2(1)	0(1)	5(1)
C(13)	17(2)	23(2)	32(2)	-1(1)	-1(1)	-1(1)
C(19)	29(2)	30(2)	34(2)	-1(1)	4(2)	4(2)
C(14)	14(1)	17(1)	29(2)	-1(1)	0(1)	2(1)
C(15)	14(1)	15(1)	32(2)	2(1)	-1(1)	0(1)

Table S14. Hydrogen coordinates (x 10^4) and isotropic displacement parameters ($Å^2$x 10^3) for V22369.

	x	y	z	U(eq)
H(2O)	2370(80)	6750(30)	8130(20)	40
H(1)	4540	5413	8152	26
H(5A)	6525	3900	6348	27
H(5B)	5732	4892	5955	27
H(6)	4294	3346	5523	26
H(17A)	18	5605	5483	34
H(17B)	2174	5660	5970	34
H(18A)	-1046	4107	4981	50
H(18B)	-653	3115	5424	50
H(18C)	968	3340	4830	50
H(7A)	4076	2395	6463	26
H(7B)	1696	2369	6121	26
H(8)	30	3230	6906	22
H(9)	1920	1763	7411	22
H(11)	2228	1687	8818	28
H(12A)	3099	3242	9539	32
H(12B)	587	2817	9525	32
H(19A)	-202	4850	9772	46
H(19B)	-2089	4258	9375	46
H(19C)	-1163	5338	9121	46
H(14)	-1304	4024	8191	24
H(15)	-594	2293	8164	24

Table S15. Torsion angles [°] for V22369.

C(13)-O(1)-C(1)-O(2)	90.2(3)
C(13)-O(1)-C(1)-C(2)	-28.4(3)
O(1)-C(1)-C(2)-O(3)	155.4(3)
O(2)-C(1)-C(2)-O(3)	37.1(4)
O(1)-C(1)-C(2)-C(3)	-132.8(3)
O(2)-C(1)-C(2)-C(3)	108.9(3)
O(1)-C(1)-C(2)-C(14)	19.5(3)
O(2)-C(1)-C(2)-C(14)	-98.8(3)
C(14)-C(2)-O(3)-C(3)	-110.9(3)
C(1)-C(2)-O(3)-C(3)	117.3(3)
C(2)-O(3)-C(3)-C(4)	-114.4(3)
C(2)-O(3)-C(3)-C(8)	104.2(3)
C(14)-C(2)-C(3)-O(3)	105.6(3)
C(1)-C(2)-C(3)-O(3)	-105.2(3)
O(3)-C(2)-C(3)-C(4)	95.9(3)
C(14)-C(2)-C(3)-C(4)	-158.5(3)
C(1)-C(2)-C(3)-C(4)	-9.3(4)
O(3)-C(2)-C(3)-C(8)	-107.4(3)
C(14)-C(2)-C(3)-C(8)	-1.8(4)
C(1)-C(2)-C(3)-C(8)	147.3(3)
O(3)-C(3)-C(4)-O(4)	50.0(4)
C(2)-C(3)-C(4)-O(4)	-14.2(5)
C(8)-C(3)-C(4)-O(4)	-169.8(3)
O(3)-C(3)-C(4)-C(5)	-126.5(3)
C(2)-C(3)-C(4)-C(5)	169.3(3)
C(8)-C(3)-C(4)-C(5)	13.7(4)
O(4)-C(4)-C(5)-C(6)	-153.6(3)
C(3)-C(4)-C(5)-C(6)	22.9(4)
C(4)-C(5)-C(6)-C(16)	67.8(4)
C(4)-C(5)-C(6)-C(7)	-59.2(3)
C(5)-C(6)-C(16)-C(17)	-5.8(5)
C(7)-C(6)-C(16)-C(17)	118.4(4)
C(5)-C(6)-C(16)-C(18)	171.0(3)

C(7)-C(6)-C(16)-C(18)	-64.8(4)
C(16)-C(6)-C(7)-C(8)	-67.3(3)
C(5)-C(6)-C(7)-C(8)	60.9(3)
O(3)-C(3)-C(8)-C(9)	-109.9(3)
C(2)-C(3)-C(8)-C(9)	-44.4(3)
C(4)-C(3)-C(8)-C(9)	112.5(3)
O(3)-C(3)-C(8)-C(7)	125.8(3)
C(2)-C(3)-C(8)-C(7)	-168.7(3)
C(4)-C(3)-C(8)-C(7)	-11.8(4)
C(6)-C(7)-C(8)-C(3)	-25.9(4)
C(6)-C(7)-C(8)-C(9)	-147.9(3)
C(3)-C(8)-C(9)-C(15)	64.2(3)
C(7)-C(8)-C(9)-C(15)	-169.1(2)
C(3)-C(8)-C(9)-C(10)	-50.4(3)
C(7)-C(8)-C(9)-C(10)	76.2(3)
C(15)-C(9)-C(10)-O(6)	-161.4(3)
C(8)-C(9)-C(10)-O(6)	-38.7(4)
C(15)-C(9)-C(10)-O(5)	14.2(3)
C(8)-C(9)-C(10)-O(5)	136.8(3)
O(6)-C(10)-O(5)-C(11)	-178.4(3)
C(9)-C(10)-O(5)-C(11)	5.6(3)
C(10)-O(5)-C(11)-C(12)	-137.9(3)
C(10)-O(5)-C(11)-C(15)	-23.1(3)
O(5)-C(11)-C(12)-C(13)	80.8(3)
C(15)-C(11)-C(12)-C(13)	-32.4(3)
C(1)-O(1)-C(13)-C(19)	-97.2(3)
C(1)-O(1)-C(13)-C(12)	138.8(3)
C(1)-O(1)-C(13)-C(14)	25.9(3)
C(11)-C(12)-C(13)-O(1)	-91.1(3)
C(11)-C(12)-C(13)-C(19)	147.7(3)
C(11)-C(12)-C(13)-C(14)	20.9(3)
O(3)-C(2)-C(14)-C(13)	-141.2(3)
C(3)-C(2)-C(14)-C(13)	149.8(3)
C(1)-C(2)-C(14)-C(13)	-4.4(3)
O(3)-C(2)-C(14)-C(15)	102.3(3)

C(3)-C(2)-C(14)-C(15)	33.2(4)
C(1)-C(2)-C(14)-C(15)	-121.0(3)
O(1)-C(13)-C(14)-C(2)	-11.7(3)
C(19)-C(13)-C(14)-C(2)	108.6(3)
C(12)-C(13)-C(14)-C(2)	-124.6(3)
O(1)-C(13)-C(14)-C(15)	110.7(3)
C(19)-C(13)-C(14)-C(15)	-129.0(3)
C(12)-C(13)-C(14)-C(15)	-2.3(3)
C(10)-C(9)-C(15)-C(11)	-27.0(3)
C(8)-C(9)-C(15)-C(11)	-146.6(3)
C(10)-C(9)-C(15)-C(14)	85.5(3)
C(8)-C(9)-C(15)-C(14)	-34.1(4)
O(5)-C(11)-C(15)-C(9)	31.1(3)
C(12)-C(11)-C(15)-C(9)	149.4(3)
O(5)-C(11)-C(15)-C(14)	-87.8(3)
C(12)-C(11)-C(15)-C(14)	30.6(3)
C(2)-C(14)-C(15)-C(9)	-14.2(4)
C(13)-C(14)-C(15)-C(9)	-129.3(3)
C(2)-C(14)-C(15)-C(11)	98.0(3)
C(13)-C(14)-C(15)-C(11)	-17.1(3)

Table S16. Hydrogen bonds for V22369 [Å and °].

D-H...A	d(D-H)	d(H...A)	d(D...A)	<(DHA)
O(2)-H(2O)...O(6)#1	0.85(3)	2.04(3)	2.887(3)	173(5)
C(1)-H(1)...O(4)	1.00	2.27	2.887(4)	118.5
C(9)-H(9)...O(4)#2	1.00	2.56	3.060(4)	110.5
C(14)-H(14)...O(6)#3	1.00	2.65	3.283(4)	121.0
C(15)-H(15)...O(6)#3	1.00	2.48	3.106(4)	119.9

Symmetry transformations used to generate equivalent atoms:

#1 -x+1,y+1/2,-z+3/2 #2 -x+1,y-1/2,-z+3/2 #3 x-1,y,z

Figure S5: X-ray coordinate of compound **1**.

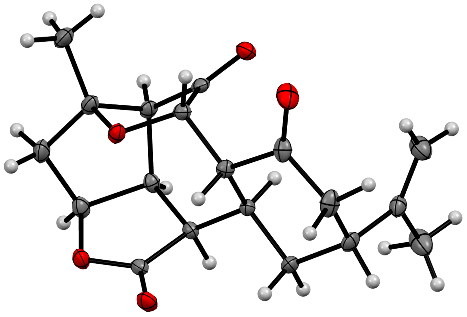

Table S17. Crystal data and structure refinement for V23078.

Identification code	V23078	
Empirical formula	C19 H22 O5	
Formula weight	330.36	
Temperature	100(2) K	
Wavelength	1.54178 Å	
Crystal system	Orthorhombic	
Space group	$P2_12_12_1$	
Unit cell dimensions	a = 10.5762(8) Å	a= 90°.
	b = 10.7831(11) Å	b= 90°.
	c = 13.8667(15) Å	g = 90°.
Volume	1581.4(3) $Å^3$	
Z	4	
Density (calculated)	1.388 Mg/m^3	
Absorption coefficient	0.820 mm^{-1}	
F(000)	704	
Crystal size	0.300 x 0.150 x 0.100 mm^3	
Theta range for data collection	5.196 to 74.559°.	
Index ranges	-13<=h<=13, -13<=k<=13, -17<=l<=17	
Reflections collected	25211	
Independent reflections	3240 [R(int) = 0.0504]	
Completeness to theta = 67.679°	100.0 %	
Absorption correction	Semi-empirical from equivalents	
Max. and min. transmission	0.7538 and 0.6138	
Refinement method	Full-matrix least-squares on F^2	
Data / restraints / parameters	3240 / 0 / 219	
Goodness-of-fit on F^2	1.045	

Final R indices [I>2sigma(I)]	R1 = 0.0306, wR2 = 0.0791
R indices (all data)	R1 = 0.0324, wR2 = 0.0803
Absolute structure parameter	-0.02(5)
Extinction coefficient	n/a
Largest diff. peak and hole	0.756 and -0.188 e.Å^{-3}

Table S18. Atomic coordinates (x 10^4) and equivalent isotropic displacement parameters ($Å^2$x 10^3) for V23078. U(eq) is defined as one third of the trace of the orthogonalized U^{ij} tensor.

	x	y	z	U(eq)
C(1)	5052(2)	2908(2)	4104(1)	15(1)
O(1)	4757(1)	1706(1)	4235(1)	18(1)
O(2)	4325(1)	3602(1)	3709(1)	20(1)
C(2)	6325(2)	3210(2)	4553(1)	13(1)
C(3)	6950(2)	1924(2)	4738(1)	15(1)
C(4)	5836(2)	997(2)	4568(1)	19(1)
C(5)	6253(2)	118(2)	3766(2)	22(1)
C(6)	7333(2)	769(2)	3257(2)	18(1)
C(16)	8121(2)	-69(2)	2615(2)	23(1)
O(3)	6826(1)	1824(1)	2717(1)	16(1)
C(7)	8042(2)	1445(2)	4071(1)	16(1)
C(8)	8704(2)	2428(2)	3493(1)	14(1)
O(4)	9762(1)	2839(1)	3584(1)	18(1)
C(9)	7777(2)	2781(2)	2698(1)	14(1)
C(10)	7158(2)	4030(2)	2932(1)	14(1)
C(11)	7806(2)	5120(2)	2425(1)	18(1)
O(5)	8613(2)	4967(2)	1815(1)	27(1)
C(12)	7301(2)	6387(2)	2684(2)	23(1)
C(13)	7166(2)	6591(2)	3779(1)	18(1)
C(17)	8417(2)	6814(2)	4290(1)	18(1)
C(18)	9522(2)	6846(2)	3848(2)	26(1)
C(19)	8333(2)	7048(3)	5360(2)	31(1)
C(14)	6397(2)	5522(2)	4216(1)	17(1)
C(15)	7033(2)	4260(2)	4032(1)	12(1)

Table S19. Bond lengths [Å] and angles [°] for V23078.

C(1)-O(2)	1.205(2)
C(1)-O(1)	1.345(2)
C(1)-C(2)	1.519(2)
O(1)-C(4)	1.450(2)
C(2)-C(15)	1.538(2)
C(2)-C(3)	1.558(2)
C(2)-H(2)	1.0000
C(3)-C(4)	1.562(2)
C(3)-C(7)	1.567(2)
C(3)-H(3)	1.0000
C(4)-C(5)	1.526(3)
C(4)-H(4)	1.0000
C(5)-C(6)	1.514(3)
C(5)-H(5A)	0.9900
C(5)-H(5B)	0.9900
C(6)-O(3)	1.464(2)
C(6)-C(16)	1.519(3)
C(6)-C(7)	1.538(2)
C(16)-H(16A)	0.9800
C(16)-H(16B)	0.9800
C(16)-H(16C)	0.9800
O(3)-C(9)	1.440(2)
C(7)-C(8)	1.502(3)
C(7)-H(7)	1.0000
C(8)-O(4)	1.210(2)
C(8)-C(9)	1.524(2)
C(9)-C(10)	1.532(2)
C(9)-H(9)	1.0000
C(10)-C(11)	1.532(3)
C(10)-C(15)	1.552(2)
C(10)-H(10)	1.0000
C(11)-O(5)	1.213(3)
C(11)-C(12)	1.510(3)

C(12)-C(13)	1.540(3)
C(12)-H(12A)	0.9900
C(12)-H(12B)	0.9900
C(13)-C(17)	1.521(3)
C(13)-C(14)	1.536(2)
C(13)-H(13)	1.0000
C(17)-C(18)	1.320(3)
C(17)-C(19)	1.507(3)
C(18)-H(18A)	0.9500
C(18)-H(18B)	0.9500
C(19)-H(19A)	0.9800
C(19)-H(19B)	0.9800
C(19)-H(19C)	0.9800
C(14)-C(15)	1.539(2)
C(14)-H(14A)	0.9900
C(14)-H(14B)	0.9900
C(15)-H(15)	1.0000
O(2)-C(1)-O(1)	120.78(16)
O(2)-C(1)-C(2)	128.23(17)
O(1)-C(1)-C(2)	110.92(15)
C(1)-O(1)-C(4)	111.62(14)
C(1)-C(2)-C(15)	113.41(14)
C(1)-C(2)-C(3)	104.63(14)
C(15)-C(2)-C(3)	121.73(15)
C(1)-C(2)-H(2)	105.2
C(15)-C(2)-H(2)	105.2
C(3)-C(2)-H(2)	105.2
C(2)-C(3)-C(4)	102.99(14)
C(2)-C(3)-C(7)	120.57(14)
C(4)-C(3)-C(7)	104.81(14)
C(2)-C(3)-H(3)	109.3
C(4)-C(3)-H(3)	109.3
C(7)-C(3)-H(3)	109.3
O(1)-C(4)-C(5)	108.84(16)

O(1)-C(4)-C(3)	107.71(15)
C(5)-C(4)-C(3)	106.80(15)
O(1)-C(4)-H(4)	111.1
C(5)-C(4)-H(4)	111.1
C(3)-C(4)-H(4)	111.1
C(6)-C(5)-C(4)	105.65(15)
C(6)-C(5)-H(5A)	110.6
C(4)-C(5)-H(5A)	110.6
C(6)-C(5)-H(5B)	110.6
C(4)-C(5)-H(5B)	110.6
H(5A)-C(5)-H(5B)	108.7
O(3)-C(6)-C(5)	108.81(15)
O(3)-C(6)-C(16)	111.30(17)
C(5)-C(6)-C(16)	114.30(16)
O(3)-C(6)-C(7)	100.67(14)
C(5)-C(6)-C(7)	104.23(16)
C(16)-C(6)-C(7)	116.41(15)
C(6)-C(16)-H(16A)	109.5
C(6)-C(16)-H(16B)	109.5
H(16A)-C(16)-H(16B)	109.5
C(6)-C(16)-H(16C)	109.5
H(16A)-C(16)-H(16C)	109.5
H(16B)-C(16)-H(16C)	109.5
C(9)-O(3)-C(6)	108.09(13)
C(8)-C(7)-C(6)	99.87(14)
C(8)-C(7)-C(3)	115.22(14)
C(6)-C(7)-C(3)	103.31(14)
C(8)-C(7)-H(7)	112.5
C(6)-C(7)-H(7)	112.5
C(3)-C(7)-H(7)	112.5
O(4)-C(8)-C(7)	129.37(16)
O(4)-C(8)-C(9)	125.40(17)
C(7)-C(8)-C(9)	105.15(15)
O(3)-C(9)-C(8)	104.91(14)
O(3)-C(9)-C(10)	109.12(14)

C(8)-C(9)-C(10)	109.92(14)
O(3)-C(9)-H(9)	110.9
C(8)-C(9)-H(9)	110.9
C(10)-C(9)-H(9)	110.9
C(11)-C(10)-C(9)	112.76(15)
C(11)-C(10)-C(15)	111.51(14)
C(9)-C(10)-C(15)	112.63(14)
C(11)-C(10)-H(10)	106.5
C(9)-C(10)-H(10)	106.5
C(15)-C(10)-H(10)	106.5
O(5)-C(11)-C(12)	122.55(18)
O(5)-C(11)-C(10)	122.04(18)
C(12)-C(11)-C(10)	115.25(16)
C(11)-C(12)-C(13)	113.38(15)
C(11)-C(12)-H(12A)	108.9
C(13)-C(12)-H(12A)	108.9
C(11)-C(12)-H(12B)	108.9
C(13)-C(12)-H(12B)	108.9
H(12A)-C(12)-H(12B)	107.7
C(17)-C(13)-C(14)	113.26(15)
C(17)-C(13)-C(12)	113.68(16)
C(14)-C(13)-C(12)	109.33(16)
C(17)-C(13)-H(13)	106.7
C(14)-C(13)-H(13)	106.7
C(12)-C(13)-H(13)	106.7
C(18)-C(17)-C(19)	120.33(19)
C(18)-C(17)-C(13)	123.89(17)
C(19)-C(17)-C(13)	115.75(17)
C(17)-C(18)-H(18A)	120.0
C(17)-C(18)-H(18B)	120.0
H(18A)-C(18)-H(18B)	120.0
C(17)-C(19)-H(19A)	109.5
C(17)-C(19)-H(19B)	109.5
H(19A)-C(19)-H(19B)	109.5
C(17)-C(19)-H(19C)	109.5

H(19A)-C(19)-H(19C)	109.5
H(19B)-C(19)-H(19C)	109.5
C(13)-C(14)-C(15)	111.50(14)
C(13)-C(14)-H(14A)	109.3
C(15)-C(14)-H(14A)	109.3
C(13)-C(14)-H(14B)	109.3
C(15)-C(14)-H(14B)	109.3
H(14A)-C(14)-H(14B)	108.0
C(2)-C(15)-C(14)	111.08(14)
C(2)-C(15)-C(10)	112.70(14)
C(14)-C(15)-C(10)	109.96(14)
C(2)-C(15)-H(15)	107.6
C(14)-C(15)-H(15)	107.6
C(10)-C(15)-H(15)	107.6

Symmetry transformations used to generate equivalent atoms:

Table S20. Anisotropic displacement parameters (Å^{2}x 10^3) for V23078. The anisotropic displacement factor exponent takes the form: -2p^2[h^2 a*^{2}U^{11} + ... + 2 h k a* b* U^{12}]

	U^{11}	U^{22}	U^{33}	U^{23}	U^{13}	U^{12}
C(1)	12(1)	16(1)	16(1)	-1(1)	2(1)	-1(1)
O(1)	14(1)	17(1)	24(1)	1(1)	-2(1)	-3(1)
O(2)	13(1)	21(1)	25(1)	2(1)	-2(1)	0(1)
C(2)	11(1)	14(1)	14(1)	-1(1)	0(1)	-1(1)
C(3)	15(1)	14(1)	15(1)	3(1)	-2(1)	-2(1)
C(4)	17(1)	15(1)	23(1)	3(1)	-1(1)	-3(1)
C(5)	22(1)	12(1)	31(1)	-1(1)	-2(1)	-3(1)
C(6)	17(1)	13(1)	23(1)	-2(1)	-4(1)	2(1)
C(16)	22(1)	17(1)	30(1)	-8(1)	-2(1)	4(1)
O(3)	16(1)	14(1)	20(1)	-2(1)	-4(1)	1(1)
C(7)	15(1)	12(1)	20(1)	0(1)	-3(1)	2(1)
C(8)	13(1)	15(1)	15(1)	-4(1)	1(1)	3(1)
O(4)	13(1)	21(1)	21(1)	-1(1)	1(1)	0(1)
C(9)	13(1)	15(1)	15(1)	-1(1)	1(1)	1(1)
C(10)	15(1)	14(1)	14(1)	1(1)	-2(1)	1(1)
C(11)	23(1)	18(1)	14(1)	1(1)	-6(1)	-4(1)
O(5)	34(1)	28(1)	19(1)	1(1)	6(1)	-9(1)
C(12)	31(1)	15(1)	22(1)	4(1)	-7(1)	-1(1)
C(13)	20(1)	10(1)	22(1)	0(1)	-1(1)	2(1)
C(17)	23(1)	13(1)	19(1)	-1(1)	1(1)	-4(1)
C(18)	23(1)	32(1)	22(1)	-1(1)	2(1)	-7(1)
C(19)	30(1)	43(1)	20(1)	-7(1)	4(1)	-13(1)
C(14)	15(1)	12(1)	24(1)	-2(1)	2(1)	1(1)
C(15)	12(1)	10(1)	15(1)	0(1)	1(1)	-1(1)

Table S21. Hydrogen coordinates (x 10^4) and isotropic displacement parameters ($Å^2$x 10^3) for V23078.

	x	y	z	U(eq)
H(2)	6125	3546	5208	16
H(3)	7227	1873	5426	18
H(4)	5630	530	5171	22
H(5A)	6539	-683	4038	26
H(5B)	5548	-40	3313	26
H(16A)	7579	-441	2120	35
H(16B)	8505	-726	3006	35
H(16C)	8789	419	2304	35
H(7)	8646	890	4419	19
H(9)	8209	2808	2057	17
H(10)	6278	3990	2671	17
H(12A)	6463	6498	2377	27
H(12B)	7875	7026	2419	27
H(13)	6650	7361	3865	21
H(18A)	10269	7018	4204	31
H(18B)	9569	6697	3174	31
H(19A)	9168	7282	5606	47
H(19B)	8045	6293	5686	47
H(19C)	7732	7721	5482	47
H(14A)	5539	5518	3931	20
H(14B)	6309	5653	4920	20
H(15)	7907	4300	4307	15

Table S22. Torsion angles [°] for V23078.

O(2)-C(1)-O(1)-C(4)	170.04(17)
C(2)-C(1)-O(1)-C(4)	-12.7(2)
O(2)-C(1)-C(2)-C(15)	-32.8(3)
O(1)-C(1)-C(2)-C(15)	150.19(15)
O(2)-C(1)-C(2)-C(3)	-167.69(18)
O(1)-C(1)-C(2)-C(3)	15.34(18)
C(1)-C(2)-C(3)-C(4)	-11.45(17)
C(15)-C(2)-C(3)-C(4)	-141.55(16)
C(1)-C(2)-C(3)-C(7)	104.72(17)
C(15)-C(2)-C(3)-C(7)	-25.4(2)
C(1)-O(1)-C(4)-C(5)	-110.86(17)
C(1)-O(1)-C(4)-C(3)	4.6(2)
C(2)-C(3)-C(4)-O(1)	4.94(18)
C(7)-C(3)-C(4)-O(1)	-122.00(15)
C(2)-C(3)-C(4)-C(5)	121.72(16)
C(7)-C(3)-C(4)-C(5)	-5.22(19)
O(1)-C(4)-C(5)-C(6)	97.19(17)
C(3)-C(4)-C(5)-C(6)	-18.8(2)
C(4)-C(5)-C(6)-O(3)	-70.65(19)
C(4)-C(5)-C(6)-C(16)	164.26(16)
C(4)-C(5)-C(6)-C(7)	36.09(19)
C(5)-C(6)-O(3)-C(9)	148.40(15)
C(16)-C(6)-O(3)-C(9)	-84.77(18)
C(7)-C(6)-O(3)-C(9)	39.23(17)
O(3)-C(6)-C(7)-C(8)	-45.18(16)
C(5)-C(6)-C(7)-C(8)	-157.91(14)
C(16)-C(6)-C(7)-C(8)	75.23(19)
O(3)-C(6)-C(7)-C(3)	73.87(16)
C(5)-C(6)-C(7)-C(3)	-38.85(17)
C(16)-C(6)-C(7)-C(3)	-165.72(16)
C(2)-C(3)-C(7)-C(8)	19.3(2)
C(4)-C(3)-C(7)-C(8)	134.58(16)
C(2)-C(3)-C(7)-C(6)	-88.48(18)

C(4)-C(3)-C(7)-C(6)	26.75(18)
C(6)-C(7)-C(8)-O(4)	-140.98(19)
C(3)-C(7)-C(8)-O(4)	109.1(2)
C(6)-C(7)-C(8)-C(9)	35.84(17)
C(3)-C(7)-C(8)-C(9)	-74.05(17)
C(6)-O(3)-C(9)-C(8)	-16.76(18)
C(6)-O(3)-C(9)-C(10)	-134.50(15)
O(4)-C(8)-C(9)-O(3)	163.79(17)
C(7)-C(8)-C(9)-O(3)	-13.19(18)
O(4)-C(8)-C(9)-C(10)	-79.0(2)
C(7)-C(8)-C(9)-C(10)	104.00(16)
O(3)-C(9)-C(10)-C(11)	-148.60(14)
C(8)-C(9)-C(10)-C(11)	96.86(17)
O(3)-C(9)-C(10)-C(15)	84.12(17)
C(8)-C(9)-C(10)-C(15)	-30.4(2)
C(9)-C(10)-C(11)-O(5)	8.8(2)
C(15)-C(10)-C(11)-O(5)	136.61(18)
C(9)-C(10)-C(11)-C(12)	-175.67(15)
C(15)-C(10)-C(11)-C(12)	-47.8(2)
O(5)-C(11)-C(12)-C(13)	-136.2(2)
C(10)-C(11)-C(12)-C(13)	48.2(2)
C(11)-C(12)-C(13)-C(17)	75.3(2)
C(11)-C(12)-C(13)-C(14)	-52.4(2)
C(14)-C(13)-C(17)-C(18)	126.3(2)
C(12)-C(13)-C(17)-C(18)	0.7(3)
C(14)-C(13)-C(17)-C(19)	-55.8(2)
C(12)-C(13)-C(17)-C(19)	178.63(18)
C(17)-C(13)-C(14)-C(15)	-68.85(19)
C(12)-C(13)-C(14)-C(15)	59.0(2)
C(1)-C(2)-C(15)-C(14)	71.42(19)
C(3)-C(2)-C(15)-C(14)	-162.34(15)
C(1)-C(2)-C(15)-C(10)	-52.49(19)
C(3)-C(2)-C(15)-C(10)	73.76(19)
C(13)-C(14)-C(15)-C(2)	174.67(15)
C(13)-C(14)-C(15)-C(10)	-59.88(18)

C(11)-C(10)-C(15)-C(2)	177.06(14)
C(9)-C(10)-C(15)-C(2)	-55.01(19)
C(11)-C(10)-C(15)-C(14)	52.53(18)
C(9)-C(10)-C(15)-C(14)	-179.54(14)
